# Assessing the use of a micro-sampling device for measuring blood protein levels in healthy subjects and COVID-19 patients

**DOI:** 10.1371/journal.pone.0272572

**Published:** 2022-08-10

**Authors:** Joost Brandsma, Josh G. Chenoweth, Melissa K. Gregory, Subramaniam Krishnan, Paul W. Blair, Deborah A. Striegel, Rittal Mehta, Kevin L. Schully, J. Stephen Dumler, CDR Cynthia S. Sikorski, Kelsey O’Connor, Susan A. Reichert-Scrivner, Carmen M. Paguirigan, Catherine F. T. Uyehara, COL Viseth Ngauy, Christopher A. Myers, Danielle V. Clark

**Affiliations:** 1 Austere Environments Consortium for Enhanced Sepsis Outcomes, Henry M. Jackson Foundation for the Advancement of Military Medicine Inc., Bethesda, Maryland, United States of America; 2 Department of Pathology, School of Medicine, Uniformed Services University, Bethesda, Maryland, United States of America; 3 Austere Environments Consortium for Enhanced Sepsis Outcomes, Biological Defense Research Directorate, Naval Medical Research Center-Frederick, Frederick, Maryland, United States of America; 4 Naval Health Research Center, San Diego, California, United States of America; 5 Tripler Army Medical Center, Honolulu, Hawaii, United States of America; The Ohio State University, UNITED STATES

## Abstract

**Background:**

Venous phlebotomy performed by trained personnel is critical for patient diagnosis and monitoring of chronic disease, but has limitations in resource-constrained settings, and represents an infection control challenge during outbreaks. Self-collection devices have the potential to shift phlebotomy closer to the point of care, supporting telemedicine strategies and virtual clinical trials. Here we assess a capillary blood micro-sampling device, the Tasso Serum Separator Tube (SST), for measuring blood protein levels in healthy subjects and non-hospitalized COVID-19 patients.

**Methods:**

57 healthy controls and 56 participants with mild/moderate COVID-19 were recruited at two U.S. military healthcare facilities. Healthy controls donated Tasso SST capillary serum, venous plasma and venous serum samples at multiple time points, while COVID-19 patients donated a single Tasso SST serum sample at enrolment. Concentrations of 17 protein inflammatory biomarkers were measured in all biospecimens by Ella multi-analyte immune-assay.

**Results:**

Tasso SST serum protein measurements in healthy control subjects were highly reproducible, but their agreements with matched venous samples varied. Most of the selected proteins, including CRP, Ferritin, IL-6 and PCT, were well-correlated between Tasso SST and venous serum with little sample type bias, but concentrations of D-dimer, IL-1B and IL-1Ra were not. Self-collection at home with delayed sample processing was associated with significant concentrations differences for several analytes compared to supervised, in-clinic collection with rapid processing. Finally, Tasso SST serum protein concentrations were significantly elevated in in non-hospitalized COVID-19 patients compared with healthy controls.

**Conclusions:**

Self-collection of capillary blood with micro-sampling devices provides an attractive alternative to routine phlebotomy. However, concentrations of certain analytes may differ significantly from those in venous samples, and factors including user proficiency, temperature control and time lags between specimen collection and processing need to be considered for their effect on sample quality and reproducibility.

## Introduction

Blood molecular biomarkers are critical for patient diagnosis, prognosis and monitoring of chronic disease. The “gold standard” method of blood collection is venous phlebotomy, but this is subject to certain limitations and potential complications, including availability of trained personnel, patients needing to travel to a central location, fear of needles, vasovagal responses, and phlebotomist exposure to potentially infectious bodily fluids or other transmissible infections. Deployment of self-collection devices for blood micro-sampling overcomes most of these limitations, shifting disease diagnosis and population monitoring closer to the point-of-care [1]. These technologies have the potential to revolutionize health care delivery by supporting telehealth visits and virtual clinical trials, providing conveniences to the patient and enabling outreach to remote populations. Additionally, self-collection devices can facilitate the rapid collection of blood during mass population visits, for example during medical in-processing at military recruitment accession sites.

While the U.S. Food and Drug Administration (FDA) in 2018 recommended comparison studies between venous blood and micro-samples of capillary blood, few such studies have been conducted to date [2, 3]. As a consequence, challenges remain in integrating results obtained using either sampling approach. Here we present a direct comparison between capillary blood micro-sampling and routine venous phlebotomy for protein biomarker analysis in an outpatient setting, and within the context of enabling virtual clinical trials for SARS-CoV-2 and other diseases.

The Tasso Serum Separator Tube (SST) (Tasso Inc., WA, USA) is a single-use, sterile, disposable, integrated device for self-collection of capillary blood by the user [4]. It is classified as an FDA 510(k) Class 2 device when used for a specific diagnostic panel, but is otherwise for investigational use only (IUO). The device comprises a lancet assembly and a detachable reservoir collection unit (called Tasso Button), designed to collect up to 300 μL capillary blood that can be processed to generate serum at a central processing lab for downstream analysis [5–7]. We quantified 17 protein inflammatory biomarkers in Tasso SST capillary serum and phlebotomy samples (venous serum and plasma), obtained from healthy subjects and non-hospitalized COVID-19 patients recruited at two U.S. military healthcare facilities. The inflammatory biomarkers were selected from previously published immune signatures in COVID-19 or sepsis [8], or based on their clinical use in the management of COVID-19 [9]. Using matched samples, our primary aim was to assess the utility of capillary blood micro-sampling as an alternative to routine phlebotomy. Our secondary aim was to examine the impact of unsupervised self-collection at home with the Tasso SST device, versus supervised collection and rapid processing in the clinic. Finally, we demonstrate the utility of blood self-sampling for telemedicine by testing whether the Tasso SST serum protein biomarkers differed significantly between non-hospitalized mild/moderate COVID-19 patients and healthy controls.

## Materials and methods

113 participants were enrolled under the PROMETHEUS 2.0 protocol between April 2020 and January 2021 at two U.S. military healthcare facilities: Tripler Army Medical Center in Honolulu, Hawaii (TAMC) and the Naval Health Research Centre in San Diego, California (NHRC) (S1 Table). Informed consent and/or assent was received from all participants before enrolment was completed. While written, paper-based consent was available for use, due to the contagious nature of emergent infectious diseases being studied, study staff were not always able to collect in-person consent from potential participants. In such cases, an electronic version with an alteration of the informed consent was utilized. For participants between 13–17 years of age, participant assent and parental/guardian consent was obtained. Participant COVID-19 status was established by RT-PCR test prior to enrolment, and subsequently monitored by either further RT-PCR tests or GeneXpert Xpress SARS-CoV-2 tests (Cepheid, Sunnyvale, CA, USA) of nasopharyngeal swabs collected throughout the study (up to five swabs in total, coinciding with the blood collection). All healthy controls were considered high-risk for contracting COVID-19 due to their profession or otherwise close proximity to COVID-19 patients (*e*.*g*., healthcare workers, family members).

**Fig 1:**
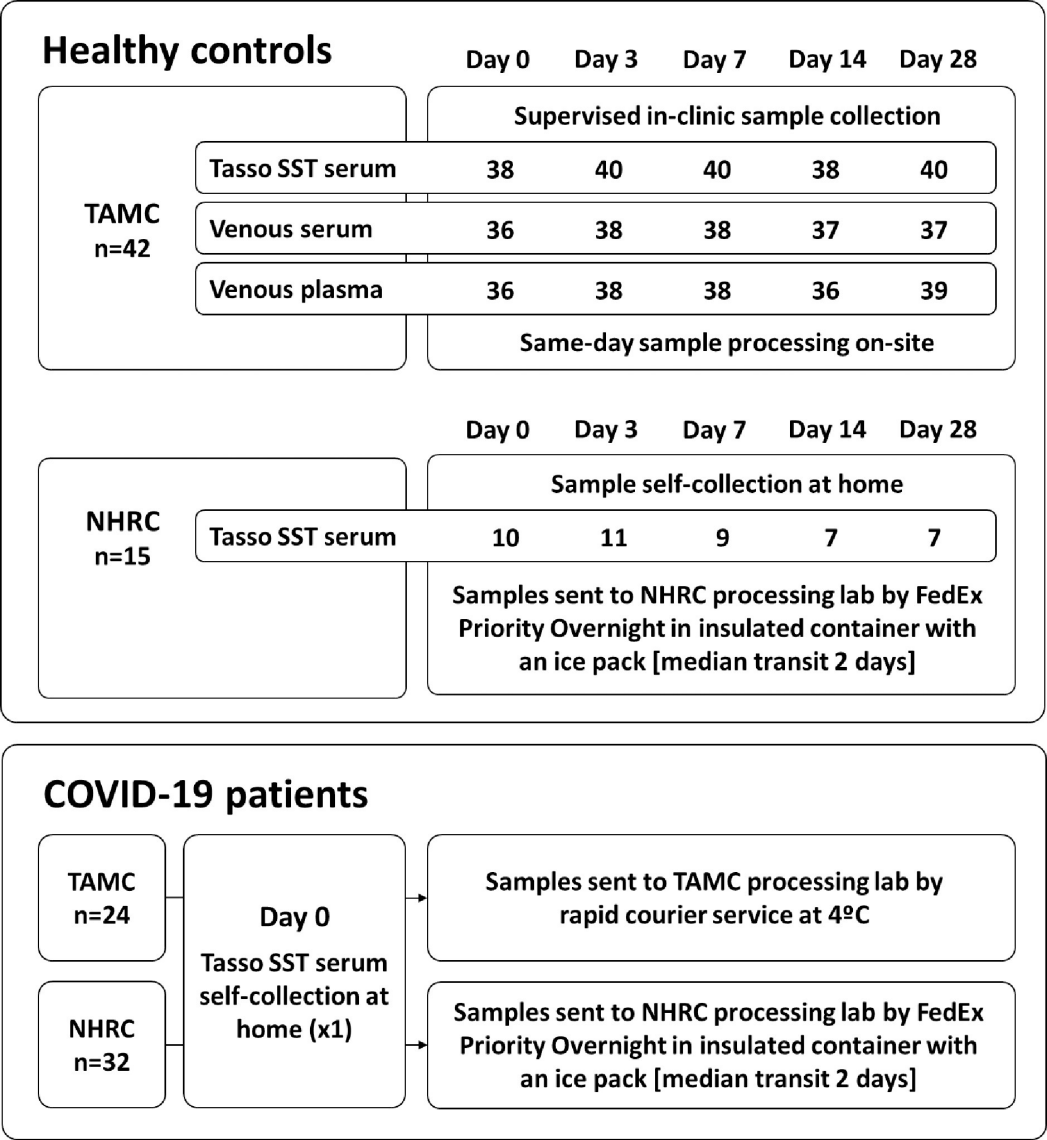
Flow chart of the samples collected at each site, the sampling location (unsupervised self-collection at home, or supervised collection in-clinic), and method of transport to the central processing labs. TAMC: Tripler Army Medical Center; NHRC: Naval Health Research Center.

Forty-two (42) healthy controls recruited at TAMC donated up to 5 matched samples of venous serum (clot-activator vacutainer), venous plasma (K2EDTA), and capillary blood (Tasso SST), at 0, 3-, 7-, 14- and 28-days post-enrolment (Fig 1). These samples were collected under supervision at the clinic and processed the same day, allowing for a direct comparison between the three sample types. Fifteen (15) healthy controls recruited at NHRC self-collected up to 5 capillary blood samples (Tasso SST) at home on the same study time points. These samples were sent from the participants’ home address to the NHRC lab by FedEx Priority Overnight in insulated containers with an ice pack, with transit times ranging from 0–5 days (median 2 days). Thus, the study design allowed for comparison of Tasso SST specimens processed shortly after supervised collection versus self-collected specimens with potentially delayed processing, albeit with unmatched samples. Median collection volumes for the Tasso SST after serum processing were 112 μl and 120 μl for the at-home and in-clinic collections, respectively, and recorded incidences of device failures and sample haemolysis were both very low (<1%).

Fifty-six (56) participants tested positive for SARS-CoV-2 at enrolment (24 TAMC and 32 NHRC), and 1 of the TAMC healthy controls contracted COVID-19 partway through the study, thus switching study arms (Fig 1). These COVID-19 patients self-isolated at home and collected a capillary blood sample (Tasso SST) at enrolment or on day-0 of study arm reassignment (n = 57). TAMC samples were sent cold (4°C) by rapid courier service directly to the specimen processing site (total transit time 0–3 days, median 1 day), while the NHRC samples were again sent with an ice pack by regular courier (total transit time 0–7 days, median 2 days).

A panel of 17 protein biomarkers of inflammation was measured in triplicate in all samples using the Ella multi-analyte immuno-assay (ProteinSimple, San Jose, CA, USA). Concentrations of CD163, CRP, CXCL10, D-dimer, Ferritin, ICAM-1, IL-1B, IL-1Ra, IL-5, IL-6, IL-6Ra, IL-18BPa, LCN, PCT, RAGE, TNF-R1 and VEGF-A were log_10_-transformed, and any measurements below the lower limit of detection (LLoD) of the Ella platform were imputed using the lowest measured value for that particular analyte. This only affected IL-1B and IL-5 (40% and 14% imputation, respectively). Eight percent (8%) of the D-dimer measurements were above the upper limit of detection (ULoD) for the assay, and these were imputed using the highest measured value for D-dimer. Finally, a small amount of missing data (<1% for any given analyte) was imputed using a *k*-nearest neighbour algorithm in Python (“sklearn” package version 0.24.2, *k* = 4). Protein concentrations and supporting data used in this study are provided in the Supplementary Information.

All plots and statistics were generated in R (version 3.6.3) using the “ggpubr” package (version 0.4.0). Unless otherwise stated, the n reported in the tables and figures refers to individual samples (up to 5 per subject). Comparisons across multiple groups were done by Kruskal-Wallis test, and comparisons between two specific groups using the Mann–Whitney U test (adjusted q-values are reported). Scatter plots were fitted with a simple linear regression, and we report the Pearson correlation coefficient of determination (R^2^). Fixed bias in the Bland-Altman plots was assessed by paired t-test.

## Results

Reproducibility of the Tasso SST assay was assessed by calculating the coefficient of variation (CV) for each protein between the study time points of each healthy control subject, and determining the median and range. Irrespective of sampling and shipment protocol, we found excellent reproducibility (median CV <5%) for most of the tested analytes. Exceptions were IL-1B (median CV 79%), IL-5 (median CV 47%), and IL-6 (median CV 63%), all of which were at the lower limit of the Ella platform’s dynamic range in these healthy subjects.

Protein concentrations in Tasso SST serum samples from the TAMC healthy controls (supervised, in-clinic collection) were compared to those in matched venous serum and plasma samples (Table 1; S1 Fig). For this comparison, samples from all study time points were used to cover the full baseline concentration range in a non-diseased population. Protein concentrations were comparable across all three sample types for CD163, CRP, ICAM-1, IL-6, IL-6Ra, IL-18BPa, PCT and RAGE. Levels of CXCL10, Ferritin, IL-1Ra, TNF-R1 and VEGF-A were significantly higher in Tasso SST serum samples than in both venous serum and plasma. IL-5 and LCN concentrations were comparable in both serum types, but significantly different in venous plasma. In contrast, levels of D-dimer and IL-1Ra were comparable for Tasso SST serum and venous plasma, but significantly lower in venous serum. For each of the tested analytes, concentrations in capillary serum sampled by Tasso SST were equal to or higher than those found in venous serum.

**Table 1:** Comparison of protein concentrations from peripheral blood samples of 42 TAMC healthy controls obtained in-clinic using the Tasso SST (capillary serum) and phlebotomy (venous serum and plasma). Up to 5 samples were collected from each participant over a 28-day period and are aggregated here. Concentrations are given as the median and interquartile range, and sorted by adjusted Kruskal-Wallis test q-values. *Note the high proportion of samples with IL-1B and IL-5 concentrations below the LLoQ. Boxplots of individual proteins with between-group significance are given in S1 Fig.

Protein concentrations in matched samples from healthy controls					
TAMC in-clinic supervised collection—all time points					
Analyte	N	Tasso SST serum	Venous serum	Venous plasma	KW test
**CRP**	183	0.80 μg/ml (0.39–1.82)	0.86 μg/ml (0.41–2.04)	0.87 μg/ml (0.41–1.97)	0.932
**CD163**	152	431 ng/ml (314–557)	431 ng/ml (309–569)	404 ng/ml (294–547)	0.662
**IL-6**	183	1.48 pg/ml (0.89–2.36)	1.36 pg/ml (0.87–2.13)	1.35 pg/ml (0.90–2.09)	0.662
**IL-18BPa**	152	4.28 ng/ml (3.77–5.61)	4.46 ng/ml (3.93–5.98)	4.38 ng/ml (3.79–5.78)	0.577
**PCT**	183	60.8 pg/ml (40.4–80.7)	56.4 pg/ml (37.6–79.3)	56.1 pg/ml (37.5–80.0)	0.497
**RAGE**	183	0.91 ng/ml (0.75–1.13)	0.95 ng/ml (0.78–1.18)	0.97 pg/ml (0.77–1.22)	0.392
**ICAM-1**	183	292 ng/ml (248–340)	301 ng/ml (264–349)	294 ng/ml (254–344)	0.338
**IL-6Ra**	183	45.4 ng/ml (38.4–52.0)	47.1 ng/ml (40.2–53.0)	48.1 ng/ml (40.2–54.2)	0.113
**Ferritin**	183	101 ng/ml (60–161)	76 ng/ml (41–148)	74 ng/ml (37–139)	**0.004**
**LCN**	183	76.6 ng/ml (64.1–90.4)	76.4 ng/ml (66.2–87.7)	70.8 ng/ml (60.8–78.0)	**4.4E-05**
**CXCL10**	152	111 pg/ml (86–164)	107 pg/ml (74–150)	87 pg/ml (57–122)	**3.3E-08**
IL-5*	183	0.13 pg/ml (0–0.28)	0.11 pg/ml (0–0.26)	0.25 pg/ml (0.12–0.45)	**7.5E-09**
**TNF-R1**	183	0.97 ng/ml (0.82–1.10)	0.89 ng/ml (0.77–1.02)	0.82 pg/ml (0.72–0.96)	**1.4E-10**
IL-1B*	183	0.02 pg/ml (0–0.44)	0 pg/ml (0–0)	0.02 pg/ml (0–0.13)	**3.4E-11**
**D-dimer**	183	0.22 μg/ml (0.35–0.44)	0.13 μg/ml (0.09–0.23)	0.26 μg/ml (0.17–0.39)	**2.7E-13**
**VEGF-A**	183	140 pg/ml (89–236)	104 pg/ml (60–182)	29 pg/ml (23–40)	**2.1E-64**
**IL-1Ra**	183	1.58 ng/ml (1.12–2.14)	0.26 ng/ml (0.20–0.37)	0.21 ng/ml (0.15–0.31)	**1.7E-78**

**Fig 2:**
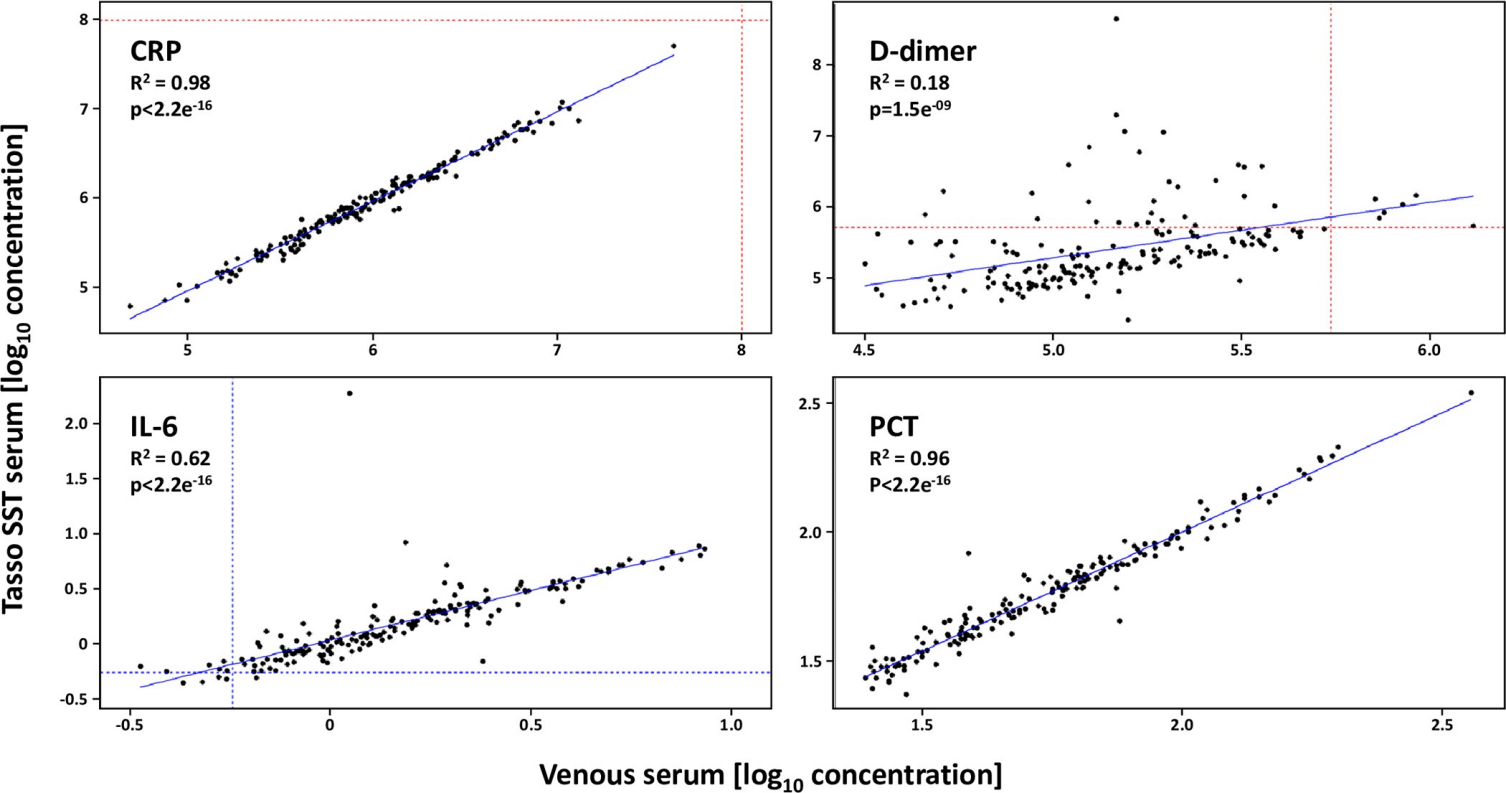
Correlation of selected protein concentrations in matched peripheral blood samples of 42 TAMC healthy controls obtained in-clinic using the Tasso SST (capillary serum) and phlebotomy (venous serum). Up to 5 samples were collected from each participant over a 28-day period and are aggregated here. Concentrations were log10 transformed. Scatter plots were fitted with a simple linear regression (solid blue line), and the Pearson correlation R and p values are shown. Ella assay limits of detection are show with the dashed lines (blue: LLoD; red: ULoD).

Using the matched sample set from TAMC healthy controls, we also assessed whether protein concentrations were correlated between the three sample types (selected plots shown in Fig 2; see S2 Table and S2 Fig for the full results). Most of the tested analytes had either excellent (R^2^>0.9) or good (R^2^ = 0.6–0.9) correlations across all sample types, including CD163, CRP, CXCL10, Ferritin, ICAM-1, IL-6, IL-6Ra, IL-18BPa, PCT, RAGE and TNF-R1. Correlations for IL-5, LCN and VEGF-A were moderate (R^2^ = 0.4–0.6), not just for the comparison between Tasso SST serum and venous samples, but also when comparing matched venous serum and plasma. Finally, there was no correlation (R^2^<0.2) for D-dimer, IL-1B and IL-1Ra, although only IL-1B was also poorly correlated between venous serum and plasma. Bland-Altman plots showed only minor fixed biases between sample types for most of the analytes (S2 Table and S3 Fig). Exceptions were the analytes at the low end of the Ella platform’s dynamic range (IL-1B, IL-5 and IL-6), as well as a bias towards Tasso SST serum for IL-1Ra, and towards either serum type for VEGF-A and CXCL10.

Protein concentrations in the Tasso SST serum of healthy controls were compared between those with supervised, in-clinic collection and prompt specimen processing (TAMC; 196 samples from 42 subjects), and those with self-collection at home and delayed specimen processing due to longer transit times (NHRC; 44 samples from 15 subjects). Levels of D-dimer, IL-1B, IL-1Ra, IL-5, IL-6Ra, LCN and PCT were all significantly different in samples from the two protocols, and particularly D-dimer and IL-1B had much higher median concentrations in the self-collected samples (Table 2). Levels of CXCL10, ICAM-1 and IL-18BPa were also significantly different in the self-collected samples, but the concentration ranges for these analytes were comparable for both protocols, and we note the small sample size for the self-collected group (NHRC).

**Table 2:** Concentrations of selected proteins in Tasso SST serum samples from healthy controls recruited at TAMC (supervised collection in-clinic with prompt processing) and NHRC (at-home self-collection with delayed processing). Up to 5 samples were collected from each subject over a 28-day period and are aggregated here. Concentrations are given as the median and interquartile range, with Mann-Whitney U test q-values for comparing the two sites. *Note the high proportion of at-home, self-collected samples with D-dimer concentrations above the ULoQ.

Healthy control Tasso SST serum—Comparison between sampling protocols			
	Clinic supervised	Home self-collection	MW U test
Analyte	TAMC (n = 196)	NHRC (n = 44)	q-value
**Ferritin**	100 ng/ml (59–158)	72 ng/ml (52–212)	0.707
**CRP**	0.88 μg/ml (0.43–2.04)	1.18 μg/ml (0.37–2.64)	0.568
**CD163**	433 ng/ml (322–566)	457 ng/ml (373–531)	0.442
**TNF-R1**	0.97 ng/ml (0.83–1.11)	0.99 ng/ml (0.84–1.18)	0.442
**RAGE**	0.91 ng/ml (0.75–1.14)	1.01 ng/ml (0.76–1.24)	0.396
**VEGF-A**	153 pg/ml (91–244)	196 pg/ml (112–279)	0.118
**IL-6**	1.53 pg/ml (0.91–2.39)	1.70 pg/ml (1.46–2.32)	0.080
**ICAM-1**	290 ng/ml (248–339)	261 ng/ml (249–294)	**0.027**
**IL-18BPa**	4.27 ng/ml (3.78–5.56)	4.05 ng/ml (3.27–4.57)	**0.009**
**CXCL10**	113 pg/ml (88–167)	97 pg/ml (83–130)	**0.007**
**IL-1Ra**	1.57 ng/ml (1.11–2.09)	2.24 ng/ml (1.74–4.12)	**6.3E-06**
**IL-6Ra**	45.2 ng/ml (37.7–51.9)	34.1 ng/ml (28.2–44.2)	**3.1E-06**
**LCN**	77.1 ng/ml (64.9–93.3)	106.7 ng/ml (81.1–162.9)	**2.1E-06**
**PCT**	60.8 pg/ml (40.7–81.3)	37.8 pg/ml (33.7–44.8)	**4.5E-07**
**IL-5**	0.13 pg/ml (0–0.28)	0.39 pg/ml (0.22–0.64)	**1.2E-08**
D-dimer*	0.23 μg/ml (0.12–0.48)	12.19 μg/ml (5.61–463.5)	**1.9E-15**
**IL-1B**	0.02 pg/ml (0–0.44)	2.30 pg/ml (0.95–4.25)	**1.9E-15**

We compared Tasso SST serum protein concentrations at baseline (day-0) of 48 healthy controls and 57 mild/moderate COVID-19 patients (all biospecimens were self-collected at-home, but with delayed processing for the 32 NHRC samples). The median duration between date of infection (first confirmed COVID-19 RT-PCR result) and baseline sampling was 5 days (range 0–16 days). Concentrations of CD163, CRP, CXCL10, D-dimer, Ferritin, IL-1B, IL-1Ra, IL-6, LCN, TNF-R1 and VEGF-A were all significantly higher in the COVID-positive samples (Table 3). However, increased levels of *e*.*g*., D-dimer, IL-1B, IL-1Ra and IL-5 in the healthy control samples that were self-collected at home resulted in different outcomes for the individual sampling protocols (S4 Fig).

**Table 3:** Baseline concentrations of selected proteins in Tasso SST serum samples from healthy controls and COVID-19 patients (pooled data from both study sites). Concentrations are given as the median and interquartile range, and sorted by adjusted Mann-Whitney U test q-values. *Note the high proportion of samples with D-dimer concentrations above the ULoQ, particularly in the self-collected protocol (NHRC). Boxplots for each analyte are given in S4 Fig.

Comparison between Tasso SST serum protein concentrations of healthy controls and COVID-19 patients at enrolment			
	Healthy controls	COVID-19	MW U test
Analyte	n = 48	n = 57	q-value
D-dimer*	0.40 μg/ml (0.15–2.55)	6.82 μg/ml (1.45–26.36)	**4.3E-06**
**IL-1B**	0.02 pg/ml (0–0.88)	1.67 pg/ml (0.32–13.00)	**4.3E-06**
**IL-6**	1.64 pg/ml (1.04–2.55)	5.67 pg/ml (1.72–34.60)	**1.9E-04**
**CXCL10**	110 pg/ml (84–162)	194 pg/ml (127–454)	**2.0E-04**
**VEGF-A**	152 pg/ml (91–231)	295 pg/ml (185–372)	**2.4E-04**
**TNF-R1**	1.00 ng/ml (0.81–1.09)	1.14 ng/ml (0.98–1.26)	**2.5E-04**
**LCN**	82.0 ng/ml (69.5–106.9)	116.9 ng/ml (86.9–223.4)	**3.2E-04**
**CRP**	1.03 μg/ml (0.38–2.33)	2.59 μg/ml (1.01–5.06)	**0.003**
**IL-1Ra**	1.58 ng/ml (1.13–2.17)	2.53 ng/ml (1.48–4.03)	**0.005**
**Ferritin**	97 ng/ml (58–159)	136 ng/ml (76–331)	**0.034**
**CD163**	449 ng/ml (347–579)	536 ng/ml (430–693)	**0.035**
**ICAM-1**	302 ng/ml (261–358)	327 ng/ml (284–378)	0.118
**IL-5**	0.16 pg/ml (0–0.30)	0.21 pg/ml (0–0.35)	0.118
**IL-18BPa**	4.23 ng/ml (3.85–5.41)	4.82 ng/ml (4.13–5.53)	0.141
**PCT**	50.9 pg/ml (39.0–69.7)	44.8 pg/ml (32.1–68.2)	0.202
**RAGE**	1.00 ng/ml (0.73–1.16)	0.87 ng/ml (0.75–1.12)	0.826
**IL-6Ra**	45.8 ng/ml (35.7–52.0)	42.6 ng/ml (35.2–51.1)	0.972

## Discussion

Self-collection of blood micro-samples overcomes a number of limitations associated with regular blood collection in-clinic, providing conveniences to the patient and enabling outreach to difficult-to-access populations [2]. By shifting phlebotomy closer to the point of care, devices such as the Tasso SST have an important role to play in the development of telemedicine strategies and conducting virtual clinical trials. Additionally, they can facilitate the rapid collection of blood during mass population visits, such as medical in-processing at military recruit accession sites. Here we assess the Tasso SST, a capillary blood micro-sampling device, for measuring blood protein levels in healthy subjects and mild/moderate COVID-19 patients in an outpatient setting. We show that Tasso SST-collected capillary serum measurements of multiple clinically relevant inflammatory biomarkers, including CRP, Ferritin, IL-6 and PCT, are highly reproducible and correlate well with routine phlebotomy samples (venous serum). Furthermore, we demonstrate that Tasso SST serum protein levels in samples from COVID-19 patients differ significantly from those obtained from healthy controls.

Capillary blood micro-sampling is mostly utilized for neonates and infants, when only small volumes are available or when venous access is difficult, but in adults it is an uncommon method for blood collection. The Tasso SST is classified as an FDA 510(k) Class 2 device when used for a specific diagnostic panel, but otherwise the technology is for investigational use only (IUO). Our primary aim was to assess the reproducibility of Tasso SST serum protein measurements, as well as their agreement with protein levels measured in matched venous serum and plasma samples. High reproducibility is important for any clinically-relevant assay, be it well-established or newly-developed. Repeat sampling of healthy controls over a period of 28 days showed excellent within-subject reproducibility for most of the protein biomarkers. Measurements of IL-1B, IL-5 and IL-6 were more variable, but this could be ascribed to low circulating levels of these analytes in the absence of disease. Protein concentrations in the Tasso SST serum were equal to, or higher than, those measured in matched venous samples. However, the smaller sample volumes obtained using this technology (median 112–120 μL) may be a limiting factor in some study designs. It was further noted that user training and experience with the devices prior to deployment had a positive impact on sample yield, and that users preferred this technology to devices that use fingersticks (VN, personal observations).

The reasons for the observed concentration differences between capillary and venous serum samples are not entirely clear, since we were unable to ascertain whether there are location-specific differences in healthy adults due to a paucity of comparative proteomics measurements [4]. Prior studies have found that capillary and venous blood differ significantly in their haematological measurements, cell counts, and coagulation factors [10, 11], as well as the levels of some specific protein biomarkers (*e*.*g*., S100B for intracranial haemorrhage) [12]. Hence, it stands to reason that several of the analytes included in our inflammatory protein panel would differ as well. Our results suggest that baseline levels of D-dimer, IL-1Ra, Ferritin, TNF-R1 and VEGF-A are higher in the capillary serum of healthy adults than in venous serum. Future work may validate these observations, or expand on them by performing a more comprehensive proteomic analysis.

Substituting one sampling method or biospecimen type for another requires a high level of agreement between the two, ideally in the form of a linear relationship between the analyte yields over a wide range of concentrations. Most of the tested proteins showed good to excellent correlations between Tasso SST serum and venous serum with little sample type bias (S2 Table). This included acute phase reactant biomarkers in current clinical use, such as CRP, ferritin, and PCT. In contrast, the lack of any correlation in D-dimer and IL-1Ra measurements makes Tasso SST-derived measurements not useful as a direct correlate of phlebotomy samples for those analytes, although it remains possible that they follow comparable trends over time during an inflammatory response to infection. Accurate agreement levels for IL-1B, IL-5 and IL-6 were difficult to establish due to their low concentrations. These analytes may turn out to be well-correlated in patients with an ongoing inflammatory response, and moderate correlations observed for some of the other analytes could likewise improve with the inclusion of samples with elevated inflammatory protein levels (*e*.*g*., LCN, VEGF-A, TNF-R1). Our results show that protein levels in self-collected samples from devices like the Tasso SST may differ from those in routine phlebotomy samples, and that normal value ranges for analytes in capillary micro-samples may need to be redefined for clinical use.

Assay reproducibility can be a significant challenge in serum cytokine analysis, in particular when comparing sample handling protocols that are employed at individual sites or for different studies [13]. We therefore examined whether unsupervised self-collection at home with potentially delayed biospecimen processing (healthy controls recruited at NHRC) impacted analyte concentrations, using the supervised in-clinic collection and rapid processing at the TAMC site as a reference. Concentrations of D-dimer, IL-1B, IL-1Ra, IL-5, IL-6Ra, LCN and PCT were all significantly different in the self-collected samples, suggesting they were potentially affected by factors such as user proficiency with the device, temperature during storage and shipping, or transit times. We note that the relatively small sample size of the self-collected group (44 samples from 15 NHRC healthy controls) may have contributed to the observed differences (*e*.*g*., CXCL10, ICAM-1 and IL-18BPa), and that we did not control for potential demographic differences between the two study sites. Given the high within-patient reproducibility in this study, it is unlikely that the observed differences between NHRC and TAMC samples were caused by the way Tasso devices were applied and handled by individual participants. Sample transit times were recorded, but protein concentrations did not show significant associations with delays in sample processing (linear regression or Jonckheere-Terpstra test for categorized data). Unfortunately, the study design did not allow for further, more in-depth assessment of assay reproducibility under a controlled set of real-life conditions. Future biomarker studies involving micro-sampling devices should build on our results and are recommended to include rigorous, analyte-specific reproducibility testing and validation prior to enrolment. Because of the unique sampling technology involved, as well as the potential differences between venous and capillary blood, such testing will ideally be performed with actual devices, rather than on bulk commercial blood products. This will ensure selection of analytes that are robust to differences in sample handling protocols, as well as variable environmental conditions and user proficiency.

To demonstrate the clinical utility of blood self-sampling for telemedicine (*e*.*g*., virtual clinical trials, distributed medicine), we tested whether the Tasso SST serum protein biomarkers could distinguish COVID-19 patients from healthy controls in a cross-sectional manner. Although none of the COVID-19 cases enrolled in this study were severe enough to require hospitalization, their Tasso SST serum contained significantly elevated levels of a range of protein biomarkers. These results are in line with the published literature on COVID-19 [8, 14–20] and inflammatory markers in general (*e*.*g*., CRP, Ferritin, D-dimer), although we acknowledge that the COVID-19 patients were recruited from a diverse demographic at two different sites, and included a range of disease stages (days after onset) and severities. Ongoing work by our group includes mapping the longitudinal inflammatory responses in these patients using the capillary micro-sampling approach, and how those biomarker patterns can be used to predict clinical outcomes. Cytokines and chemokines are involved in the effector phase of all inflammatory diseases, and many have been implicated in the pathobiology of COVID-19, or proposed as prognostic markers of disease severity or hospital/ICU admission [8, 14–20]. As COVID-19 progresses over time, disruption of cellular processes leads to increased biological dissonance, cascading to overt symptoms and a variety of possible disease phenotypes, which depend on the interplay between pathogen and host response [21, 22]. Early detection, diagnosis and treatment of infection have been shown to result in significant reductions in disease severity and mortality, including in COVID-19 [23, 24]. The ability to remotely monitor disease progression after the initial diagnosis, and time interventions accordingly, is likely to yield additional benefits, in particular for high-risk individuals and those early on in the disease when the risk of transmission is highest [21]. In this context, unsupervised self-collection of Tasso SST capillary blood for Anti-SARS-CoV-2 IgG antibody testing, and mid-nasal swabs for detection of SARS-CoV-2 infection have already yielded promising results [25, 26]. In this study, we have demonstrated the utility of remote serum collection with the Tasso SST to enable detection of elevated biomarkers of inflammation in the disease state of COVID-19. In conclusion, continued development of scalable, easy-to-use technologies for at-home biological sampling and testing will permit the rapid triaging of a significant number of individuals to early care, thereby decreasing the disease burden.

## Supporting information

S1 Table Summary demographics of the study cohort. Gender is shown as the ratio of male (M) to female (F) participants; Age is given as the median and range; Race is shown as the ratio of white (W) to other/non-white (O) participants.
(PDF)

S2 Table Protein correlations between sample type. Agreement and bias for protein concentrations in matched peripheral blood sample types of TAMC healthy controls (supervised, in-clinic collection). The table lists the Linear Model coefficients of determination (Pearson’s R^2^) and Bland-Altman fixed bias. Where significant according to a paired t-test, the proportional fixed bias (%) is given; note that this exaggerates small apparent bias at low concentration ranges. *Samples with concentrations below the LLoQ were excluded for IL-1B and IL-5, resulting in a reduced sample size. Correlation and Bland-Altman plots for individual proteins are given in S2 and S3 Figs, respectively.
(PDF)

S1 Data Study data summary. Summary table of the data and metadata used in this study, including: Ella protein concentrations (in pg/ml), sample information (sample type and study time point), and selected subject information (sex, race, age, study site and COVID-19 status at the time of sampling).
(XLSX)

S1 Fig Boxplots of protein concentrations in different blood sample types from healthy controls. Comparison of protein concentrations from peripheral blood samples of 42 TAMC healthy controls obtained in-clinic using the Tasso SST (capillary serum) and phlebotomy (venous serum and plasma). Up to 5 samples were collected from each participant over a 28-day period and are aggregated here. Concentrations were log10 transformed. Significance values for Mann-Whitney U tests between sample types are abbreviated as follows: n.s. not significant; * p<0.05; ** p<0.01; *** p<0.001; **** p<0.0001.
(PDF)

S2 Fig Correlation of protein concentrations in matched venous and Tasso SST serum samples from healthy controls. Correlation of protein concentrations in matched peripheral blood samples of 42 TAMC healthy controls obtained in-clinic using the Tasso SST (capillary serum) and phlebotomy (venous serum). Up to 5 samples were collected from each participant over a 28-day period and are aggregated here. Concentrations were log10 transformed. Scatter plots were fitted with a simple linear regression (blue line), and the Pearson correlation R and *p* values are shown.
(PDF)

S3 Fig Agreement and bias of protein concentrations in matched venous and Tasso SST serum samples from healthy controls. Bland-Altman plots of protein concentrations in matched peripheral blood samples of 42 TAMC healthy controls obtained in-clinic using the Tasso SST (capillary serum) and phlebotomy (venous serum). Up to 5 samples were collected from each participant over a 28-day period and are aggregated here. Concentrations were log10 transformed. Fixed bias in the plots was assessed by paired t-test.
(PDF)

S4 Fig Tasso SST protein concentrations in COVID-19 patients and healthy controls. Baseline concentrations of selected proteins in Tasso SST serum samples from healthy controls and non-hospitalized COVID-19 patients. Data are shown for the supervised, in-clinic collection protocol at TAMC (with rapid processing), the unsupervised, at-home self-collection protocol at NHRC (with potentially delayed processing), and for both protocols combined. Concentrations were log10 transformed. Significance values for Mann-Whitney U tests between COVID-19 patients and healthy controls are abbreviated as follows: n.s. not significant; * p<0.05; ** p<0.01; *** p<0.001; **** p<0.0001.
(PDF)
