# Supplementary material for: Assessing the use of a micro-sampling device for measuring blood protein levels in healthy subjects and COVID-19 patients
Source: PLoS One. 2022 Aug 10;17(8):e0272572. doi: 10.1371/journal.pone.0272572 (PMC9365123; doi:10.1371/journal.pone.0272572)
Supplement: S1 Table — Gender is shown as the ratio of male (M) to female (F) participants; Age is given as the median and range; Race is shown as the ratio of white (W) to other/non-white (O) participants. (PDF) [file pone.0272572.s001.pdf]

	Healthy control		COVID-19	
	TAMC	NHRC	TAMC	NHRC
**Gender**	15M/26F	7M/8F	18M/6F	16M/16F
**Age**	35 (20-64)	36 (22-53)	29 (19-59)	32 (15-71)
**Race**	26W/14O	11W/4O	12W/12O	22W/10O
