# Supplementary material for: Assessing the use of a micro-sampling device for measuring blood protein levels in healthy subjects and COVID-19 patients
Source: PLoS One. 2022 Aug 10;17(8):e0272572. doi: 10.1371/journal.pone.0272572 (PMC9365123; doi:10.1371/journal.pone.0272572)
Supplement: S2 Table — Agreement and bias for protein concentrations in matched peripheral blood sample types of TAMC healthy controls (supervised, in-clinic collection). The table lists the Linear Model coefficients of determination (Pearson’s R2) and Bland-Altman fixed bias. Where significant according to a paired t-test, the proportional fixed bias (%) is given; note that this exaggerates small apparent bias at low concentration ranges. *Samples with concentrations below the LLoQ were excluded for IL-1B and IL-5, resulting in a reduced sample size. Correlation and Bland-Altman plots for individual proteins are given in S2 and S3 Figs, respectively. (PDF) [file pone.0272572.s002.pdf]

Agreement and bias in matched samples from healthy controls<br>TAMC in-clinic supervised collection - all time points							
		Tasso SST serum vs Venous serum		Tasso SST serum vs Venous plasma		Venous serum vs Venous plasma	
**Analyte**	**N**	**R^2**	**Fixed bias**	**R^2**	**Fixed bias**	**R^2**	**Fixed bias**
**CRP**	183	0.98	Tasso SST lower (<1%)	0.98	Not significant	0.98	Serum higher (<1%)
**PCT**	183	0.96	Tasso SST higher (1%)	0.96	Tasso SST higher (1%)	0.98	Not significant
**Ferritin**	183	0.92	Tasso SST higher (2%)	0.92	Tasso SST higher (3%)	0.98	Serum higher (<1%)
**IL-18BPa**	152	0.90	Tasso SST lower (<1%)	0.88	Not significant	0.90	Serum higher (<1%)
**CD163**	152	0.88	Not significant	0.88	Tasso SST higher (<1%)	0.92	Serum higher (<1%)
**CXCL10**	152	0.88	Tasso SST higher (3%)	0.86	Tasso SST higher (7%)	0.90	Serum higher (5%)
**RAGE**	183	0.81	Tasso SST lower (<1%)	0.79	Tasso SST lower (1%)	0.92	Not significant
**IL-6Ra**	183	0.76	Tasso SST lower (<1%)	0.76	Tasso SST lower (<1%)	0.77	Serum lower (<1%)
**ICAM-1**	183	0.74	Tasso SST lower (<1%)	0.72	Not significant	0.81	Serum higher (<1%)
**TNF-R1**	183	0.64	Tasso SST higher (1%)	0.61	Tasso SST higher (2%)	0.64	Serum higher (1%)
**IL-6**	183	0.62	Tasso SST higher (29%)	0.63	Tasso SST higher (21%)	0.89	Not significant
**VEGF-A**	183	0.59	Tasso SST higher (7%)	0.36	Tasso SST higher (37%)	0.42	Serum higher (30%)
**LCN**	183	0.53	Not significant	0.61	Tasso SST higher (1%)	0.67	Serum higher (1%)
**IL-5**	152*	0.41	Not significant	0.34	Tasso SST lower (40%)	0.64	Serum lower (50%)
**D-dimer**	183	0.18	Tasso SST higher (5%)	0.14	Not significant	0.79	Serum lower (5%)
**IL-1B**	24*	0.12	Not significant	0.04	Tasso SST higher (83%)	0.38	Serum higher (58%)
**IL-1Ra**	183	0.02	Tasso SST higher (27%)	0.06	Tasso SST higher (31%)	0.66	Serum higher (4%)
