# Supplementary material for: Assessing the use of a micro-sampling device for measuring blood protein levels in healthy subjects and COVID-19 patients
Source: PLoS One. 2022 Aug 10;17(8):e0272572. doi: 10.1371/journal.pone.0272572 (PMC9365123; doi:10.1371/journal.pone.0272572)
Supplement: S1 Fig — Comparison of protein concentrations from peripheral blood samples of 42 TAMC healthy controls obtained in-clinic using the Tasso SST (capillary serum) and phlebotomy (venous serum and plasma). Up to 5 samples were collected from each participant over a 28-day period and are aggregated here. Concentrations were log10 transformed. Significance values for Mann-Whitney U tests between sample types are abbreviated as follows: n.s. not significant; * p<0.05; ** p<0.01; *** p<0.001; **** p<0.0001. (PDF) [file pone.0272572.s004.pdf]

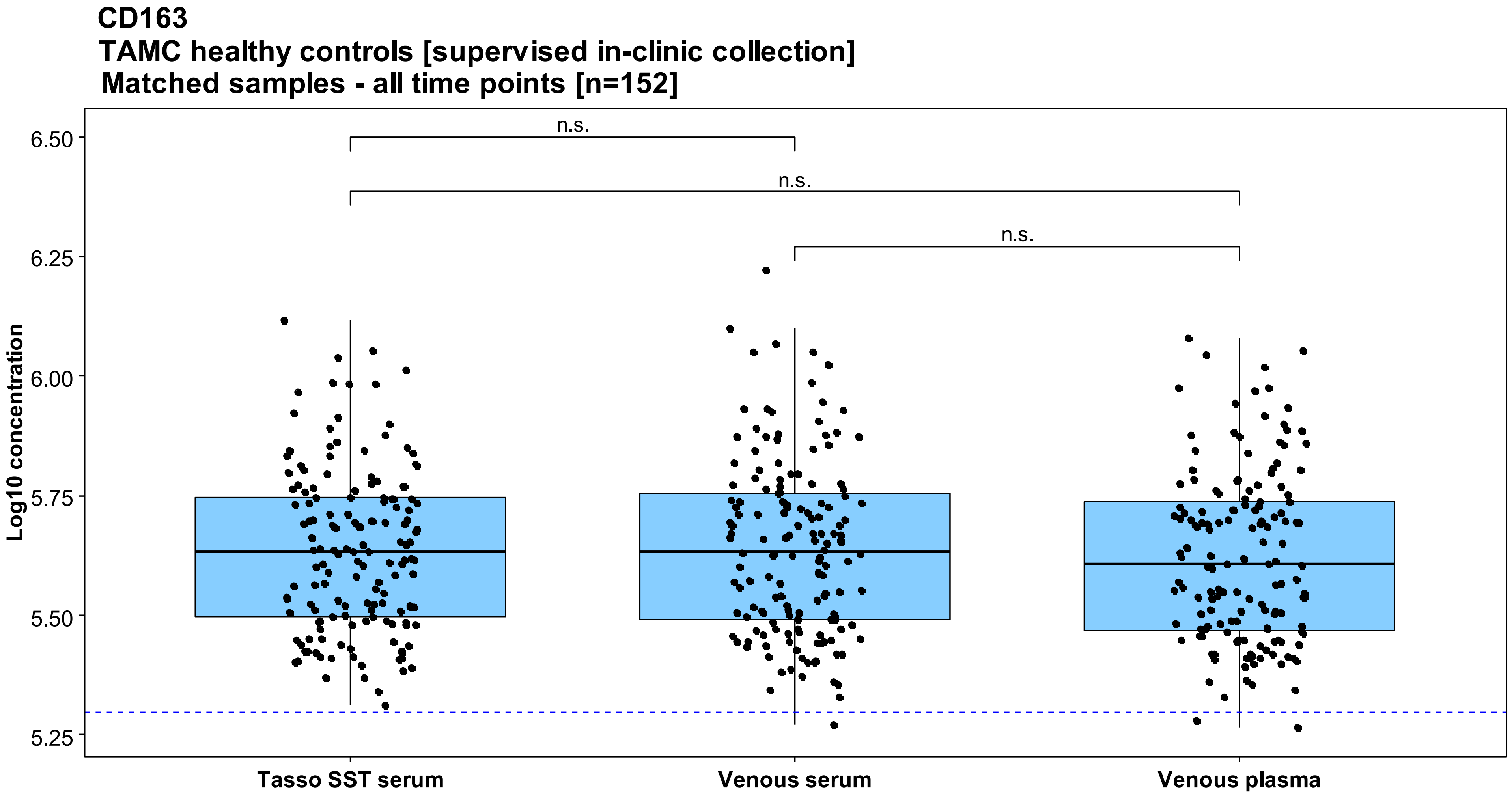
CD163
TAMC healthy controls [supervised in-clinic collection]
Matched samples - all time points [n=152]
Log10 concentration
6.50
6.25
6.00
5.75
5.50
5.25
n.s.
n.s.
n.s.
Tasso SST serum
Venous serum
Venous plasma

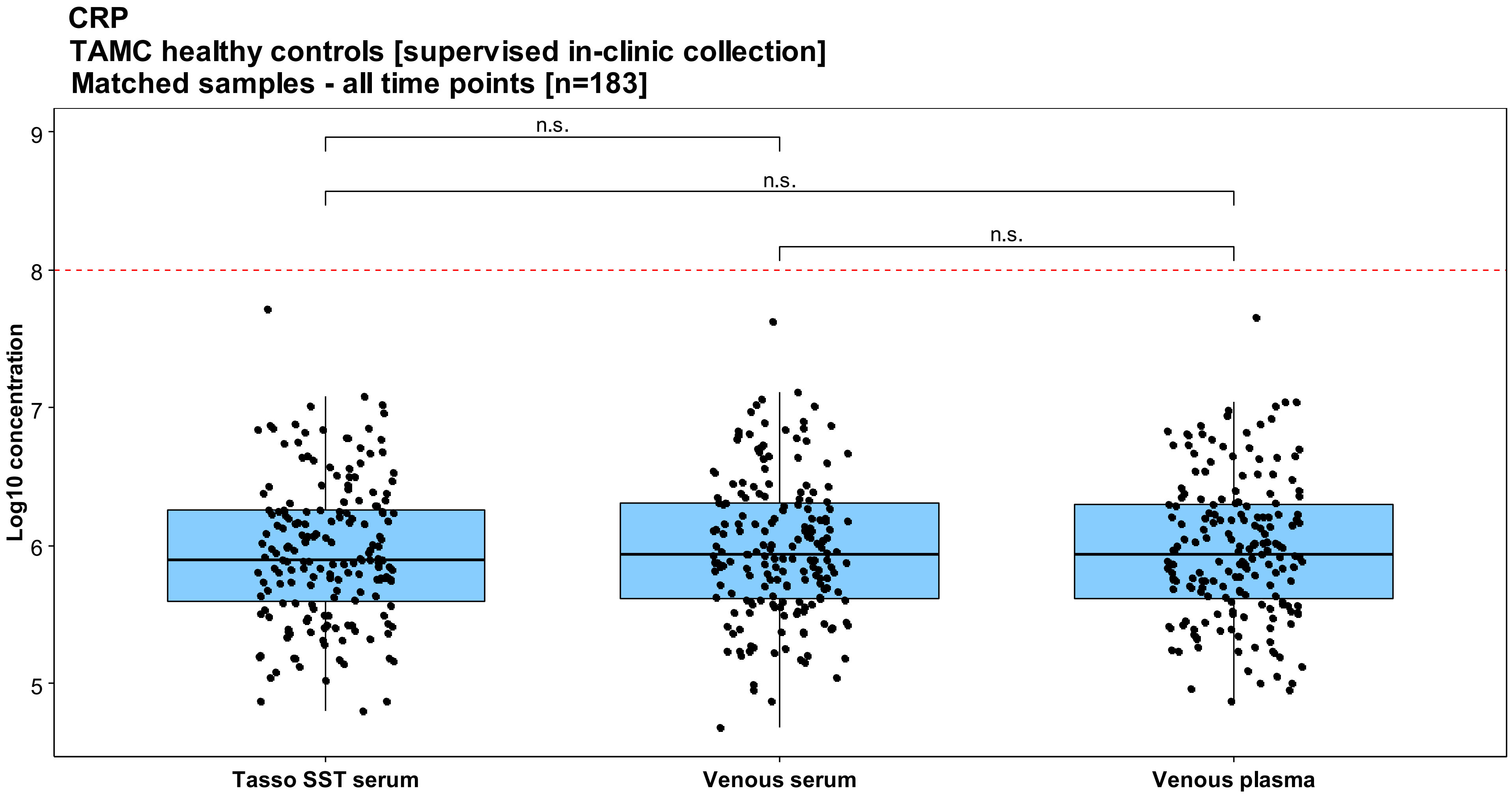

CRP
TAMC healthy controls [supervised in-clinic collection]
Matched samples - all time points [n=183]
n.s.
n.s.
n.s.
9
8
7
6
5
Log10 concentration
Tasso SST serum
Venous serum
Venous plasma

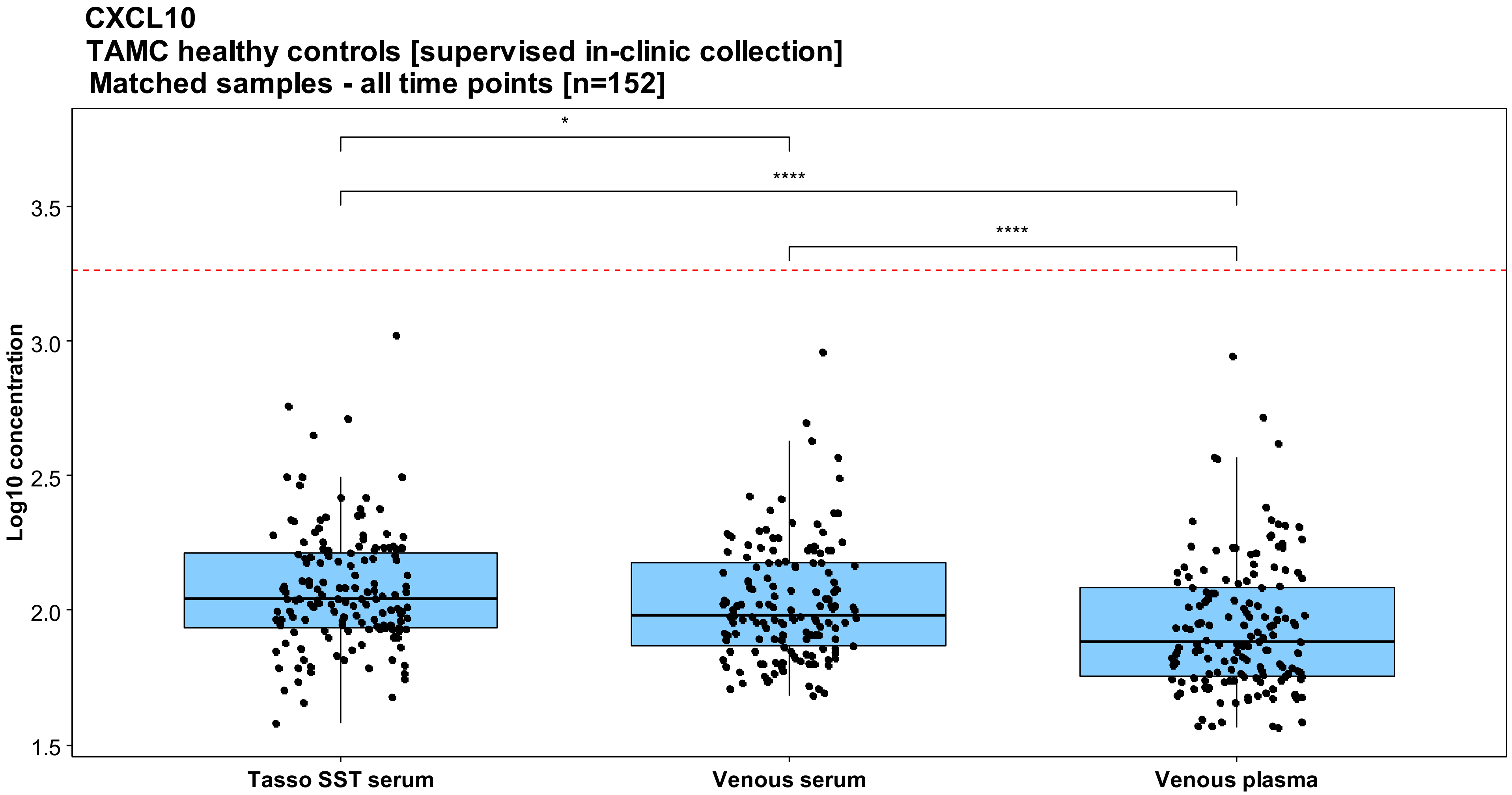

CXCL10
TAMC healthy controls [supervised in-clinic collection]
Matched samples - all time points [n=152]
*
****
****
Log10 concentration
3.5
3.0
2.5
2.0
1.5
Tasso SST serum
Venous serum
Venous plasma

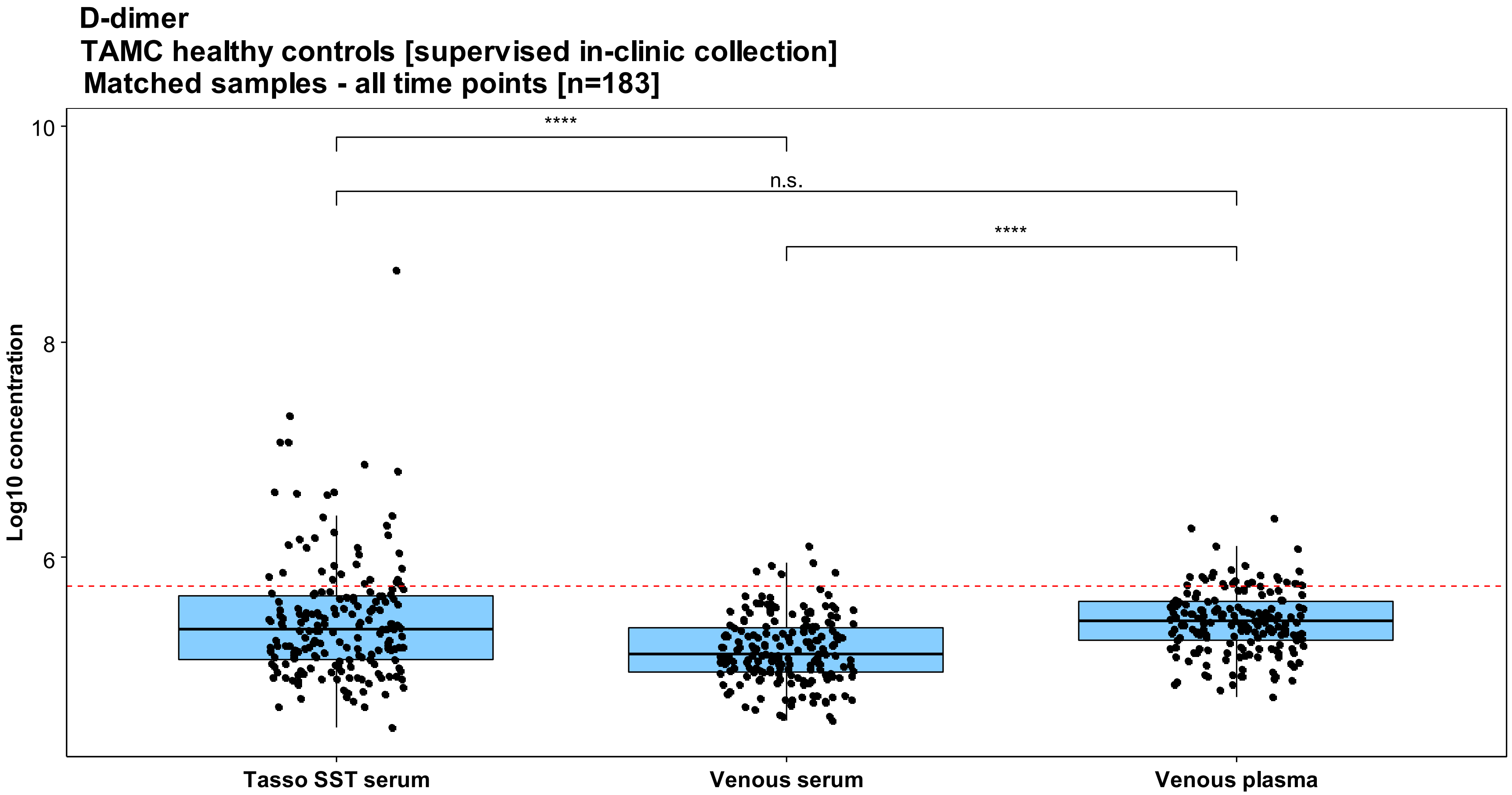
D-dimer
TAMC healthy controls [supervised in-clinic collection]
Matched samples - all time points [n=183]
****
n.s.
****
10
8
6
Log10 concentration
Tasso SST serum
Venous serum
Venous plasma

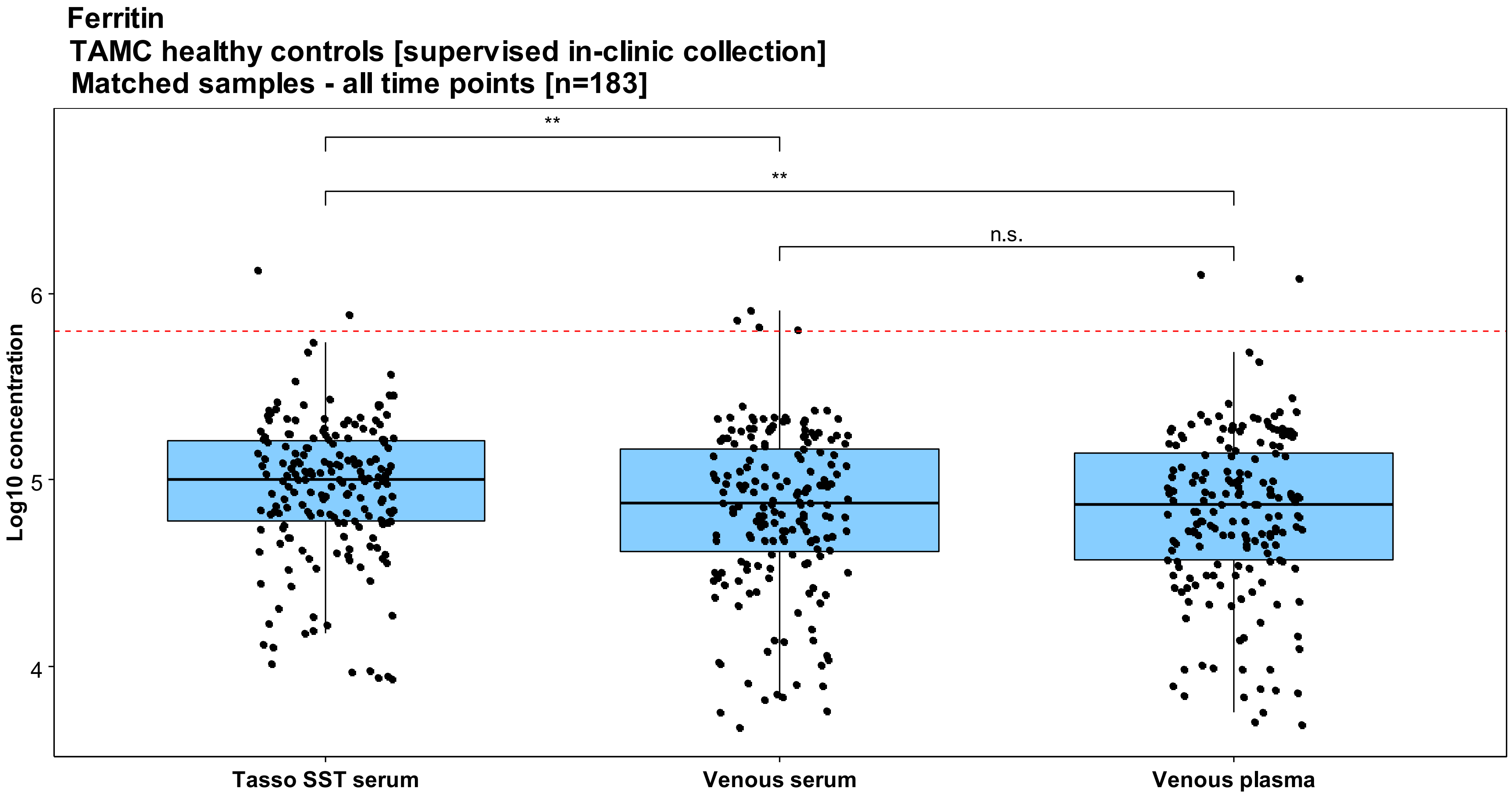

Ferritin
TAMC healthy controls [supervised in-clinic collection]
Matched samples - all time points [n=183]
**
**
n.s.
6
5
4
Log10 concentration
Tasso SST serum
Venous serum
Venous plasma

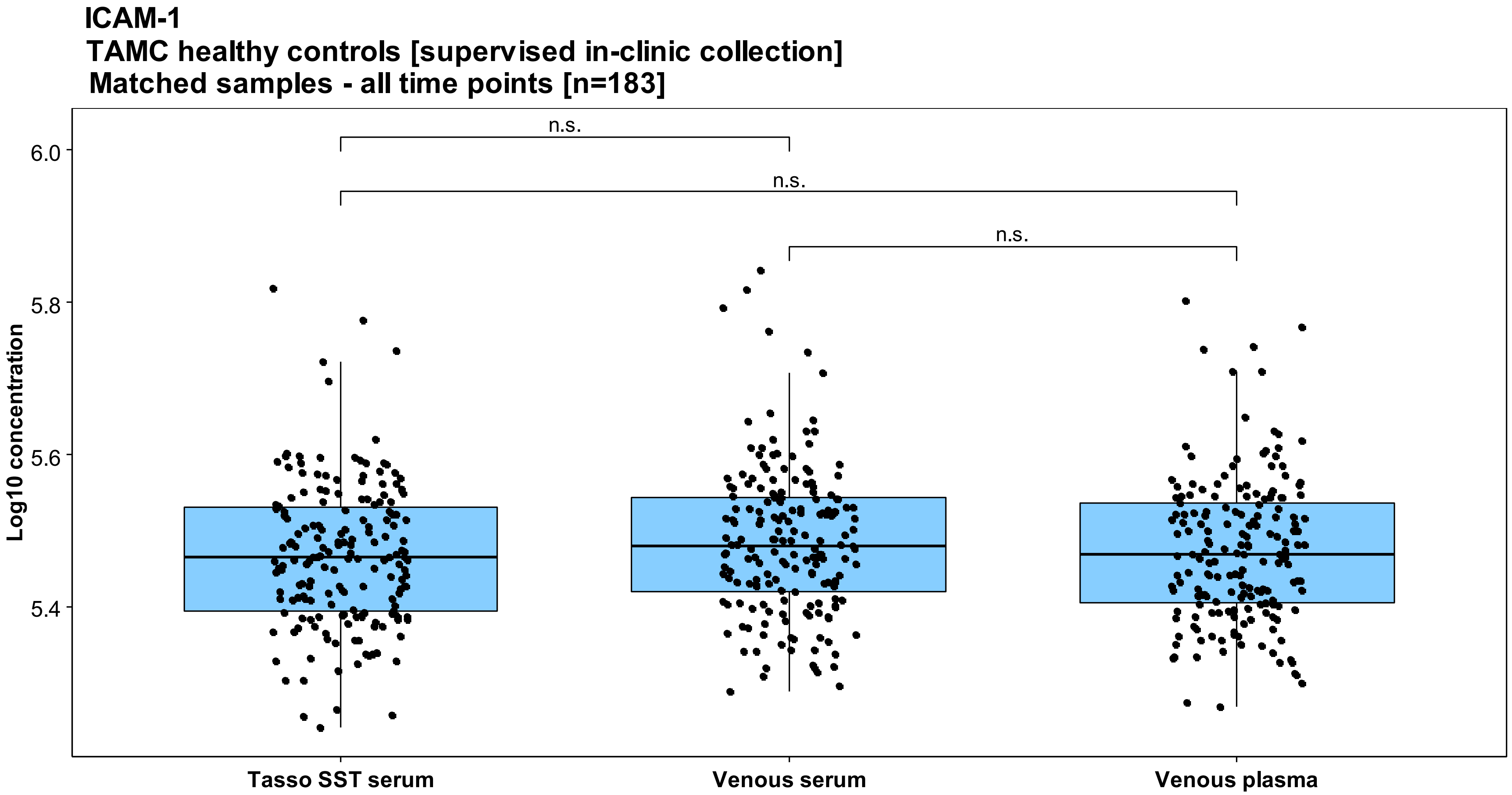

ICAM-1
TAMC healthy controls [supervised in-clinic collection]
Matched samples - all time points [n=183]
n.s.
n.s.
n.s.
Log10 concentration
6.0
5.8
5.6
5.4
Tasso SST serum
Venous serum
Venous plasma

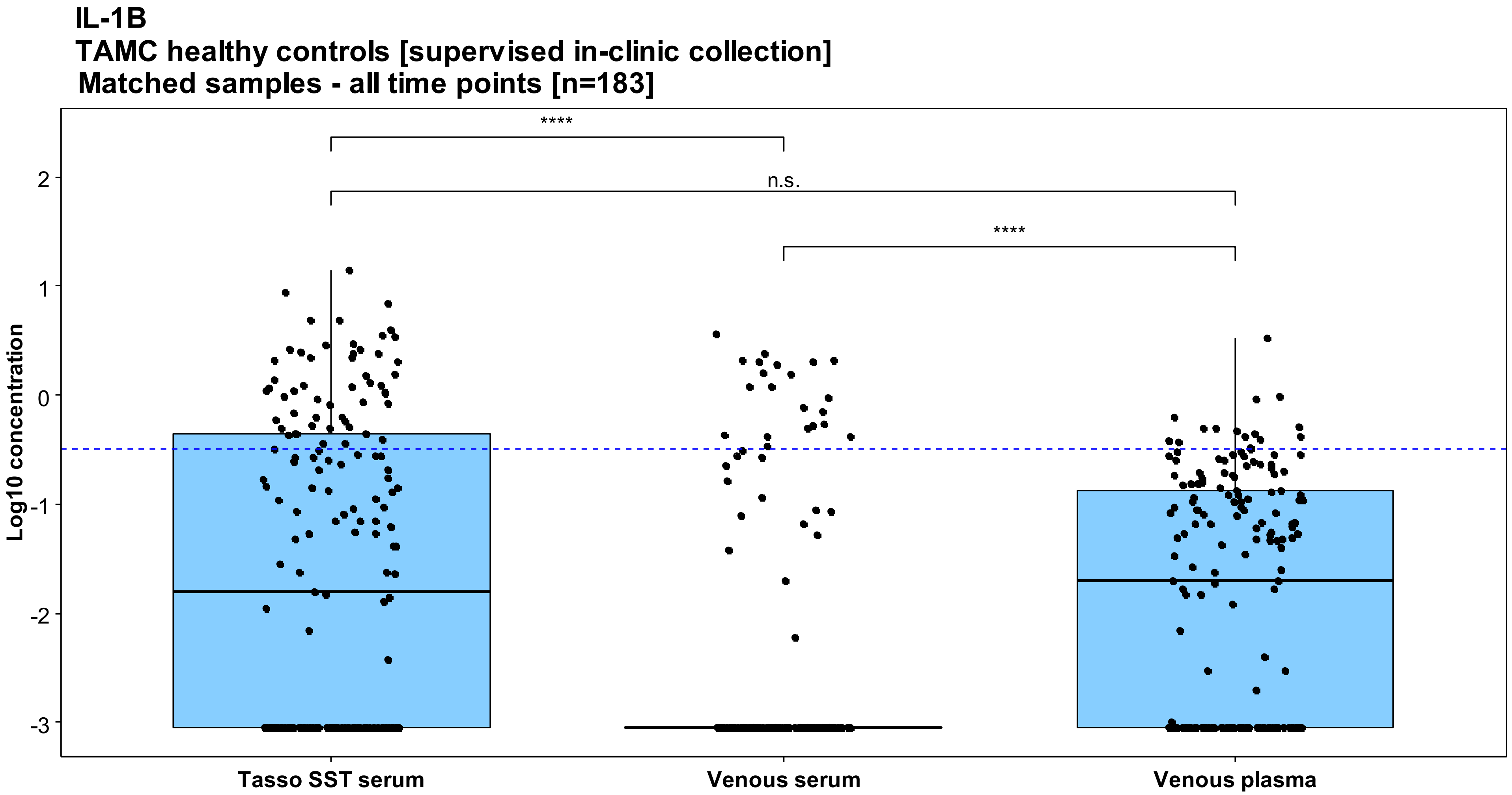
IL-1B
TAMC healthy controls [supervised in-clinic collection]
Matched samples - all time points [n=183]
****
n.s.
****
2
1
0
-1
-2
-3
Log10 concentration
Tasso SST serum
Venous serum
Venous plasma

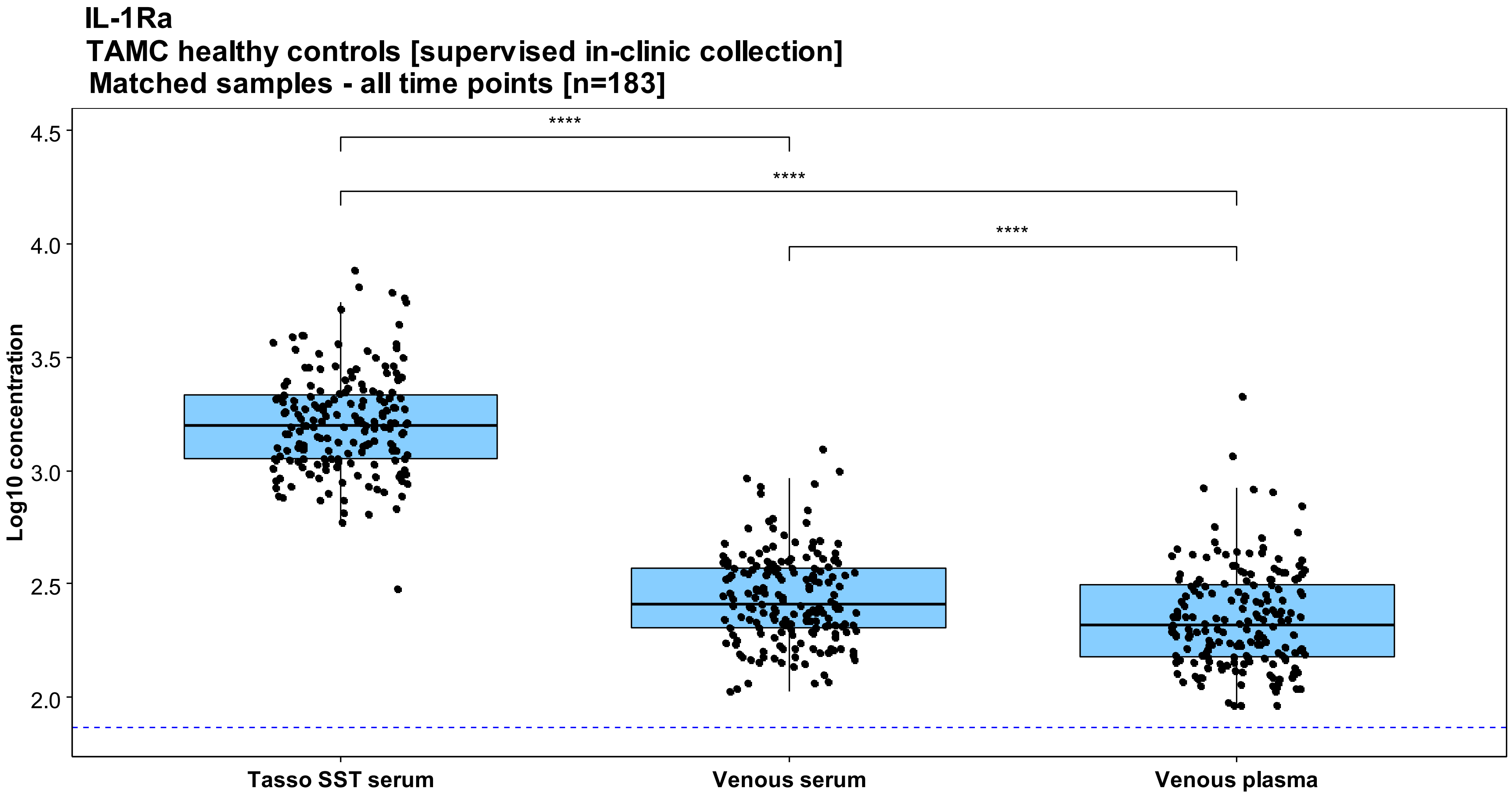

IL-1Ra
TAMC healthy controls [supervised in-clinic collection]
Matched samples - all time points [n=183]
Log10 concentration
4.5
4.0
3.5
3.0
2.5
2.0
****
****
****
Tasso SST serum
Venous serum
Venous plasma

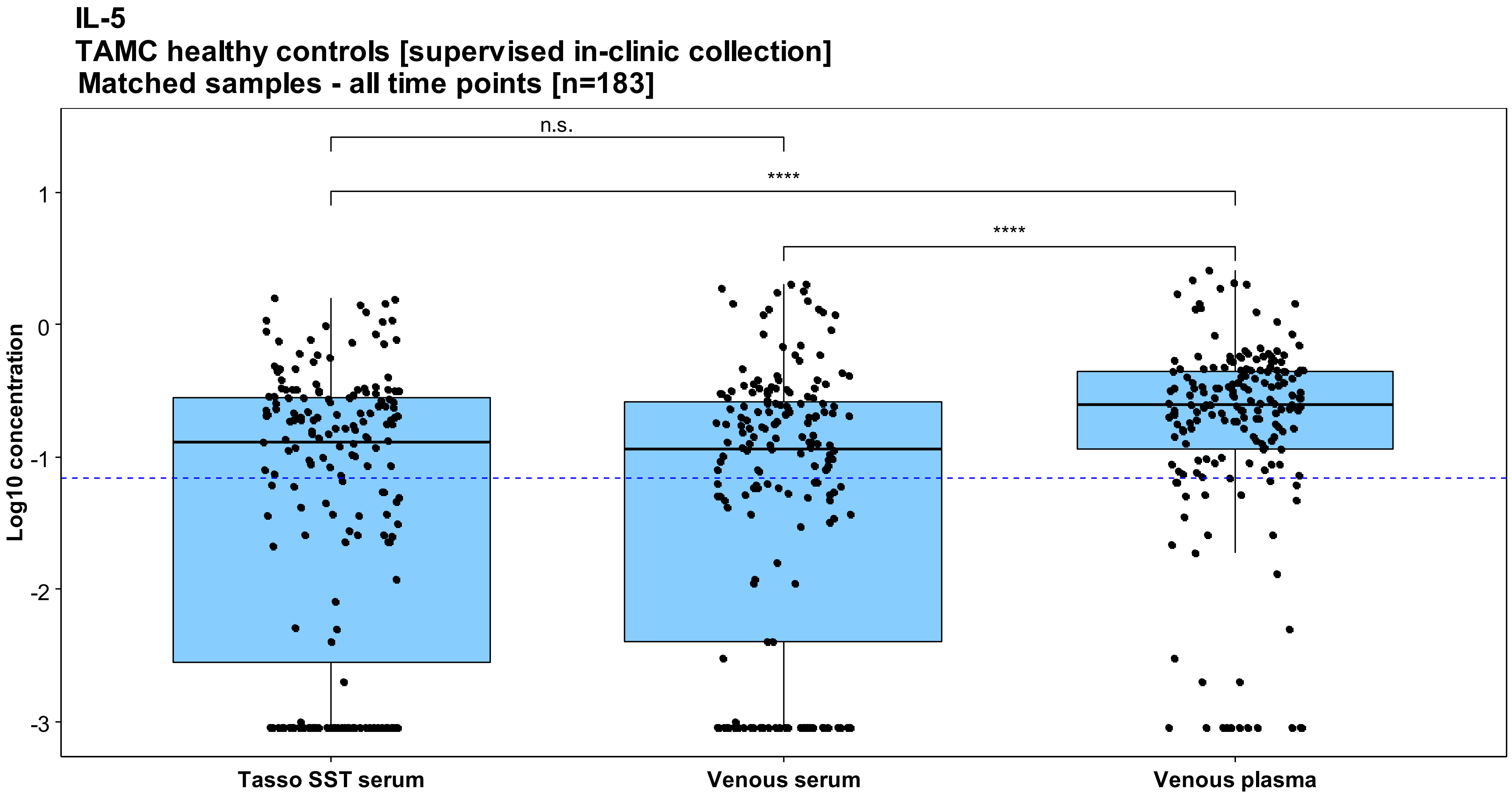
IL-5
TAMC healthy controls [supervised in-clinic collection]
Matched samples - all time points [n=183]
n.s.
****
****
1
0
-1
-2
-3
Log10 concentration
Tasso SST serum
Venous serum
Venous plasma

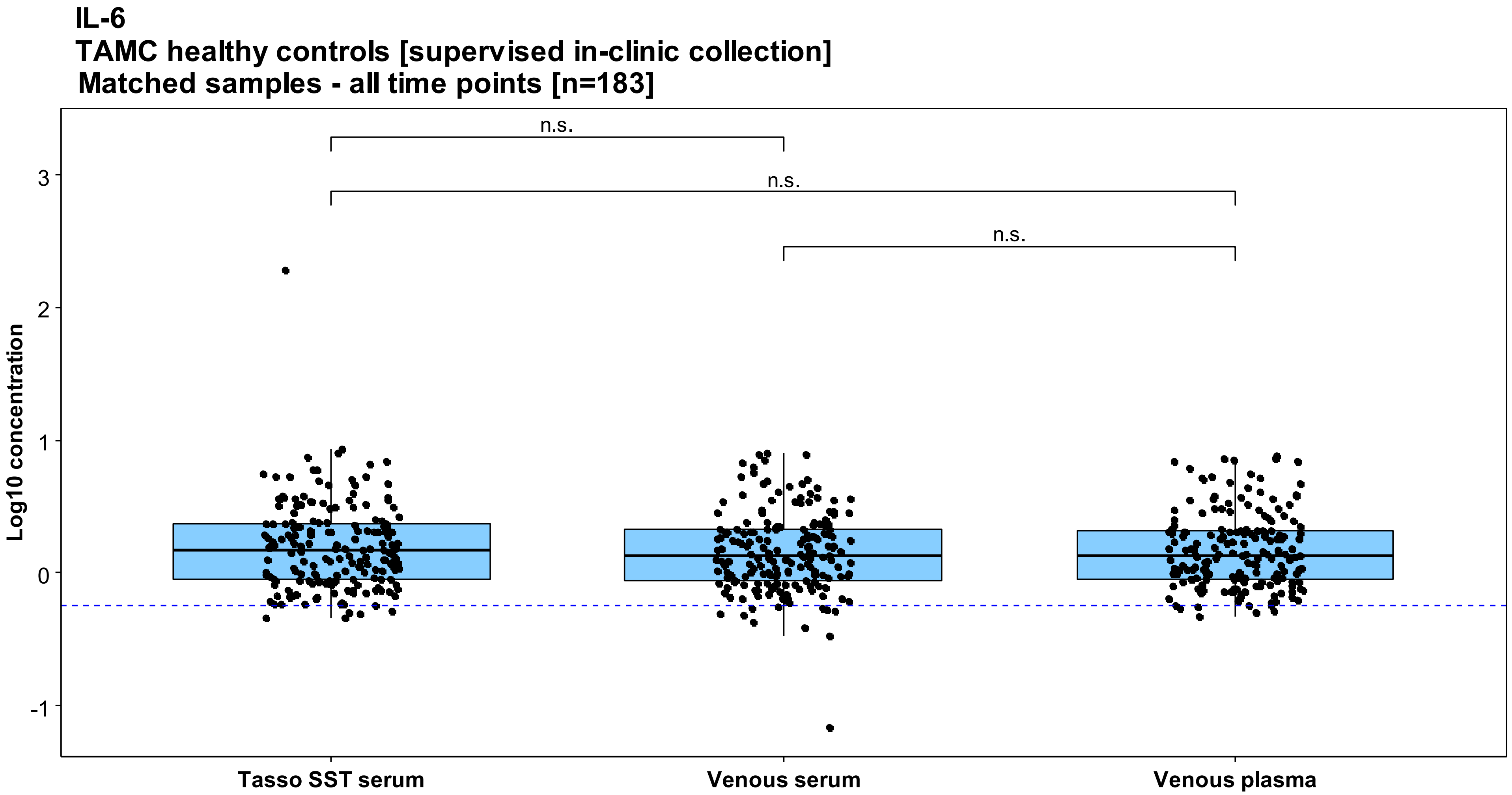

IL-6
TAMC healthy controls [supervised in-clinic collection]
Matched samples - all time points [n=183]
n.s.
n.s.
n.s.
3
2
1
0
-1
Log10 concentration
Tasso SST serum
Venous serum
Venous plasma

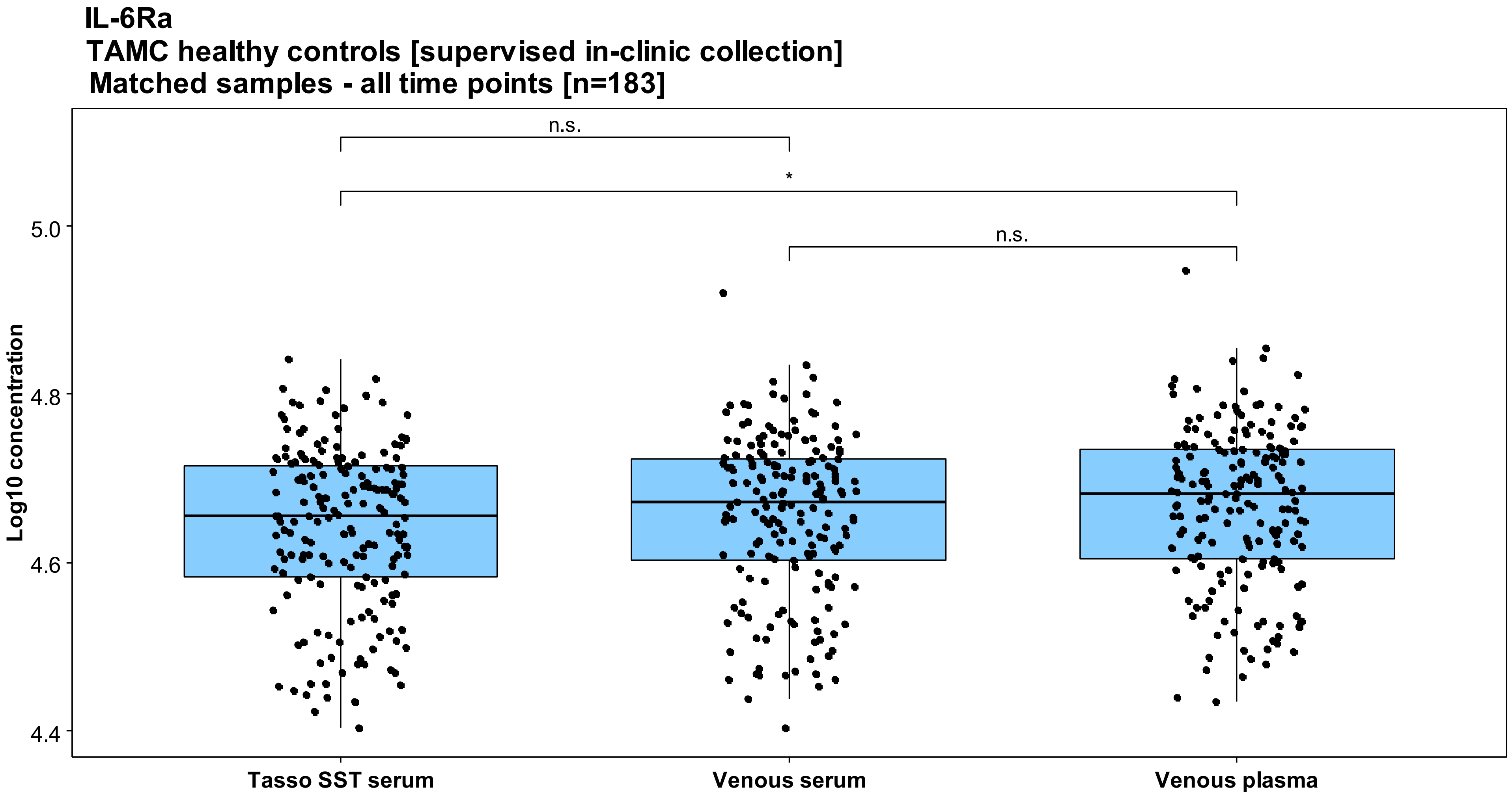
IL-6Ra
TAMC healthy controls [supervised in-clinic collection]
Matched samples - all time points [n=183]
n.s.
*
n.s.
5.0
4.8
4.6
4.4
Log10 concentration
Tasso SST serum
Venous serum
Venous plasma

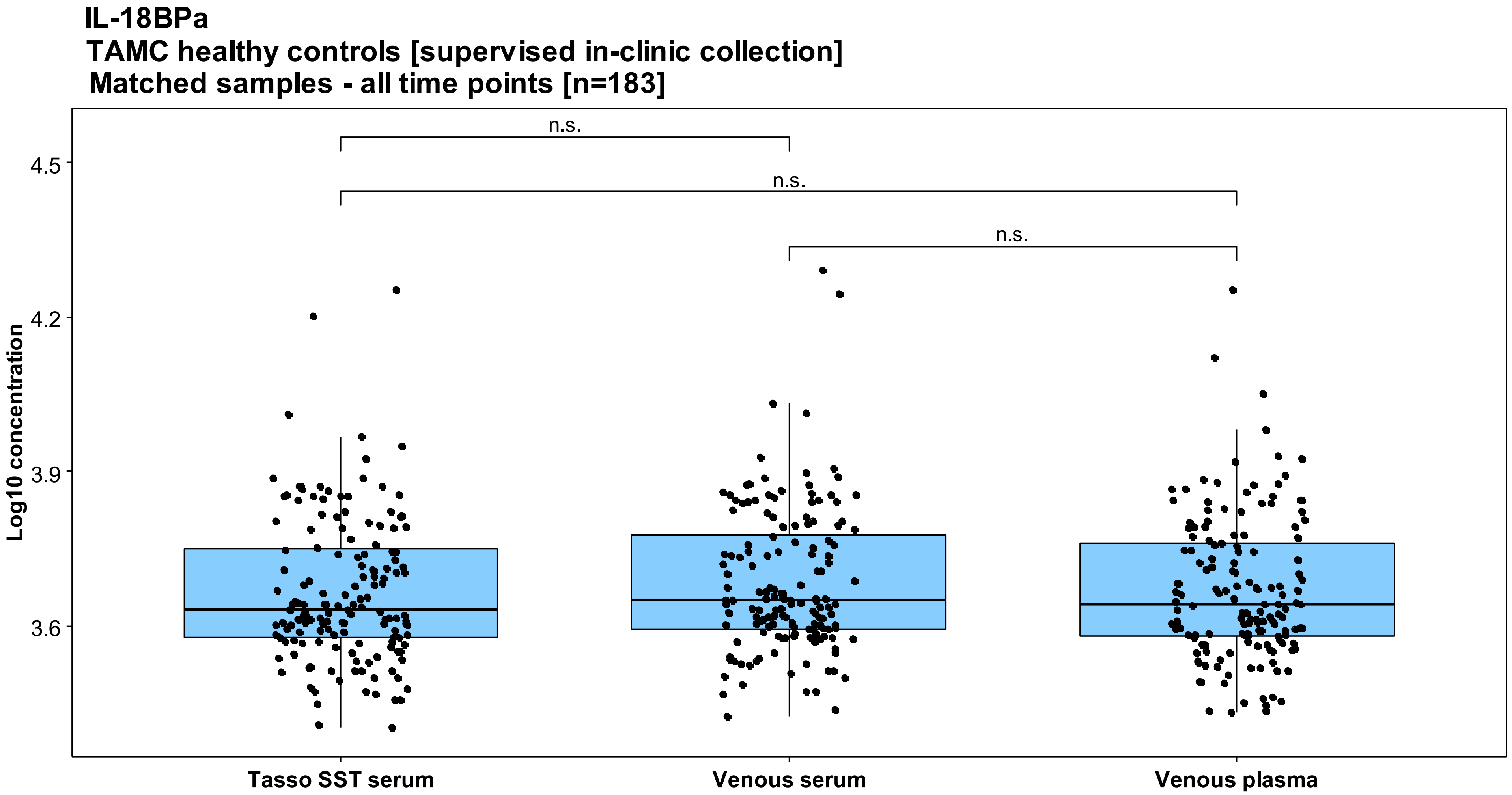

IL-18BPa
TAMC healthy controls [supervised in-clinic collection]
Matched samples - all time points [n=183]
n.s.
n.s.
n.s.
4.5
4.2
3.9
3.6
Log10 concentration
Tasso SST serum
Venous serum
Venous plasma

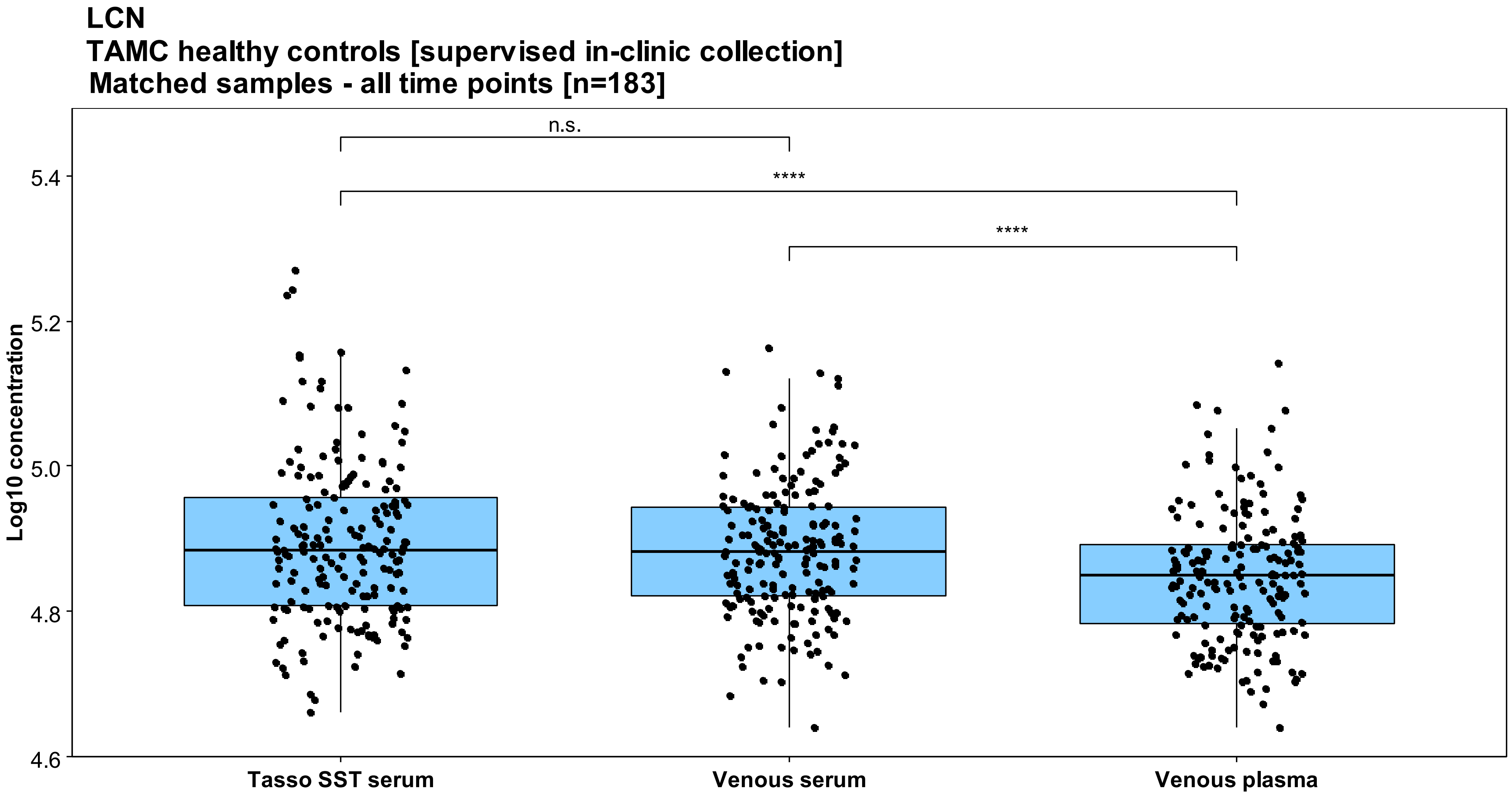
LCN
TAMC healthy controls [supervised in-clinic collection]
Matched samples - all time points [n=183]
n.s.
****
****
Log10 concentration
5.4
5.2
5.0
4.8
4.6
Tasso SST serum
Venous serum
Venous plasma

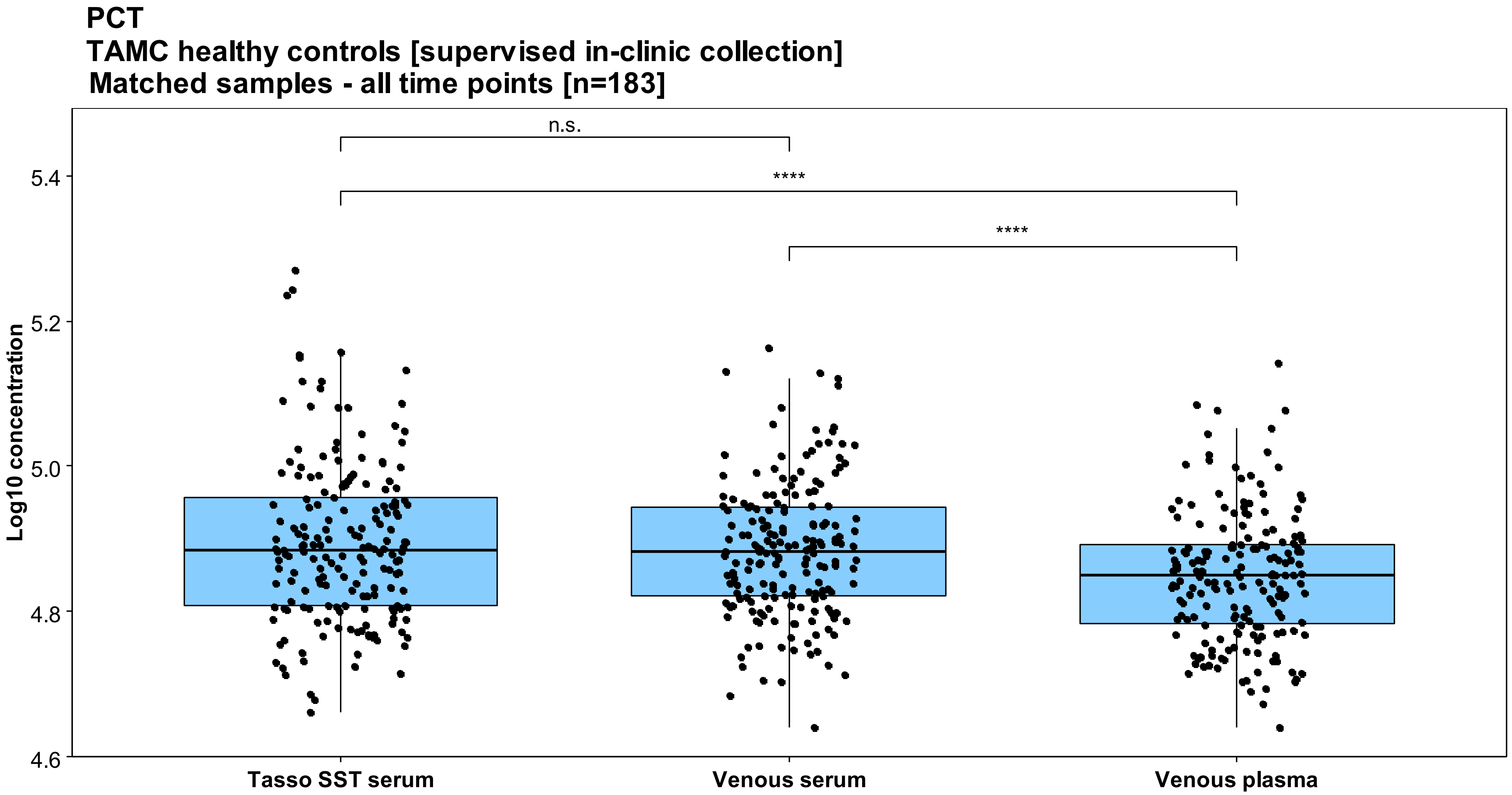

PCT
TAMC healthy controls [supervised in-clinic collection]
Matched samples - all time points [n=183]
n.s.
****
****
Log10 concentration
5.4
5.2
5.0
4.8
4.6
Tasso SST serum
Venous serum
Venous plasma

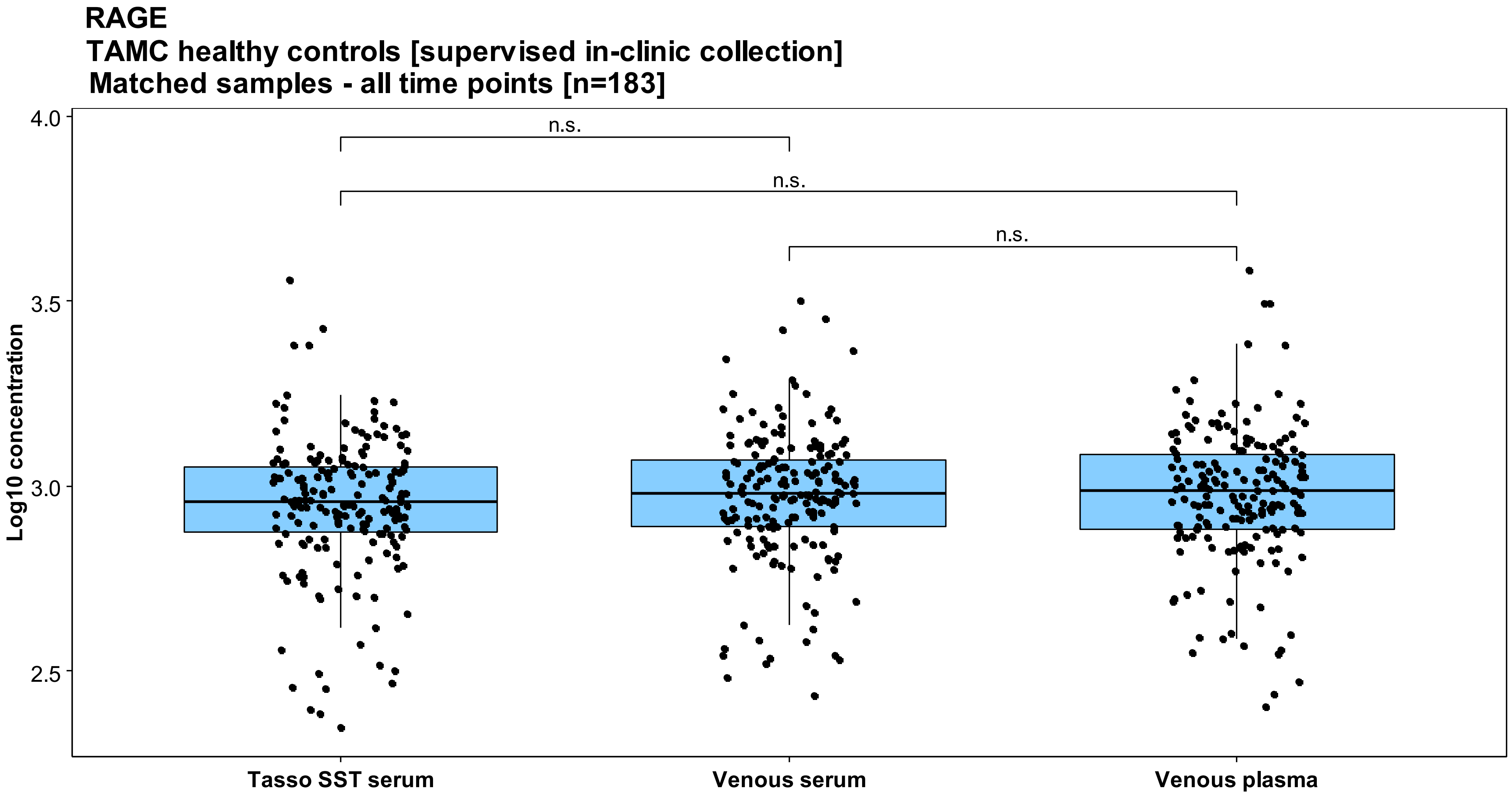

RAGE
TAMC healthy controls [supervised in-clinic collection]
Matched samples - all time points [n=183]
Log10 concentration
4.0
3.5
3.0
2.5
n.s.
n.s.
n.s.
Tasso SST serum
Venous serum
Venous plasma

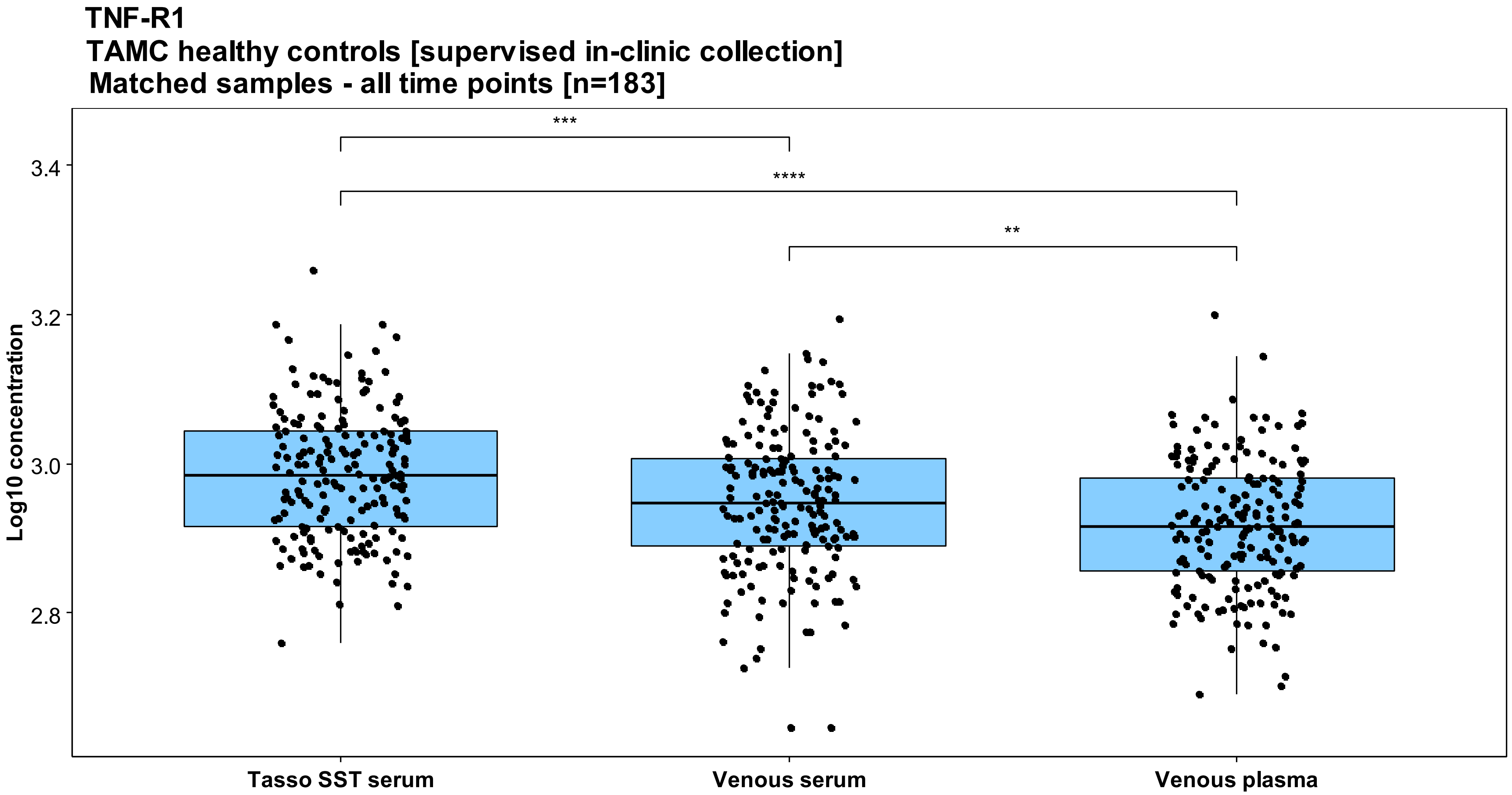
TNF-R1
TAMC healthy controls [supervised in-clinic collection]
Matched samples - all time points [n=183]
***
****
**
3.4
3.2
3.0
2.8
Log10 concentration
Tasso SST serum
Venous serum
Venous plasma

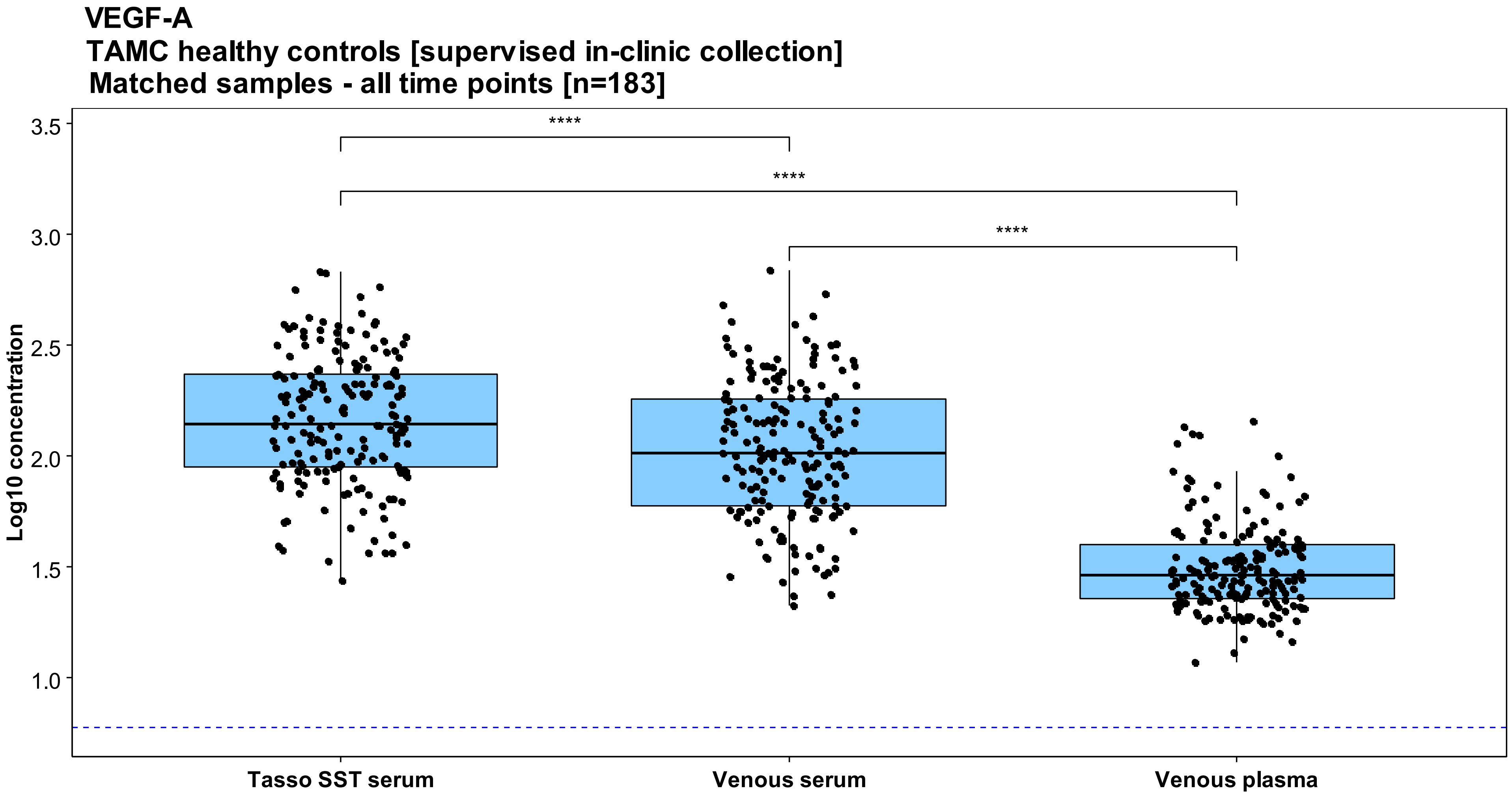
VEGF-A
TAMC healthy controls [supervised in-clinic collection]
Matched samples - all time points [n=183]
Log10 concentration
3.5
3.0
2.5
2.0
1.5
1.0
****
****
****
Tasso SST serum
Venous serum
Venous plasma
