# Supplementary material for: Assessing the use of a micro-sampling device for measuring blood protein levels in healthy subjects and COVID-19 patients
Source: PLoS One. 2022 Aug 10;17(8):e0272572. doi: 10.1371/journal.pone.0272572 (PMC9365123; doi:10.1371/journal.pone.0272572)

CD163
TAMC healthy controls [supervised in-clinic collection]
Matched Venous serum and Tasso SST serum - all time points [n=152]

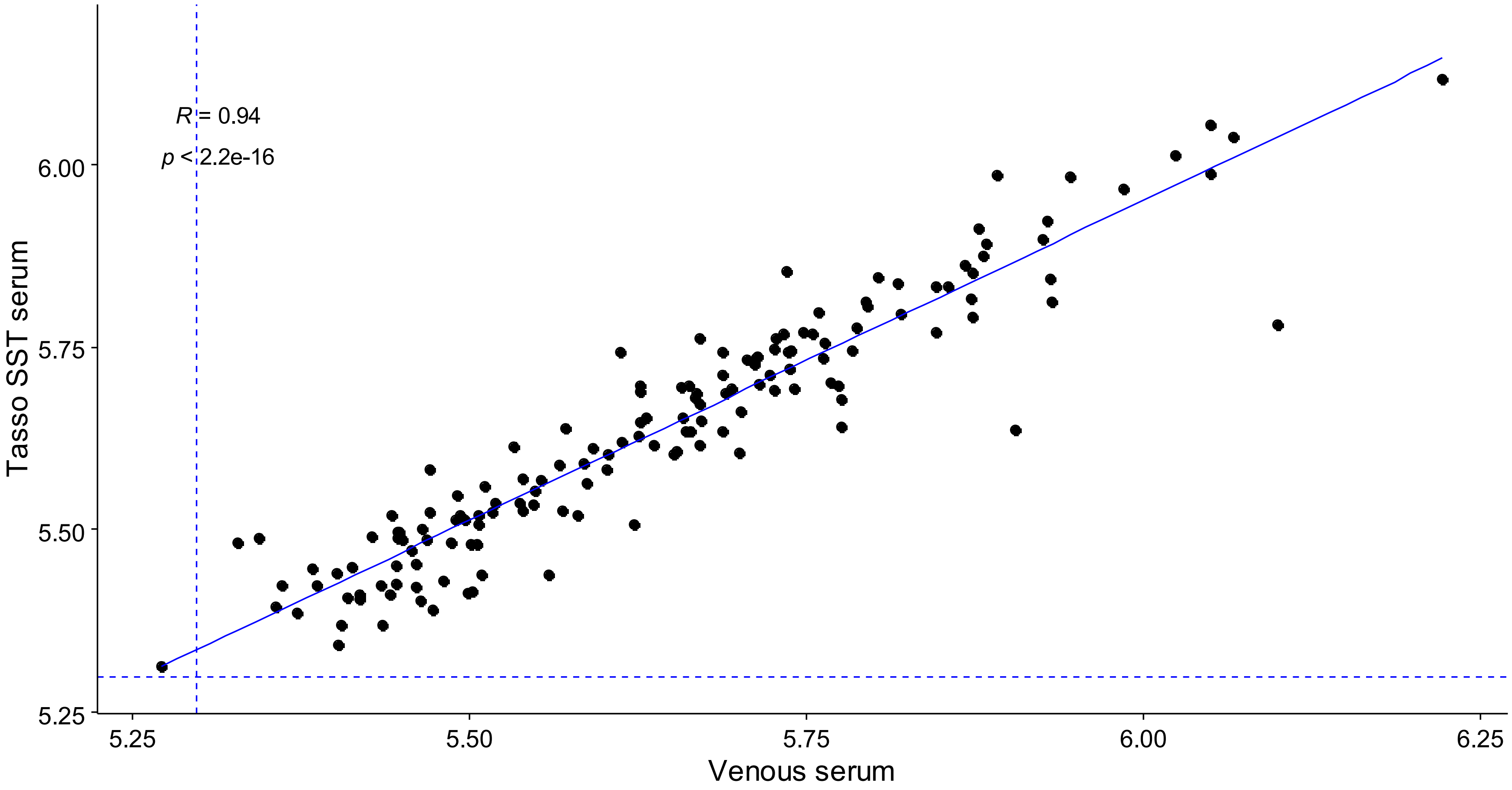

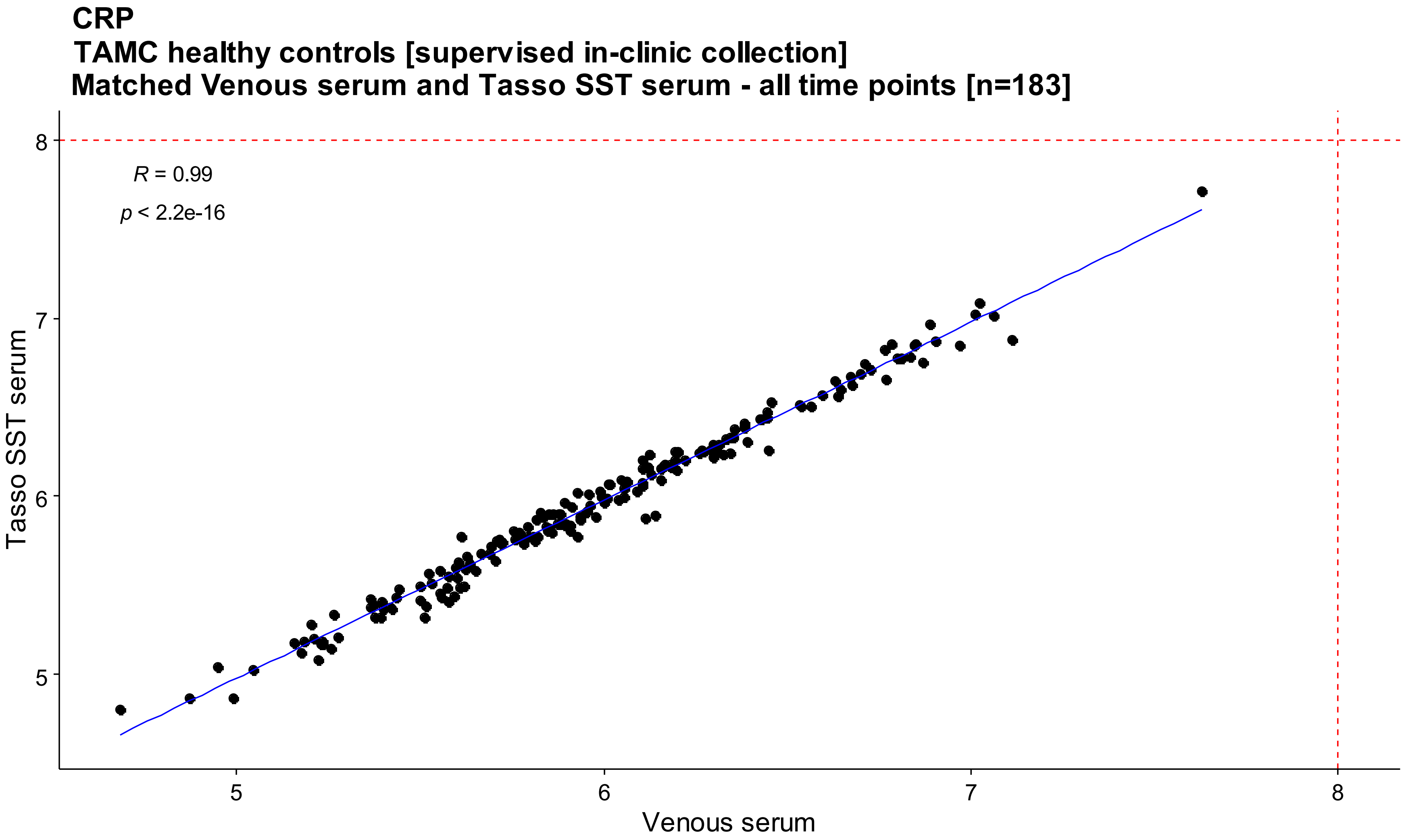
CRP
TAMC healthy controls [supervised in-clinic collection]
Matched Venous serum and Tasso SST serum - all time points [n=183]
R = 0.99
p < 2.2e-16
Tasso SST serum
Venous serum
5
6
7
8

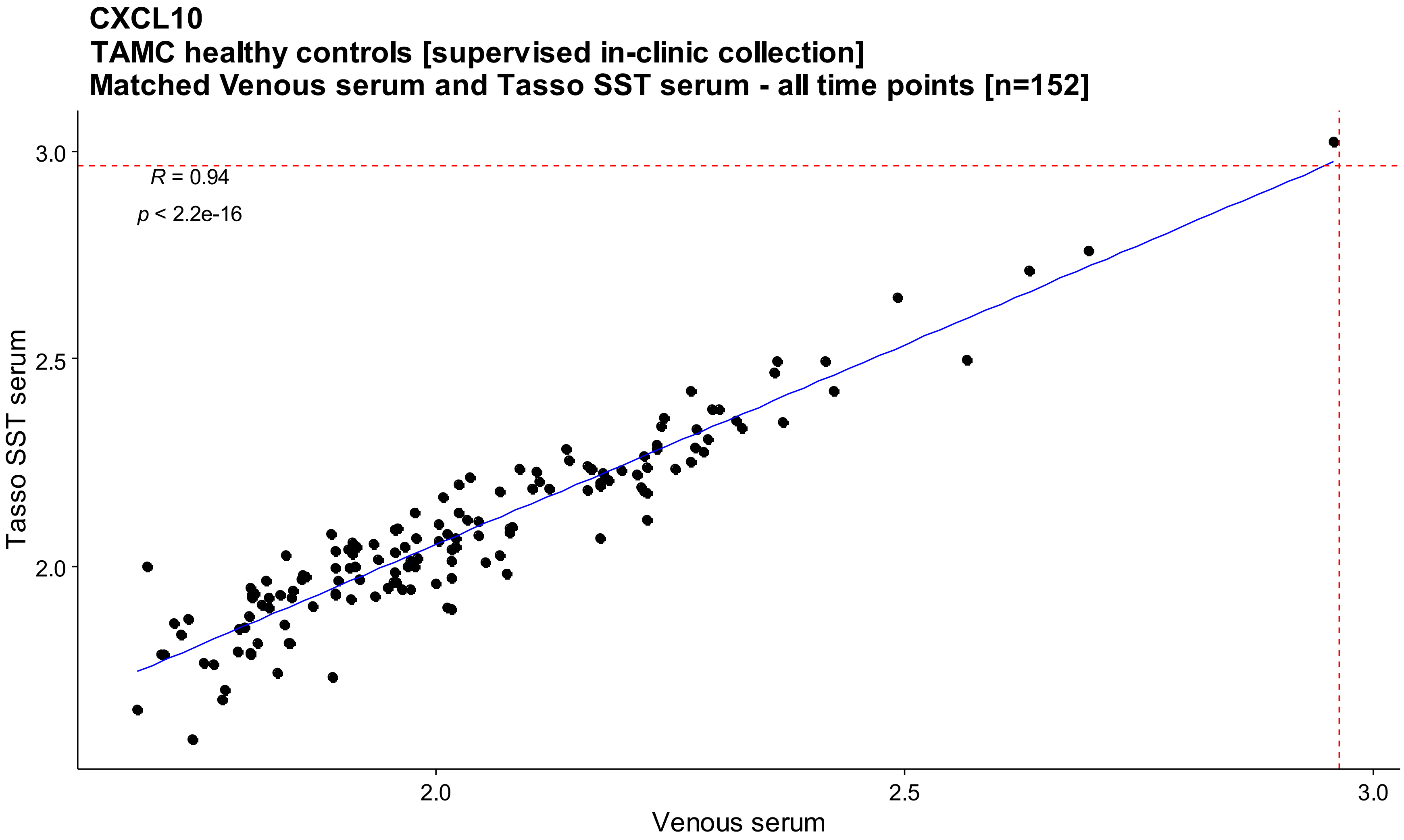
CXCL10
TAMC healthy controls [supervised in-clinic collection]
Matched Venous serum and Tasso SST serum - all time points [n=152]
R = 0.94
p < 2.2e-16
Tasso SST serum
3.0
2.5
2.0
Venous serum
2.0
2.5
3.0

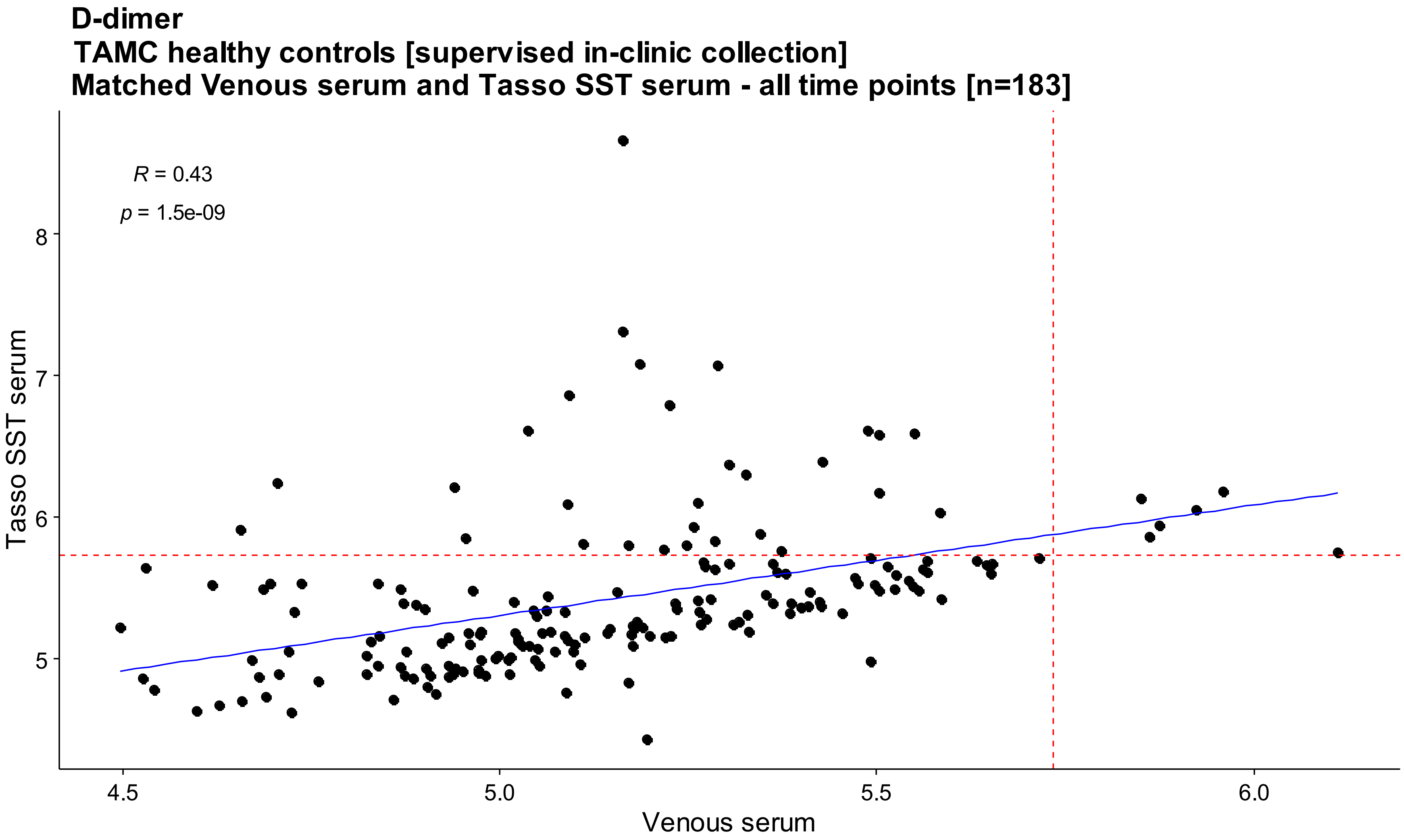

D-dimer
TAMC healthy controls [supervised in-clinic collection]
Matched Venous serum and Tasso SST serum - all time points [n=183]
R = 0.43
p = 1.5e-09
Tasso SST serum
Venous serum
8
7
6
5
4.5
5.0
5.5
6.0

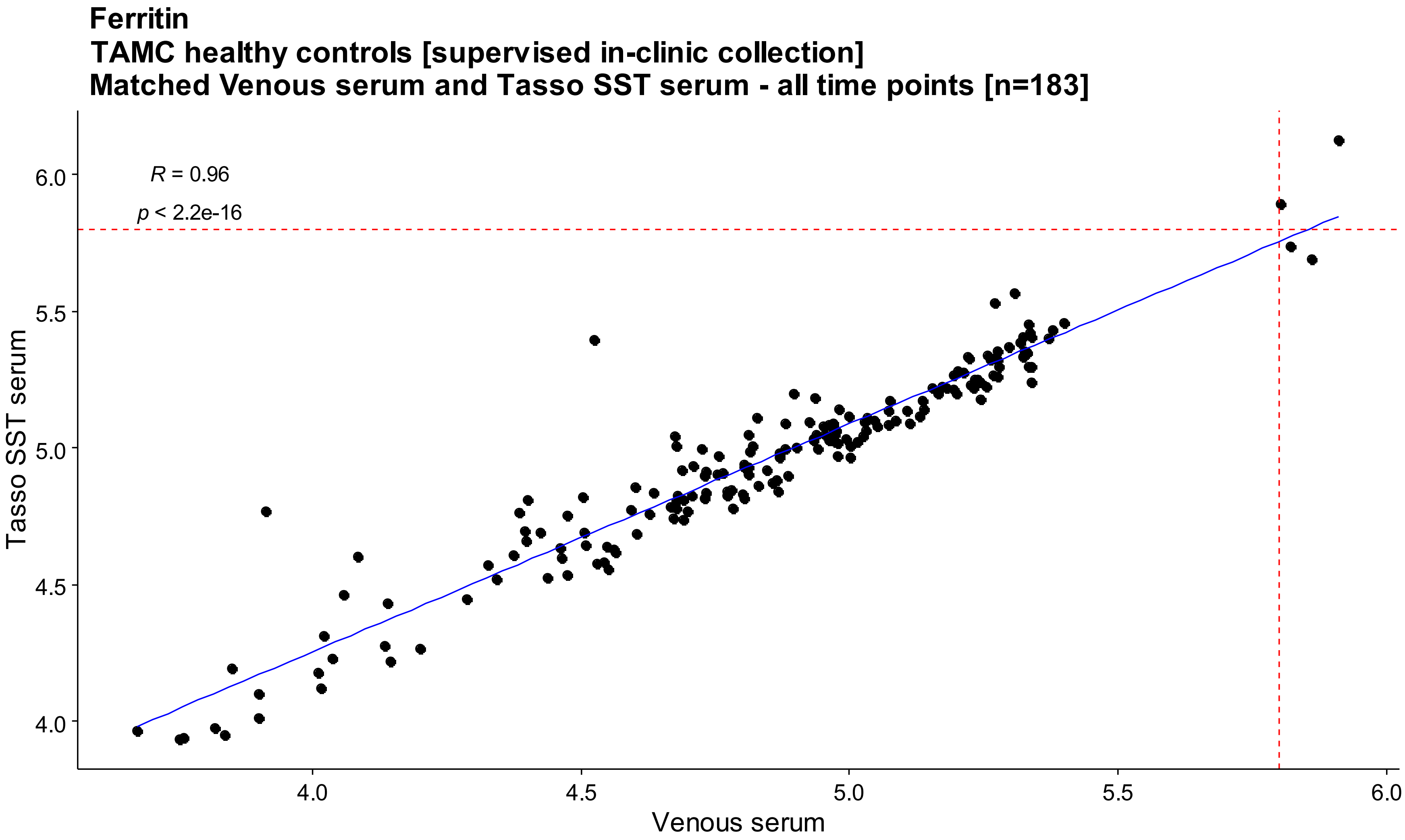
Ferritin
TAMC healthy controls [supervised in-clinic collection]
Matched Venous serum and Tasso SST serum - all time points [n=183]
R = 0.96
p < 2.2e-16
Tasso SST serum
Venous serum
6.0
5.5
5.0
4.5
4.0
4.0
4.5
5.0
5.5
6.0

ICAM-1
TAMC healthy controls [supervised in-clinic collection]
Matched Venous serum and Tasso SST serum - all time points [n=183]

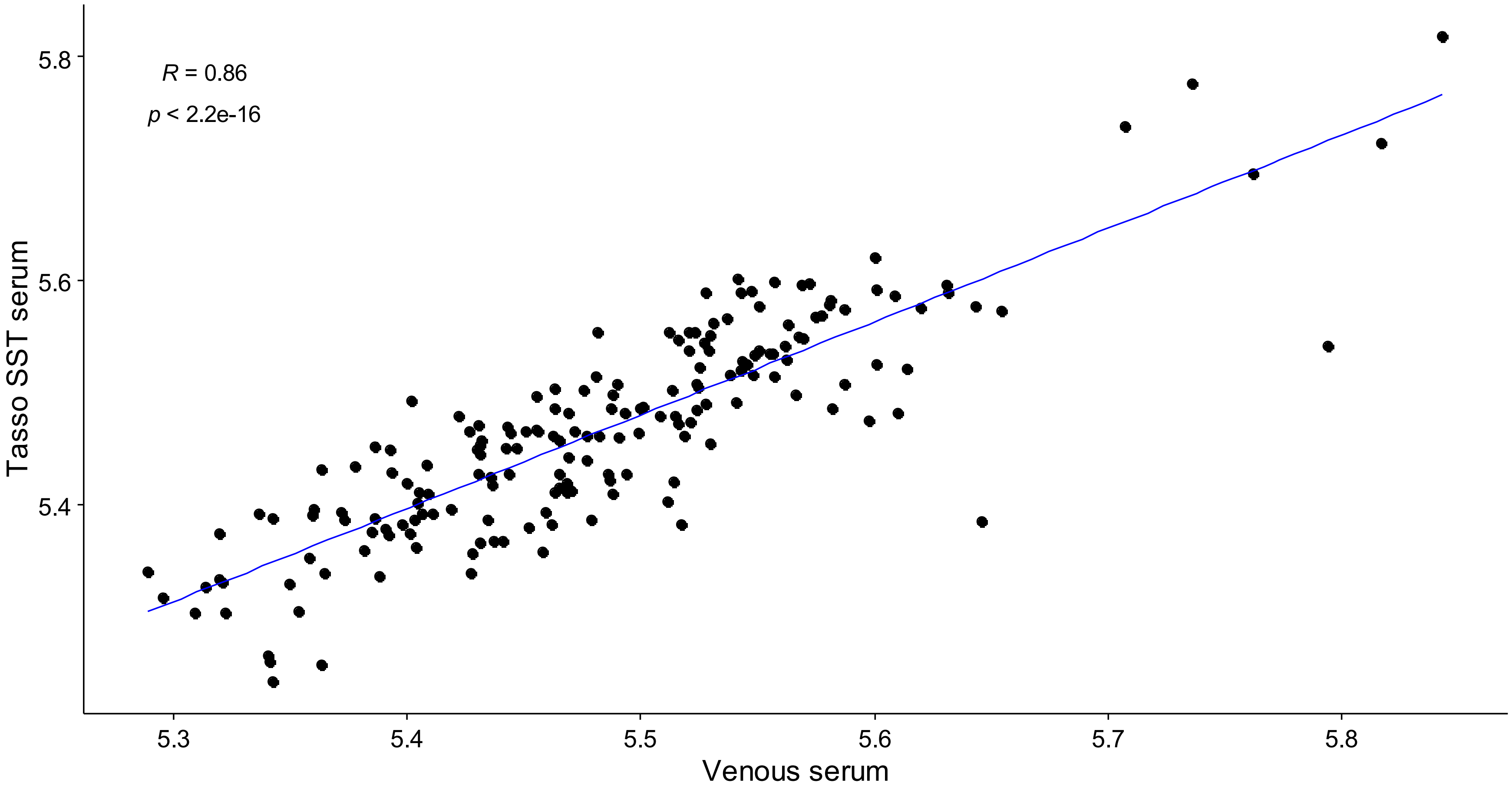

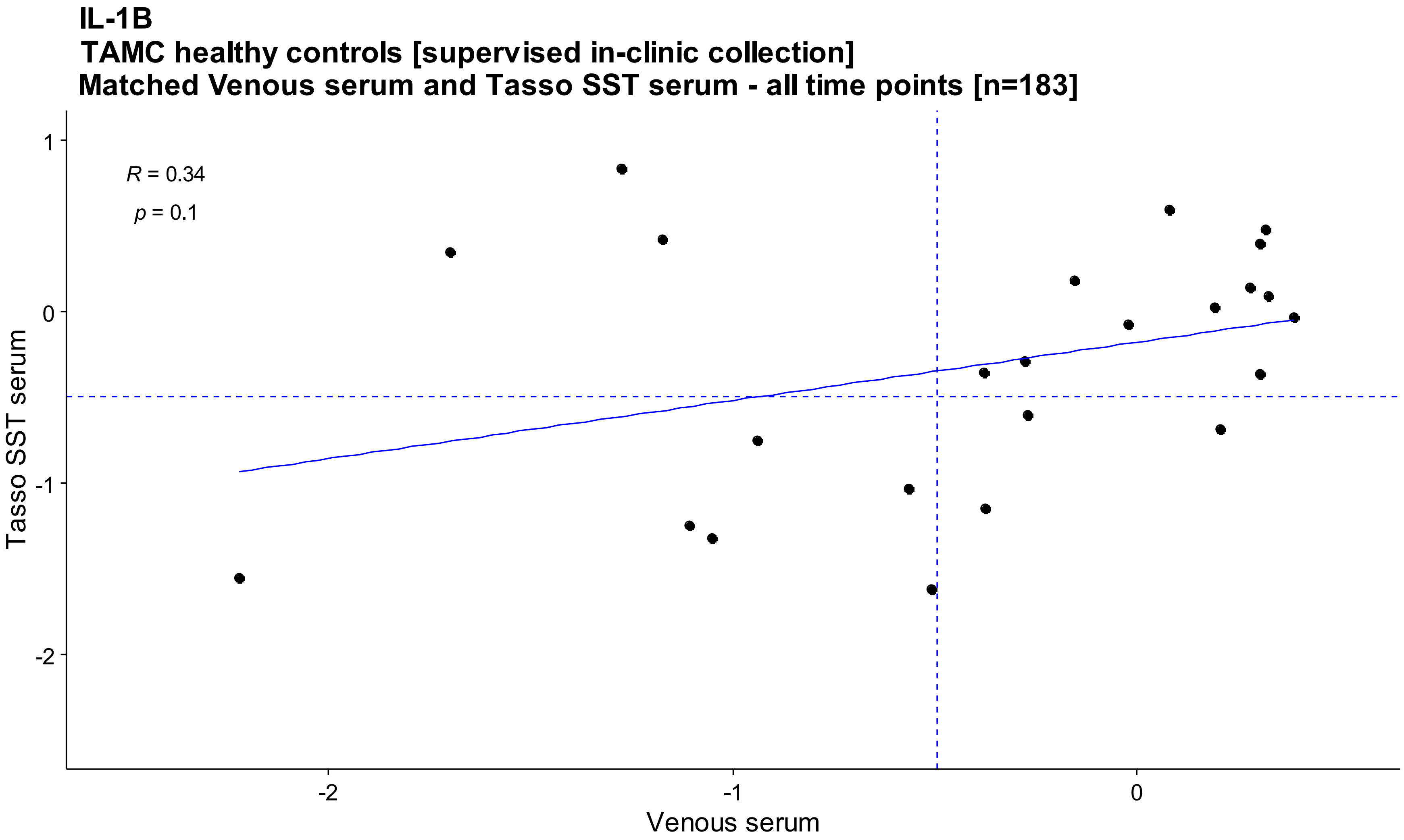
IL-1B
TAMC healthy controls [supervised in-clinic collection]
Matched Venous serum and Tasso SST serum - all time points [n=183]
R = 0.34
p = 0.1
Tasso SST serum
1
0
-1
-2
-2
-1
0
Venous serum

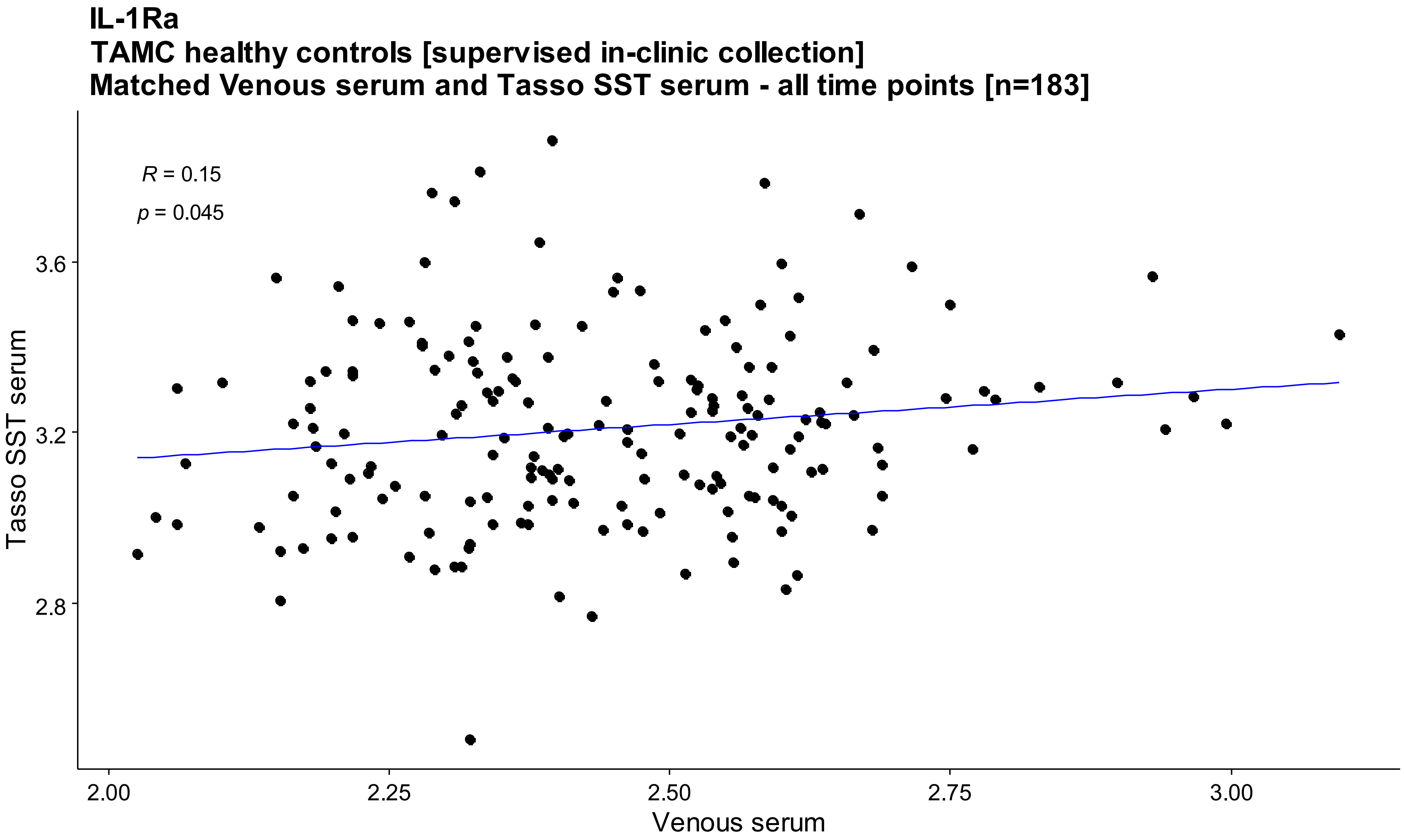
IL-1Ra
TAMC healthy controls [supervised in-clinic collection]
Matched Venous serum and Tasso SST serum - all time points [n=183]
R = 0.15
p = 0.045
3.6
3.2
2.8
Tasso SST serum
2.00
2.25
2.50
2.75
3.00
Venous serum

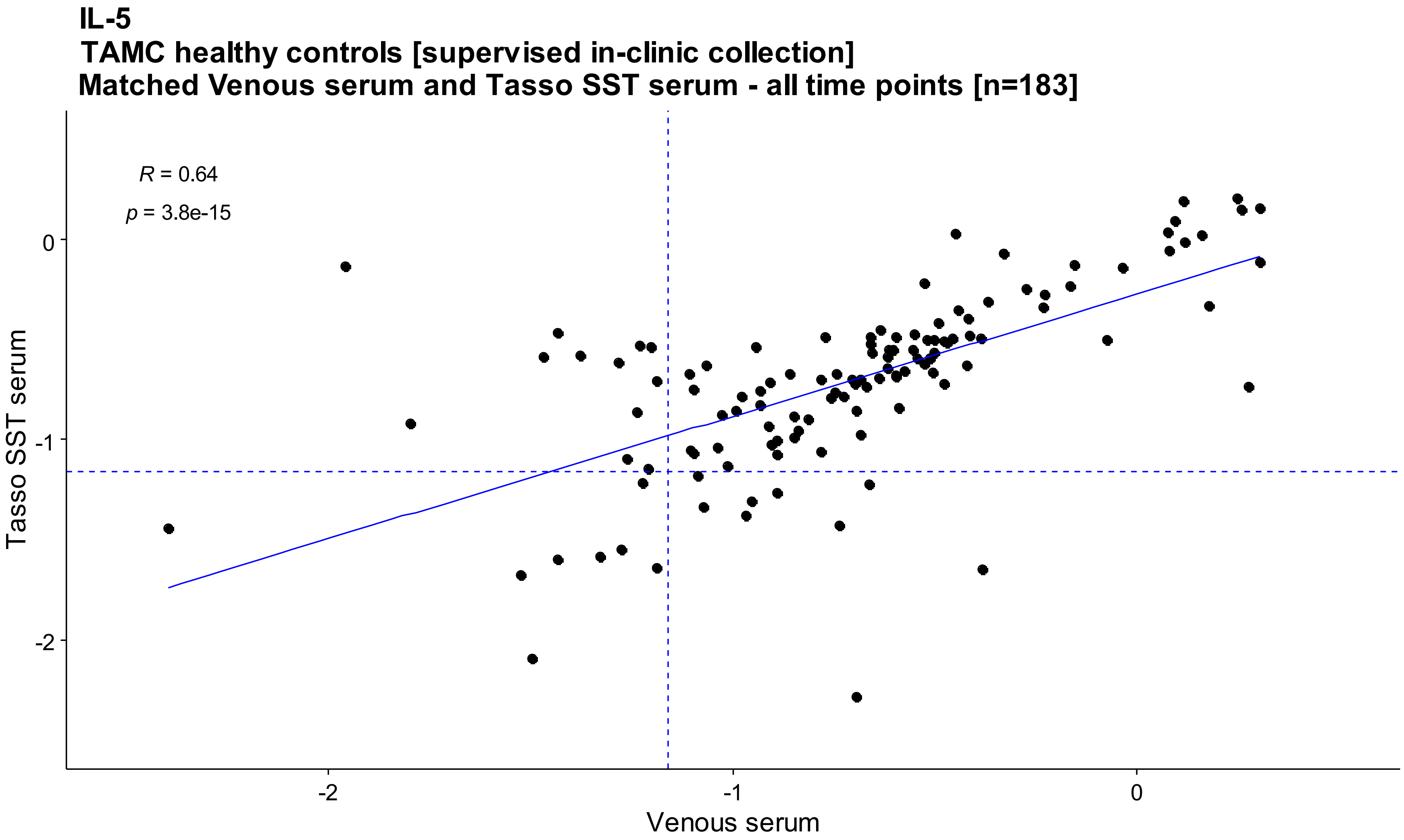
IL-5
TAMC healthy controls [supervised in-clinic collection]
Matched Venous serum and Tasso SST serum - all time points [n=183]
R = 0.64
p = 3.8e-15
0
-1
-2
Tasso SST serum
-2
-1
0
Venous serum

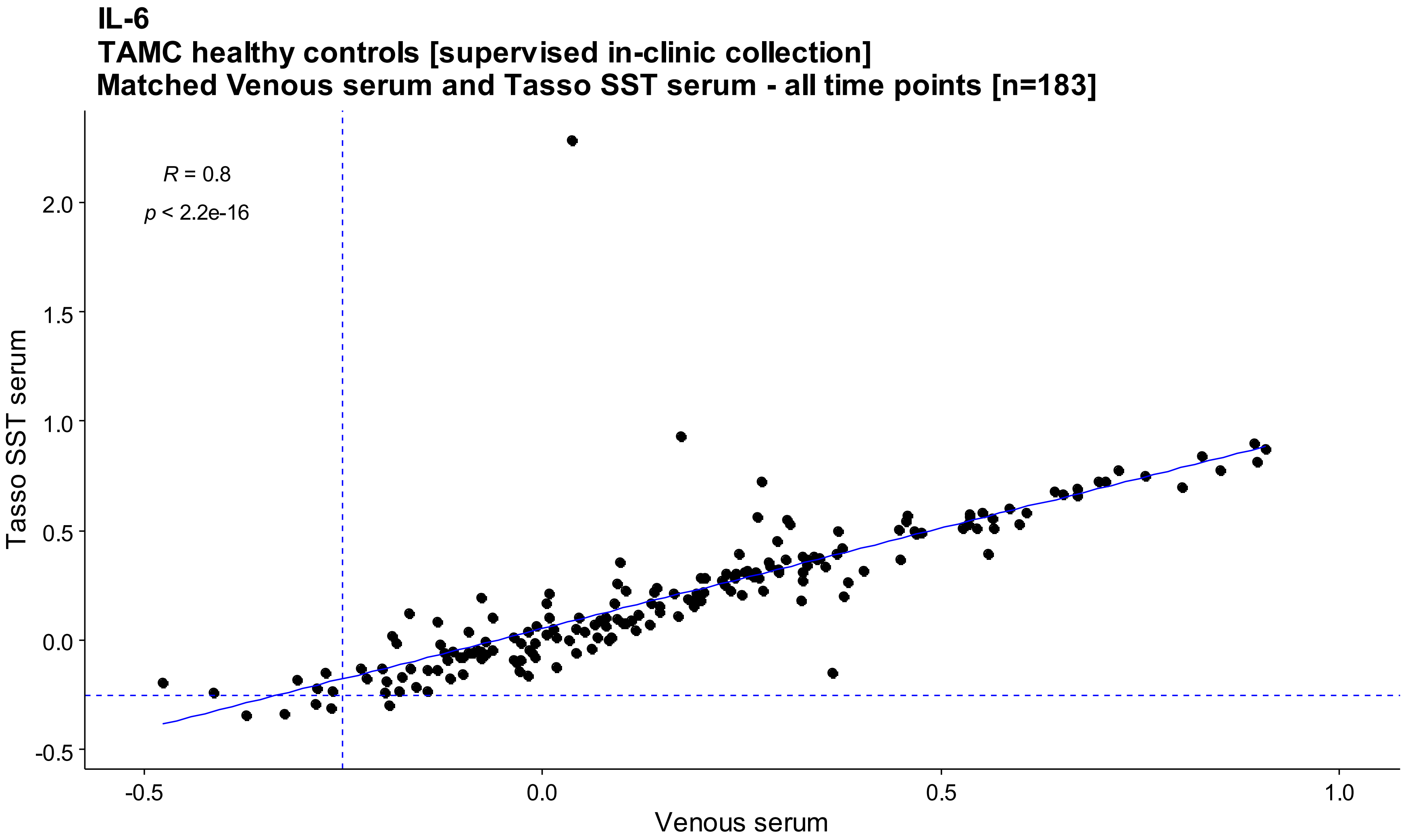

IL-6
TAMC healthy controls [supervised in-clinic collection]
Matched Venous serum and Tasso SST serum - all time points [n=183]
R = 0.8
p < 2.2e-16
Tasso SST serum
Venous serum
2.0
1.5
1.0
0.5
0.0
-0.5
-0.5
0.0
0.5
1.0

IL-6Ra
TAMC healthy controls [supervised in-clinic collection]
Matched Venous serum and Tasso SST serum - all time points [n=183]

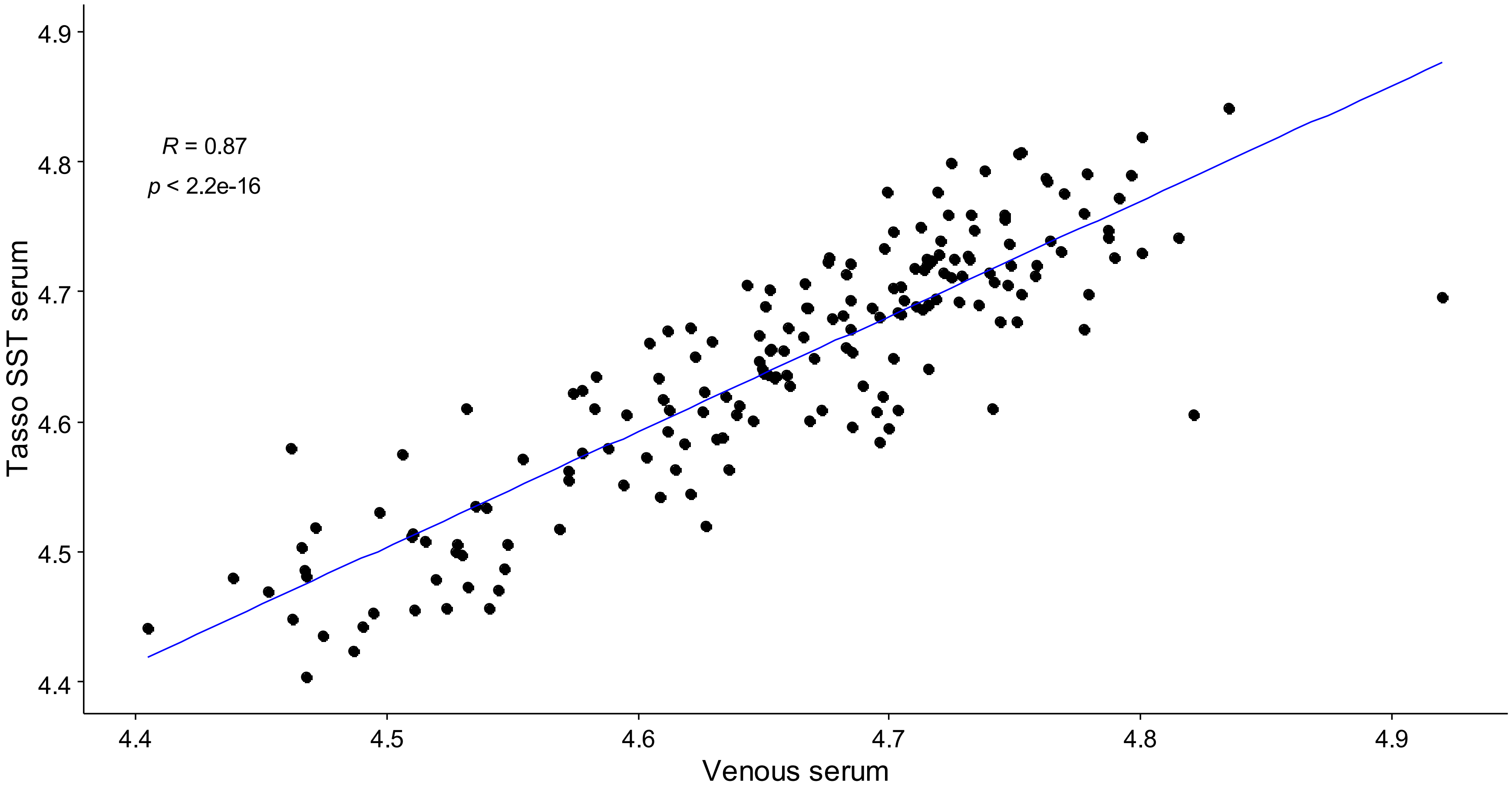

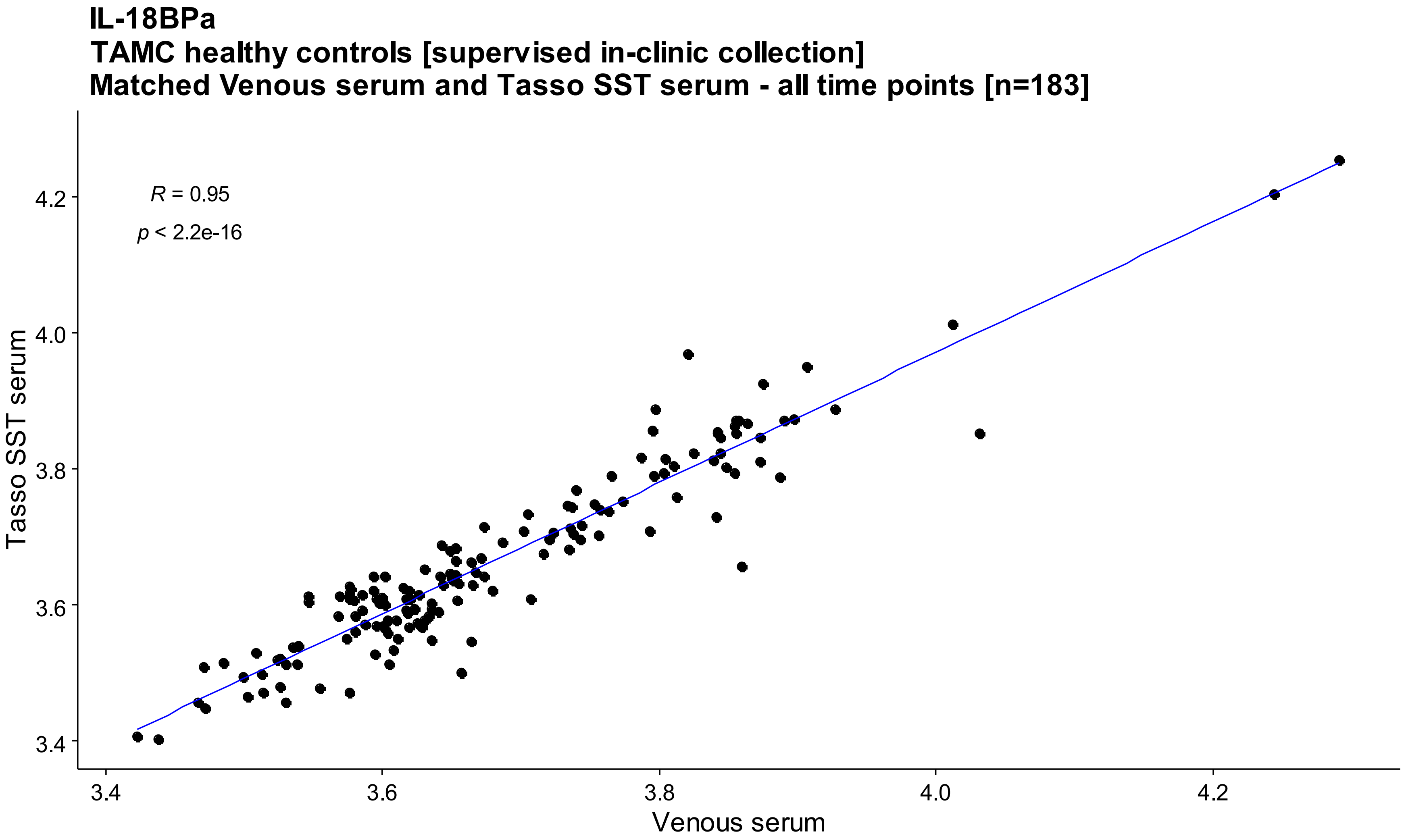
IL-18BPa
TAMC healthy controls [supervised in-clinic collection]
Matched Venous serum and Tasso SST serum - all time points [n=183]
R = 0.95
p < 2.2e-16
Tasso SST serum
4.2
4.0
3.8
3.6
3.4
3.4
3.6
3.8
4.0
4.2
Venous serum

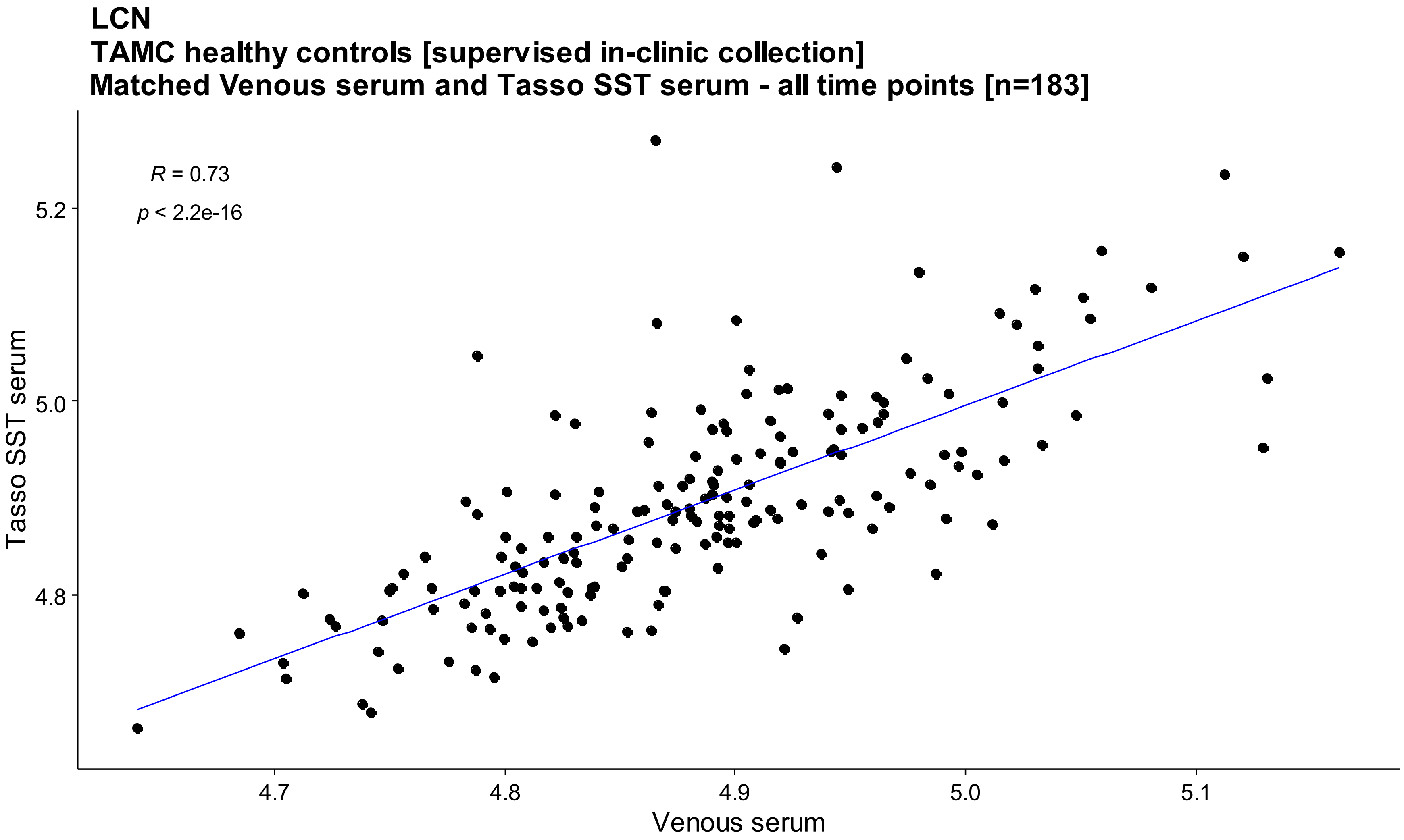
LCN
TAMC healthy controls [supervised in-clinic collection]
Matched Venous serum and Tasso SST serum - all time points [n=183]
R = 0.73
p < 2.2e-16
Tasso SST serum
Venous serum
4.8
5.0
5.2
4.7
4.8
4.9
5.0
5.1

PCT
TAMC healthy controls [supervised in-clinic collection]
Matched Venous serum and Tasso SST serum - all time points [n=183]

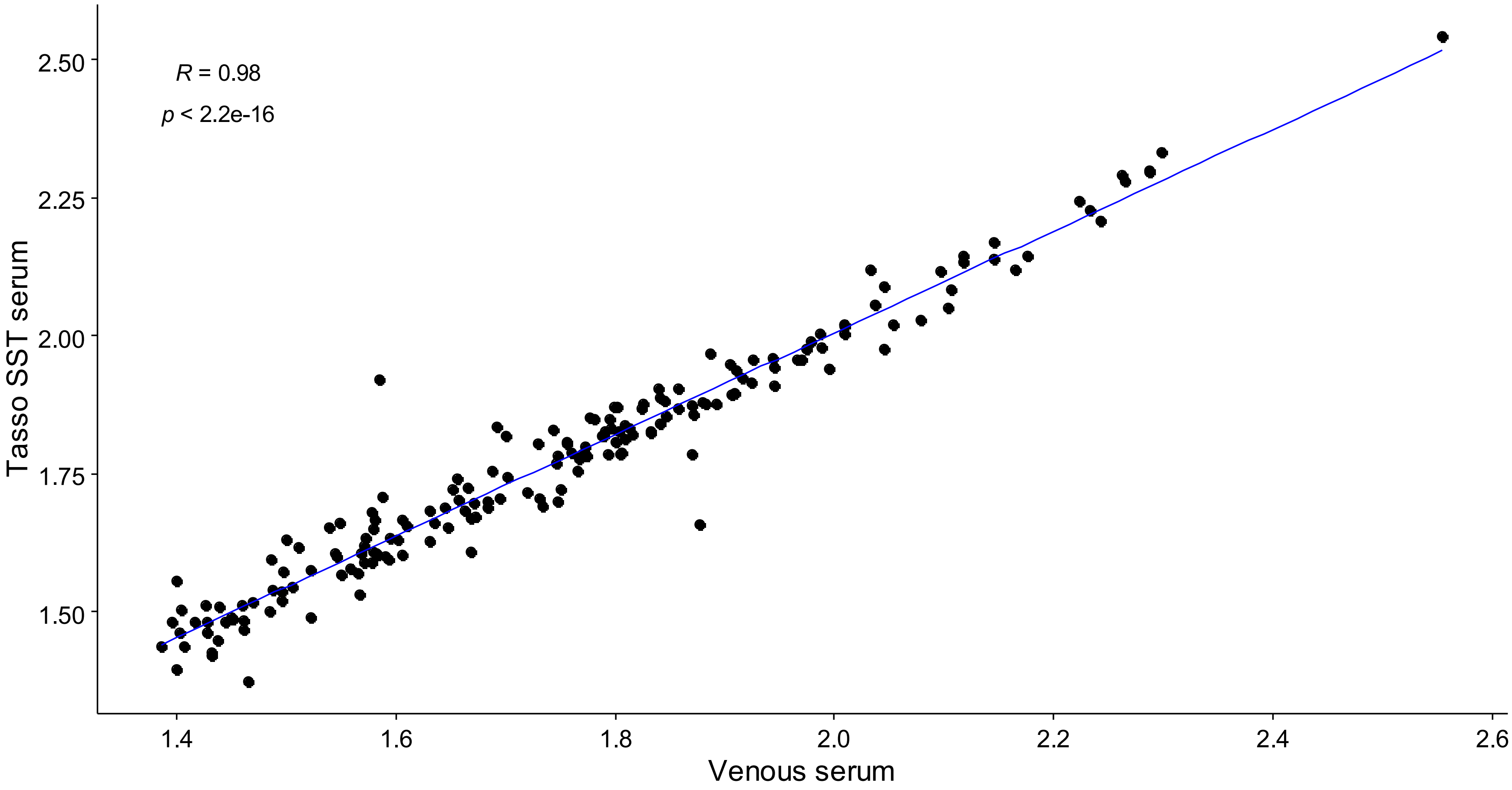

RAGE
TAMC healthy controls [supervised in-clinic collection]
Matched Venous serum and Tasso SST serum - all time points [n=183]

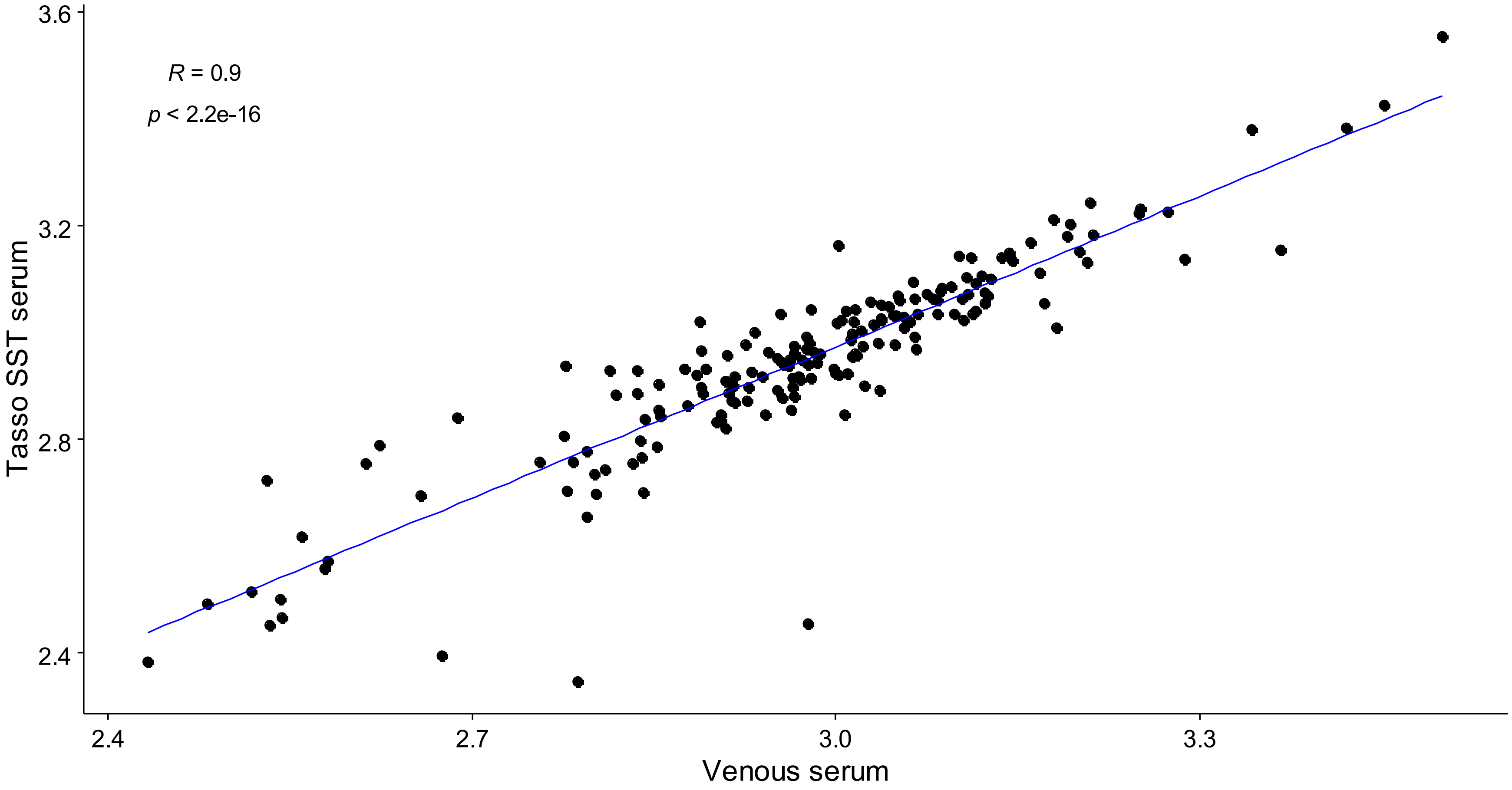

TNF-R1
TAMC healthy controls [supervised in-clinic collection]
Matched Venous serum and Tasso SST serum - all time points [n=183]

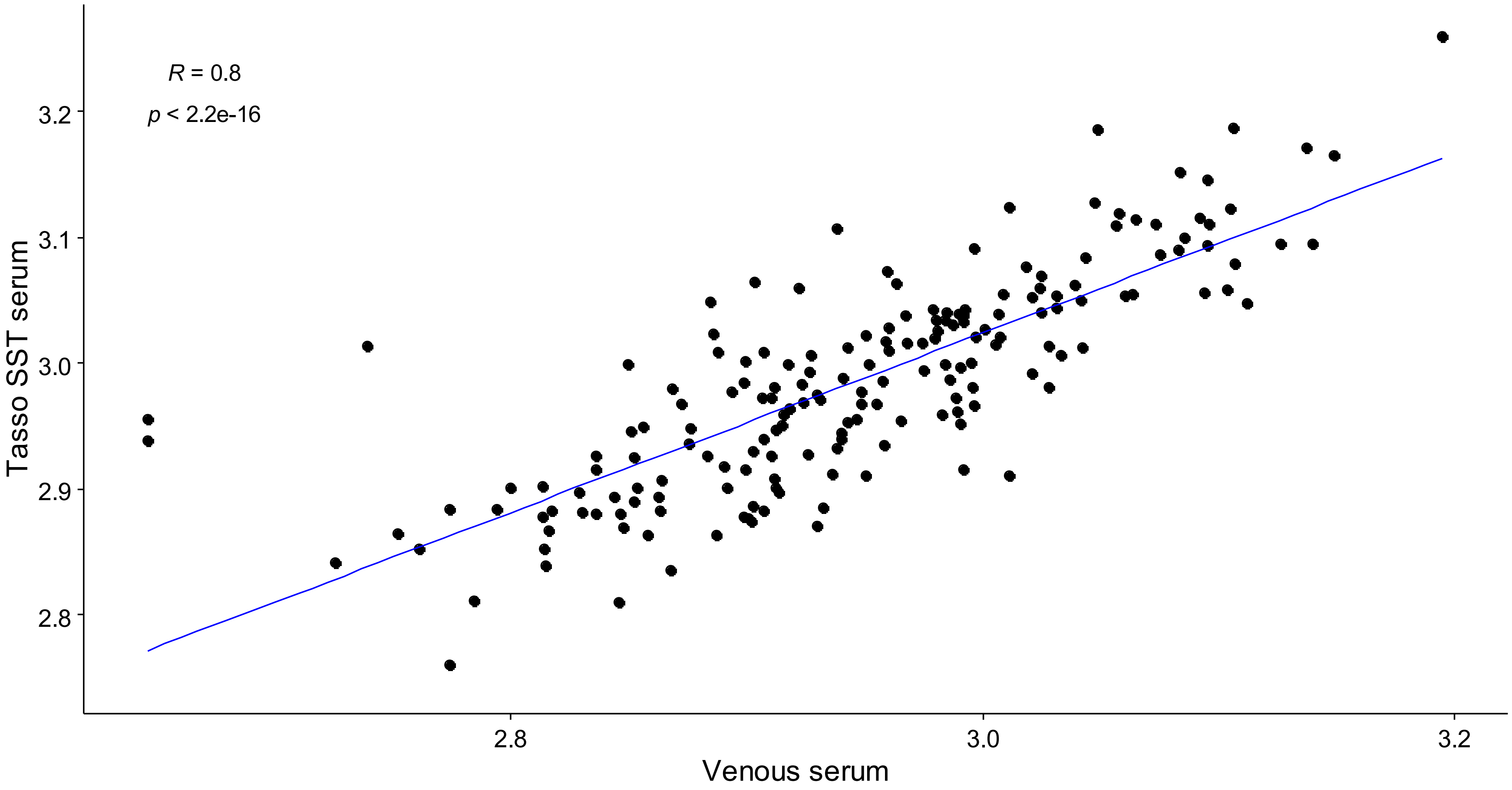

VEGF-A
TAMC healthy controls [supervised in-clinic collection]
Matched Venous serum and Tasso SST serum - all time points [n=183]

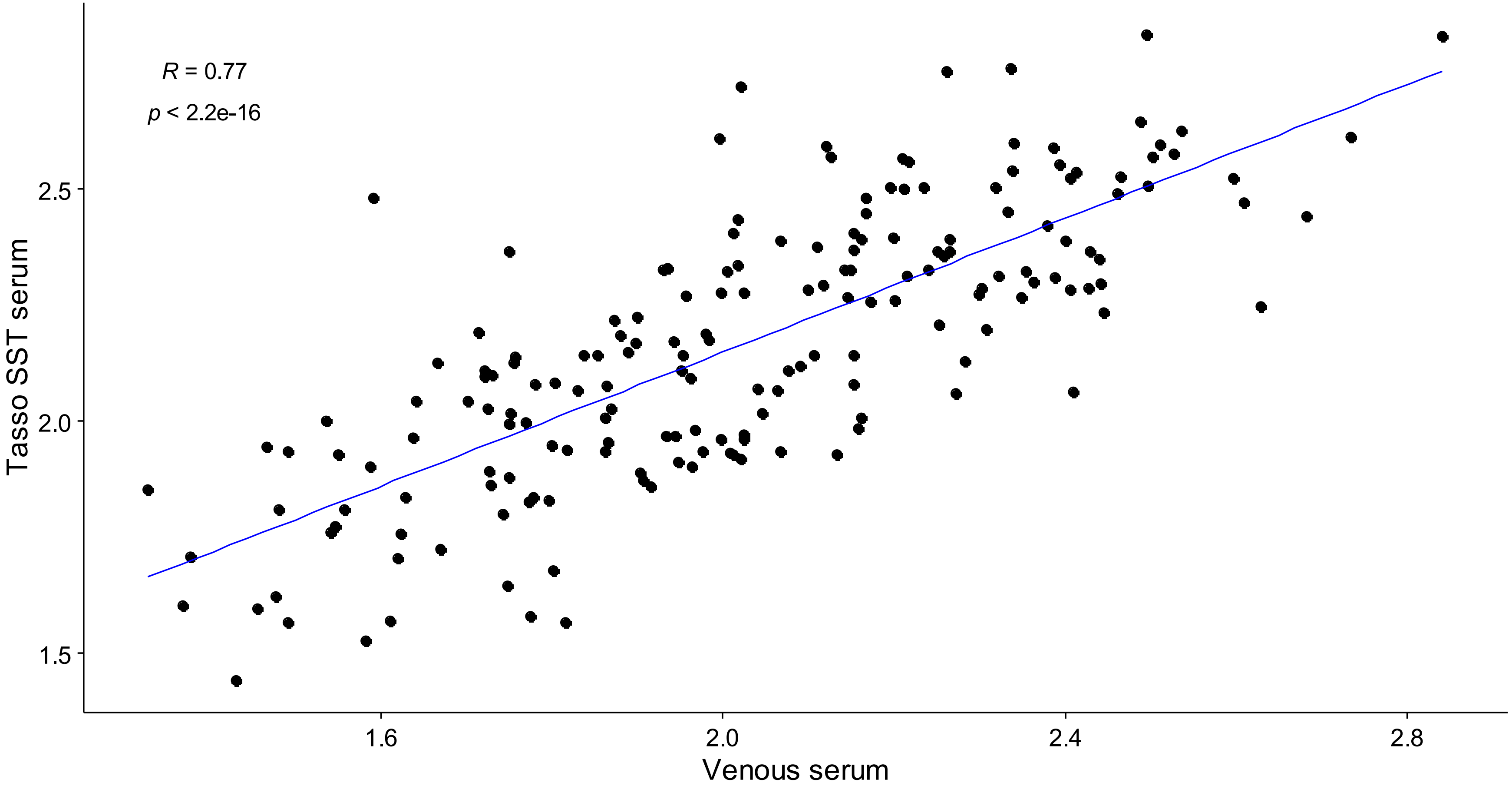

Supplement: S2 Fig — Correlation of protein concentrations in matched peripheral blood samples of 42 TAMC healthy controls obtained in-clinic using the Tasso SST (capillary serum) and phlebotomy (venous serum). Up to 5 samples were collected from each participant over a 28-day period and are aggregated here. Concentrations were log10 transformed. Scatter plots were fitted with a simple linear regression (blue line), and the Pearson correlation R and p values are shown. (PDF) [file pone.0272572.s005.pdf]
