# Supplementary material for: Assessing the use of a micro-sampling device for measuring blood protein levels in healthy subjects and COVID-19 patients
Source: PLoS One. 2022 Aug 10;17(8):e0272572. doi: 10.1371/journal.pone.0272572 (PMC9365123; doi:10.1371/journal.pone.0272572)
Supplement: S3 Fig — Bland-Altman plots of protein concentrations in matched peripheral blood samples of 42 TAMC healthy controls obtained in-clinic using the Tasso SST (capillary serum) and phlebotomy (venous serum). Up to 5 samples were collected from each participant over a 28-day period and are aggregated here. Concentrations were log10 transformed. Fixed bias in the plots was assessed by paired t-test. (PDF) [file pone.0272572.s006.pdf]

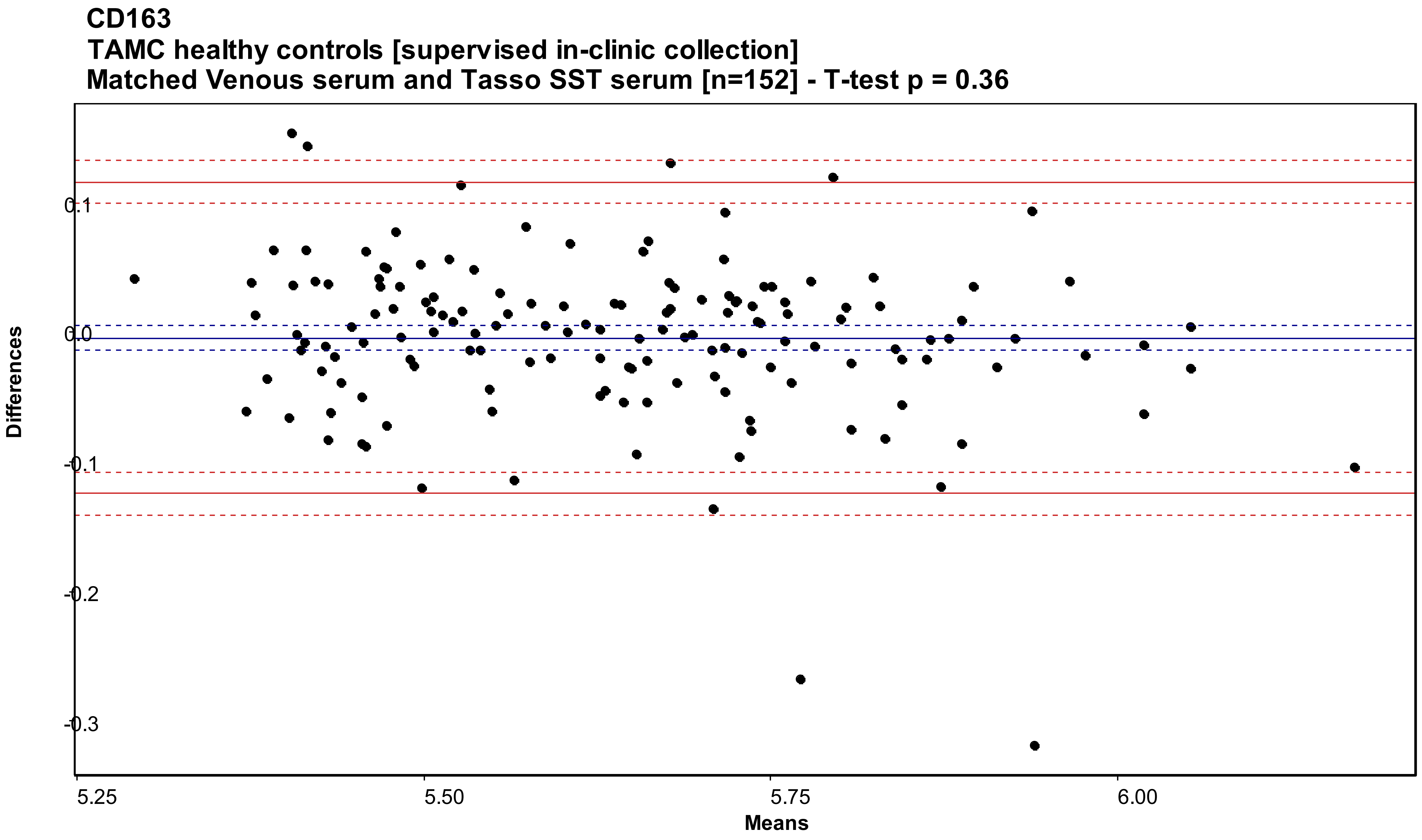

CD163
TAMC healthy controls [supervised in-clinic collection]
Matched Venous serum and Tasso SST serum [n=152] - T-test p = 0.36
Differences
0.1
0.0
-0.1
-0.2
-0.3
5.25
5.50
5.75
6.00
Means

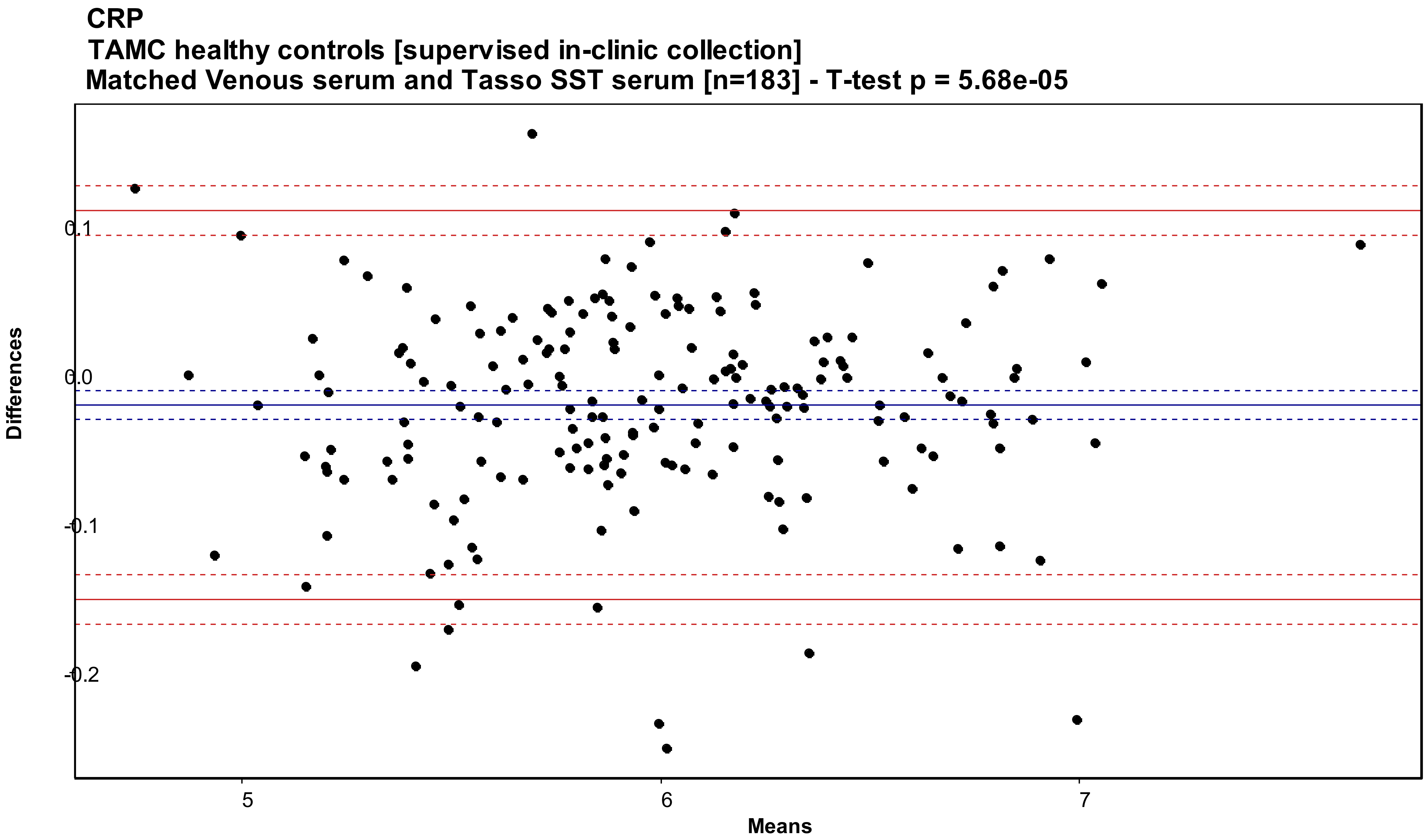

CRP
TAMC healthy controls [supervised in-clinic collection]
Matched Venous serum and Tasso SST serum [n=183] - T-test p = 5.68e-05
Differences
0.1
0.0
-0.1
-0.2
5
6
7
Means

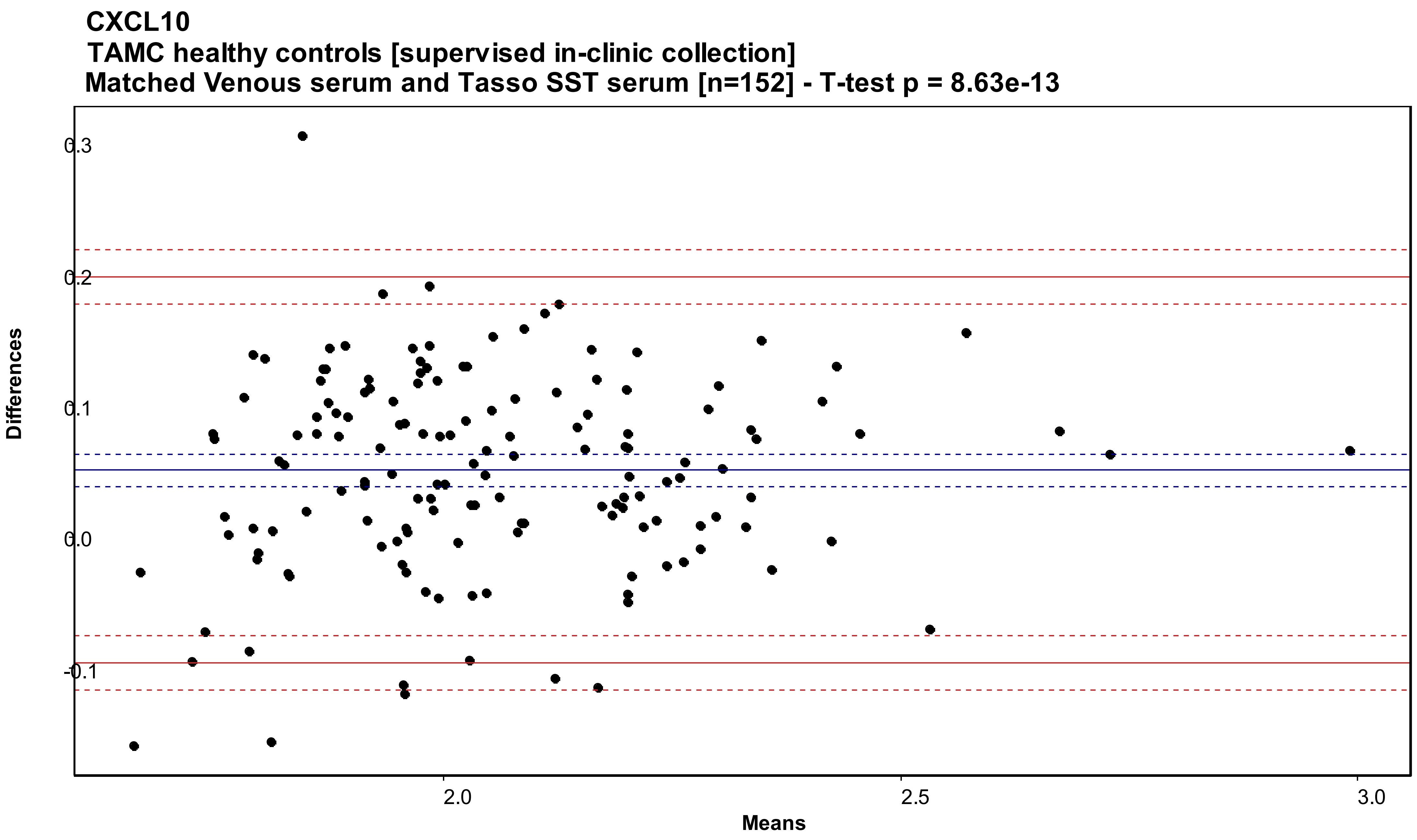
CXCL10
TAMC healthy controls [supervised in-clinic collection]
Matched Venous serum and Tasso SST serum [n=152] - T-test p = 8.63e-13
Differences
0.3
0.2
0.1
0.0
-0.1
2.0
2.5
3.0
Means

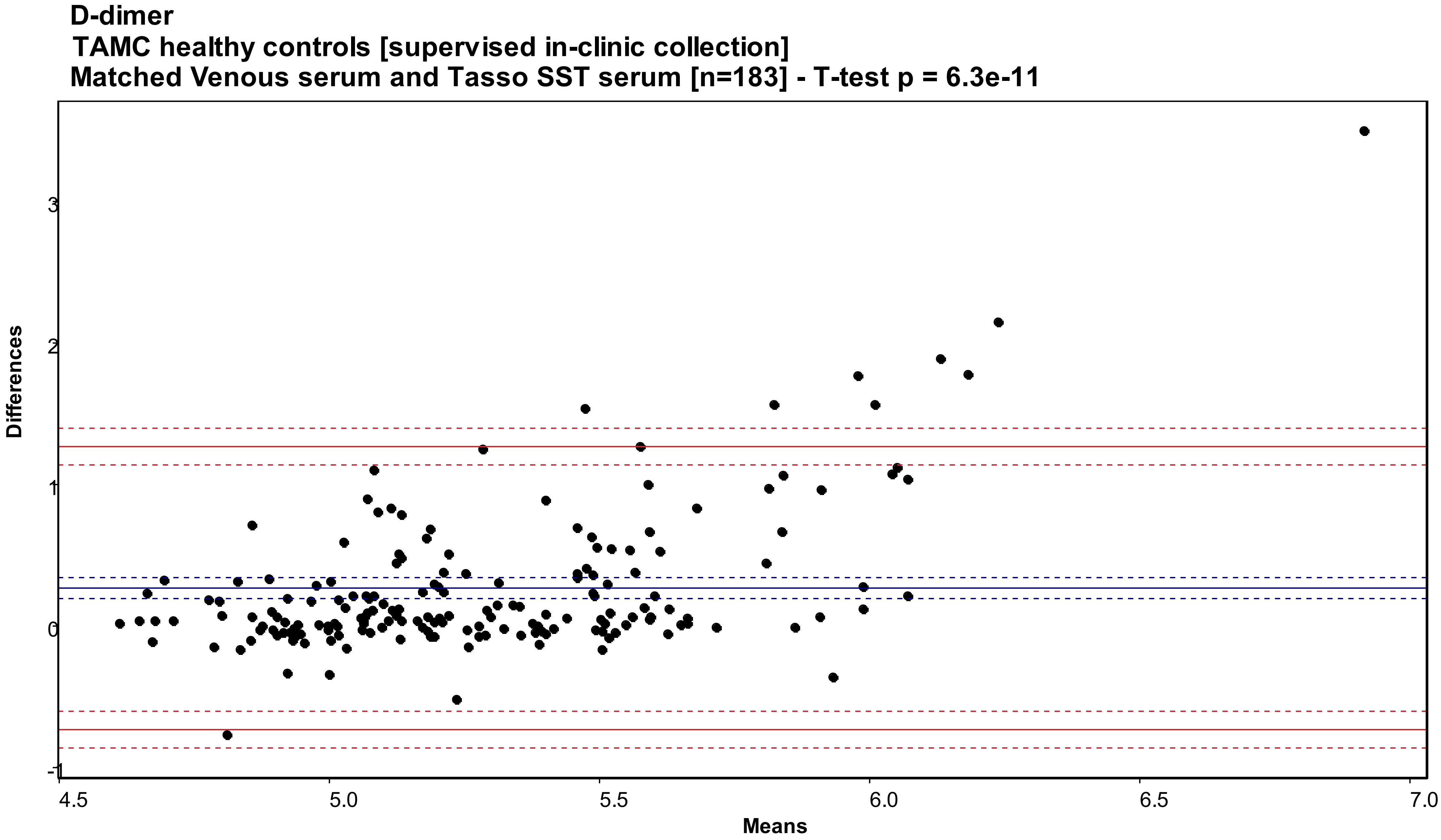
D-dimer
TAMC healthy controls [supervised in-clinic collection]
Matched Venous serum and Tasso SST serum [n=183] - T-test p = 6.3e-11
Differences
Means
-1
0
1
2
3
4.5
5.0
5.5
6.0
6.5
7.0

Ferritin
TAMC healthy controls [supervised in-clinic collection]
Matched Venous serum and Tasso SST serum [n=183] - T-test p = 1.74e-21

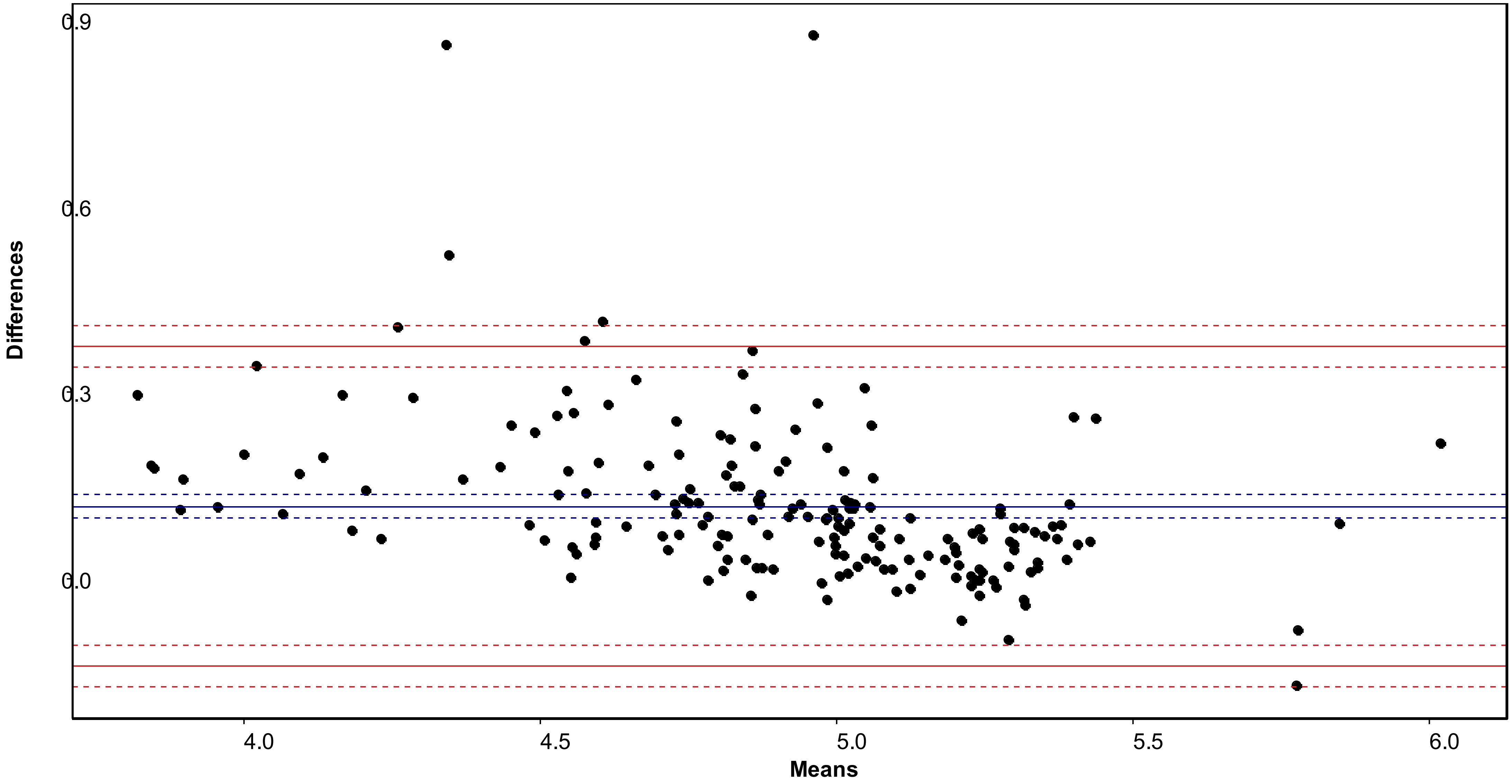

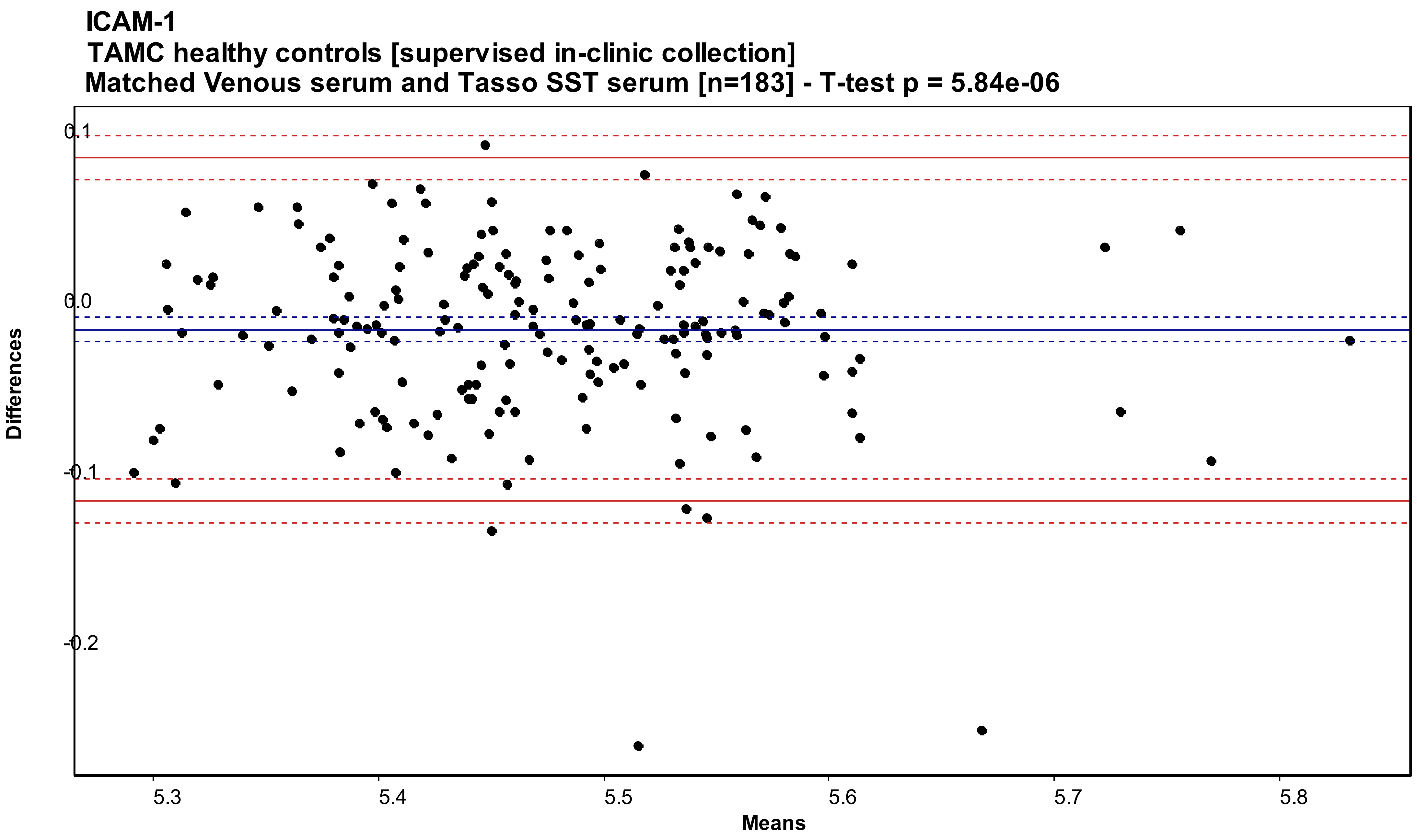
ICAM-1
TAMC healthy controls [supervised in-clinic collection]
Matched Venous serum and Tasso SST serum [n=183] - T-test p = 5.84e-06
0.1
0.0
-0.1
-0.2
Differences
5.3
5.4
5.5
5.6
5.7
5.8
Means

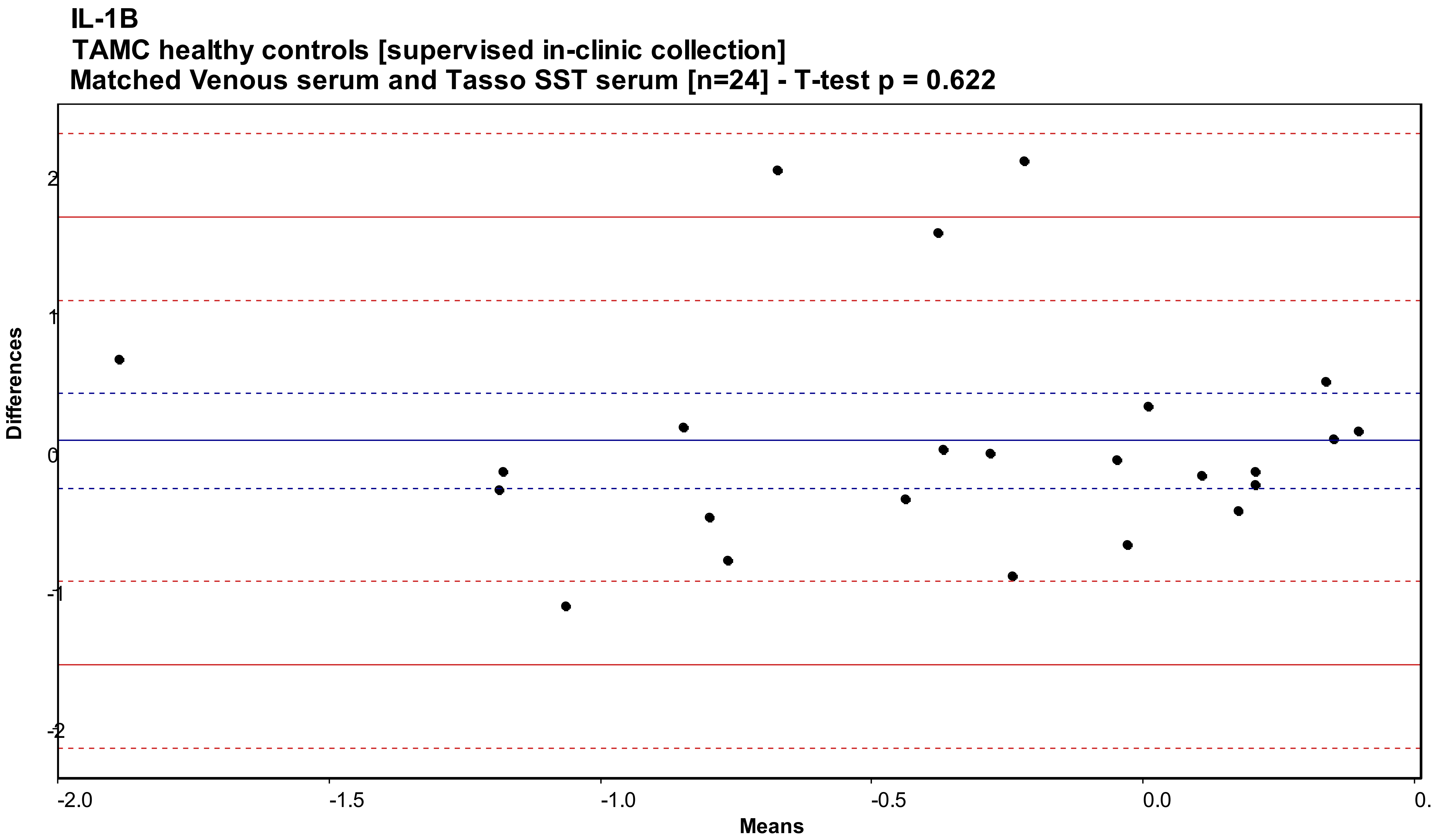
IL-1B
TAMC healthy controls [supervised in-clinic collection]
Matched Venous serum and Tasso SST serum [n=24] - T-test p = 0.622
Differences
2
1
0
-1
-2
-2.0
-1.5
-1.0
-0.5
0.0
0.
Means

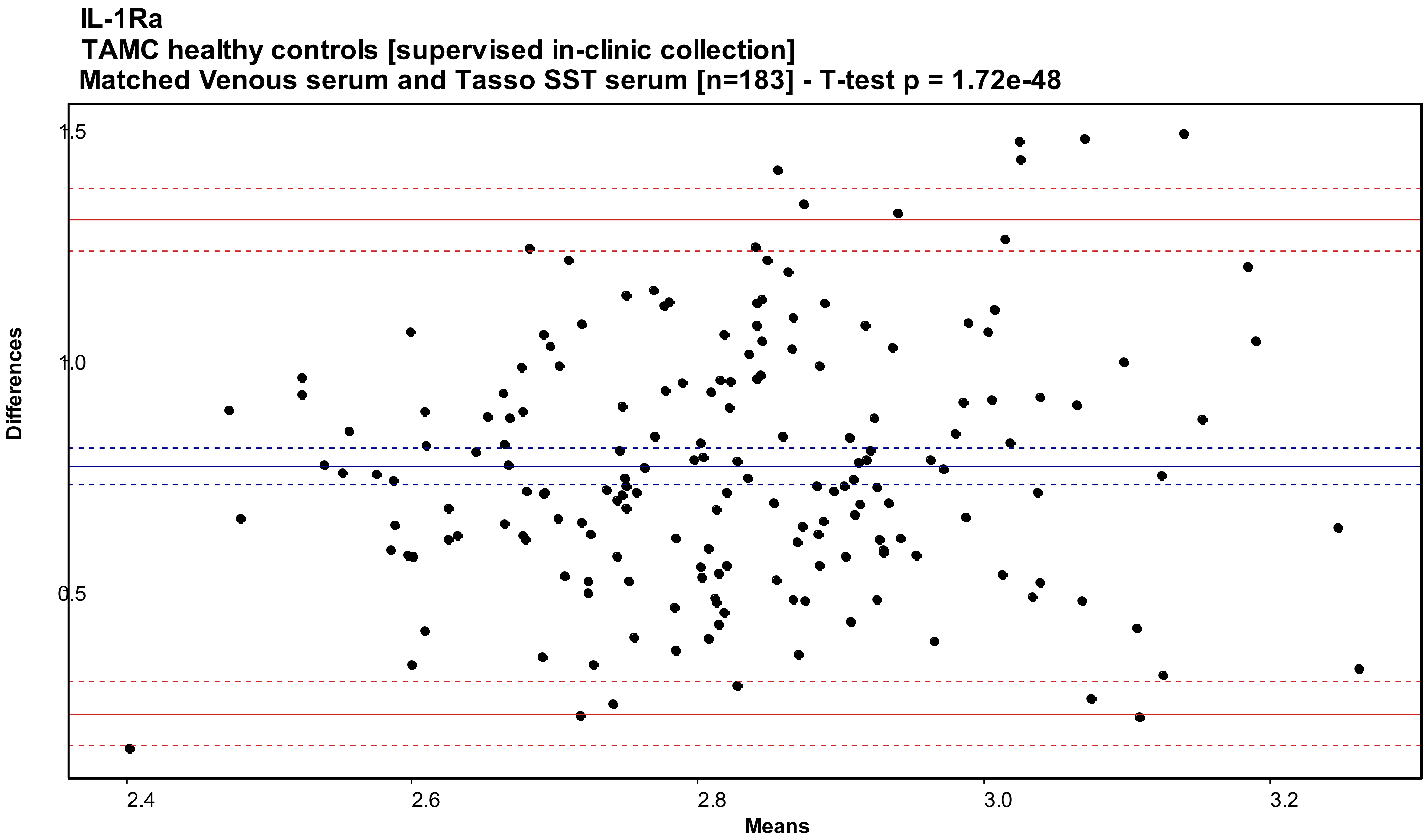
IL-1Ra
TAMC healthy controls [supervised in-clinic collection]
Matched Venous serum and Tasso SST serum [n=183] - T-test p = 1.72e-48
Differences
1.5
1.0
0.5
2.4
2.6
2.8
3.0
3.2
Means

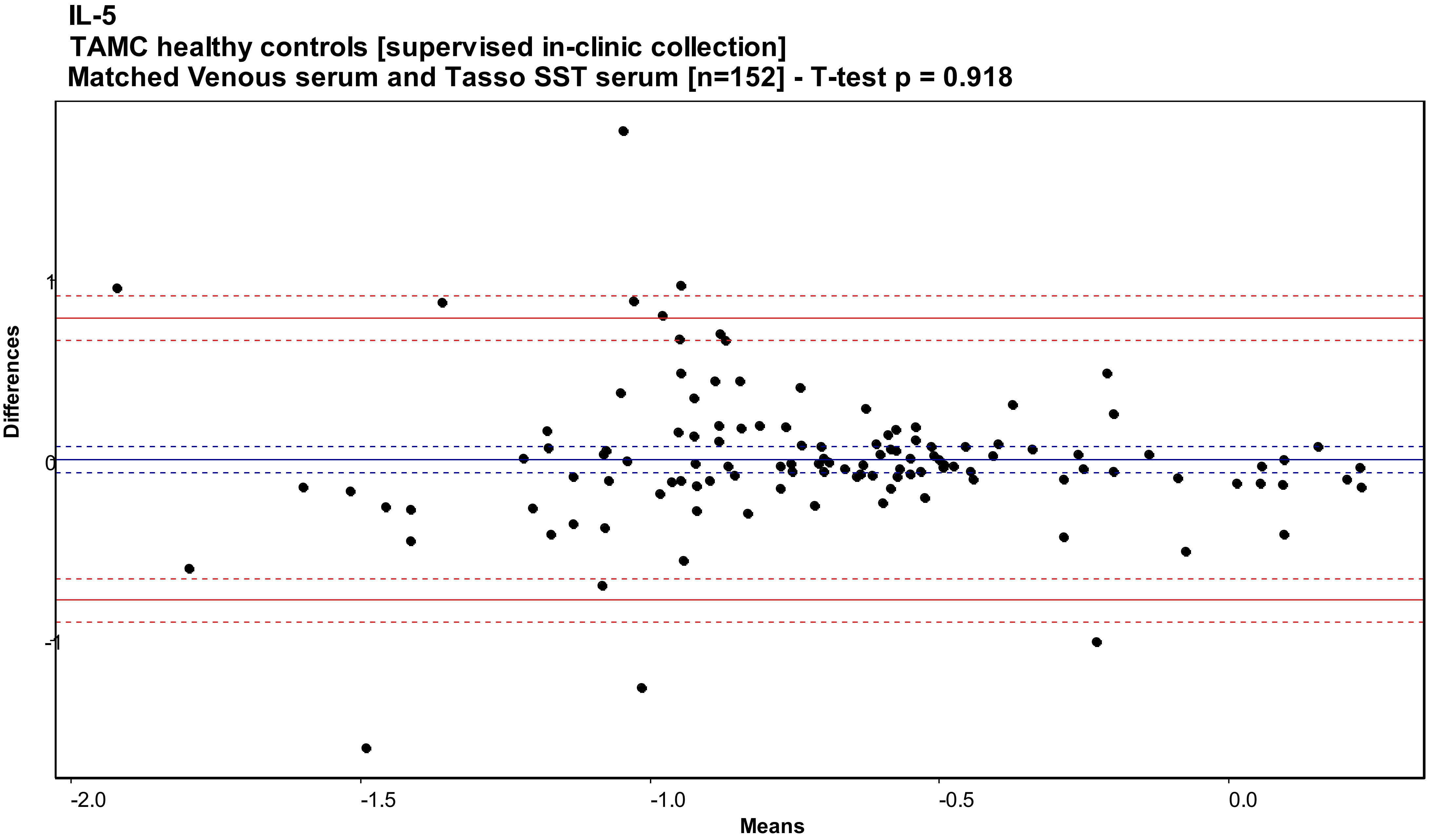
IL-5
TAMC healthy controls [supervised in-clinic collection]
Matched Venous serum and Tasso SST serum [n=152] - T-test p = 0.918
Differences
1
0
-1
-2.0
-1.5
-1.0
-0.5
0.0
Means

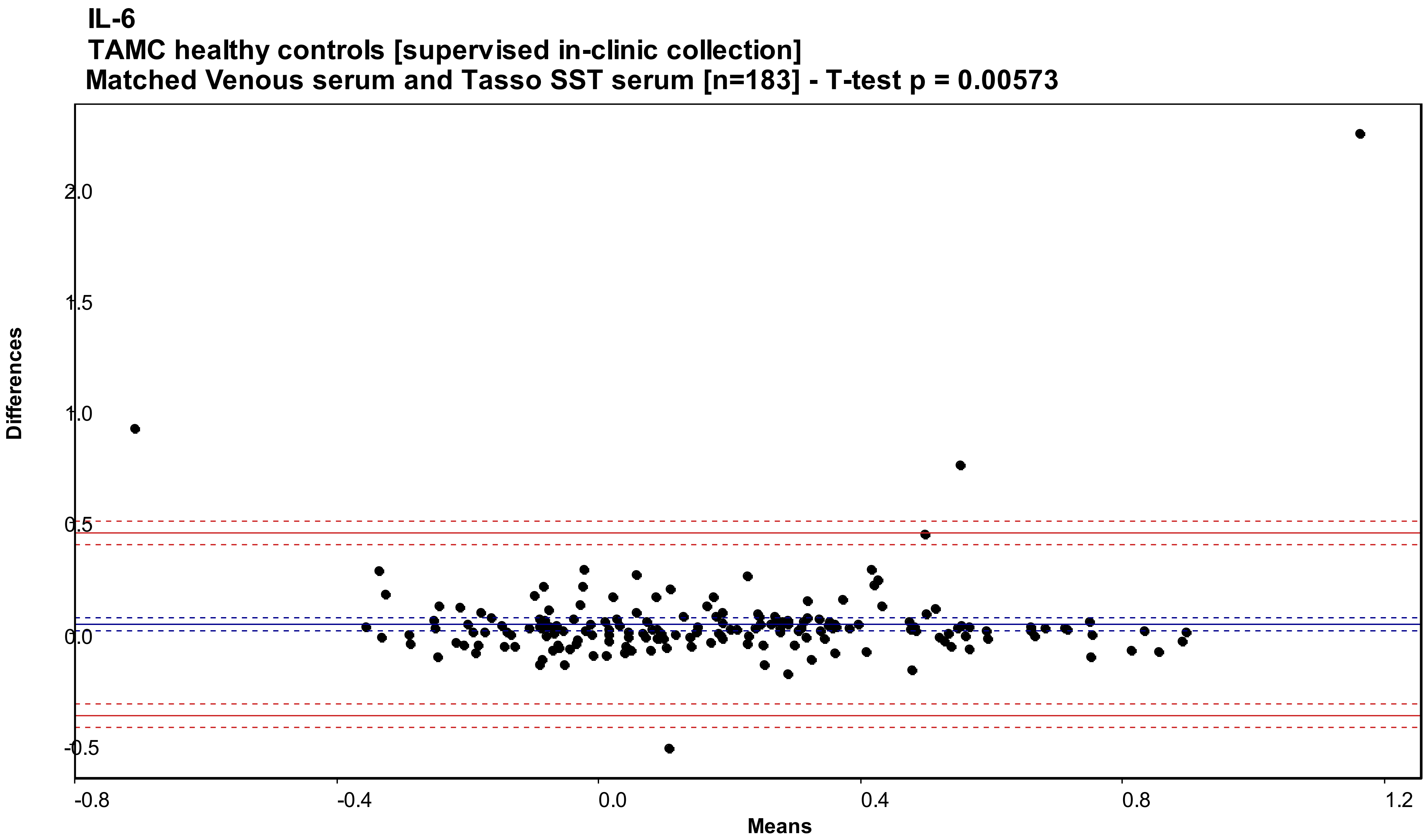
IL-6
TAMC healthy controls [supervised in-clinic collection]
Matched Venous serum and Tasso SST serum [n=183] - T-test p = 0.00573
Differences
2.0
1.5
1.0
0.5
0.0
-0.5
-0.8
-0.4
0.0
0.4
0.8
1.2
Means

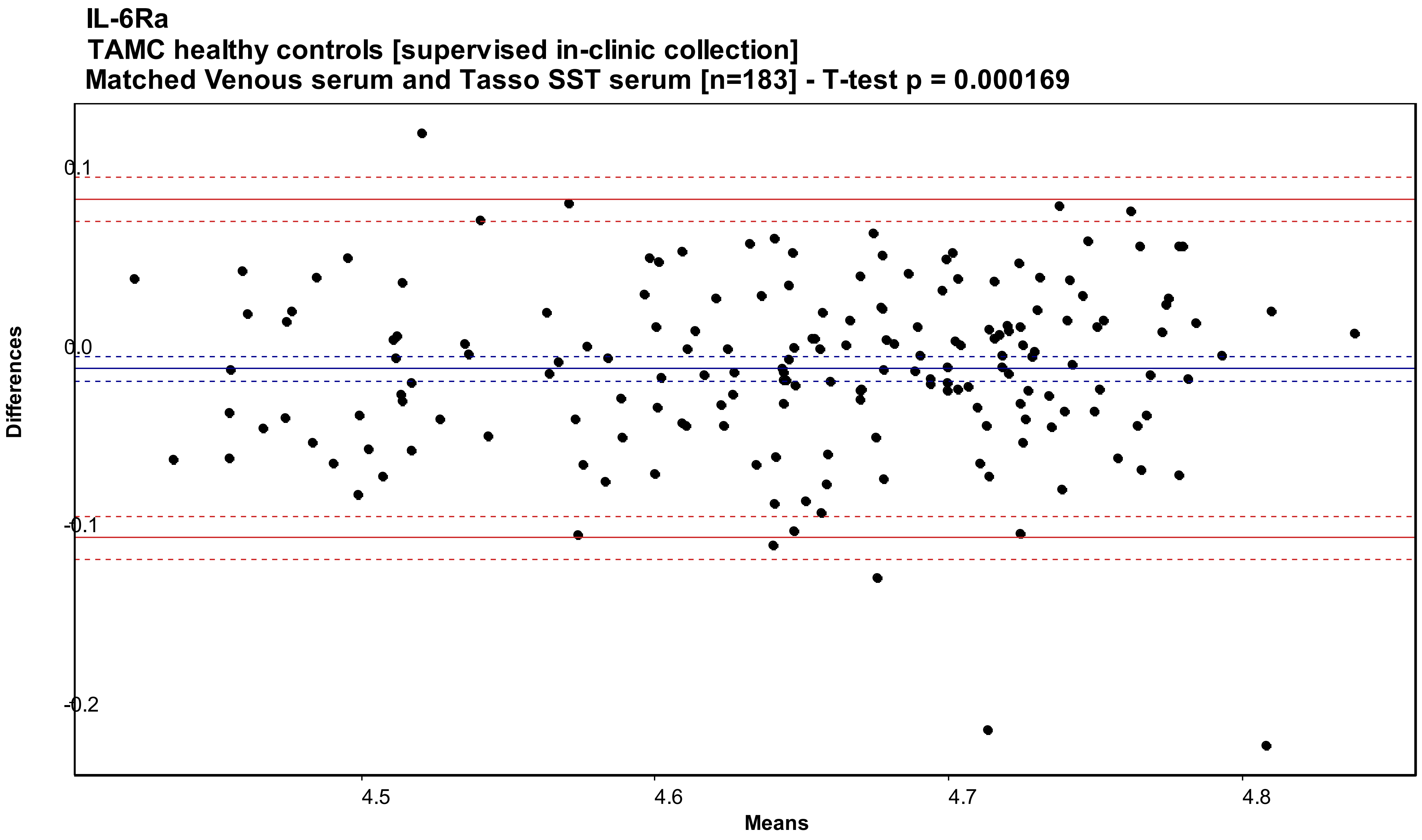
IL-6Ra
TAMC healthy controls [supervised in-clinic collection]
Matched Venous serum and Tasso SST serum [n=183] - T-test p = 0.000169
Differences
0.1
0.0
-0.1
-0.2
4.5
4.6
4.7
4.8
Means

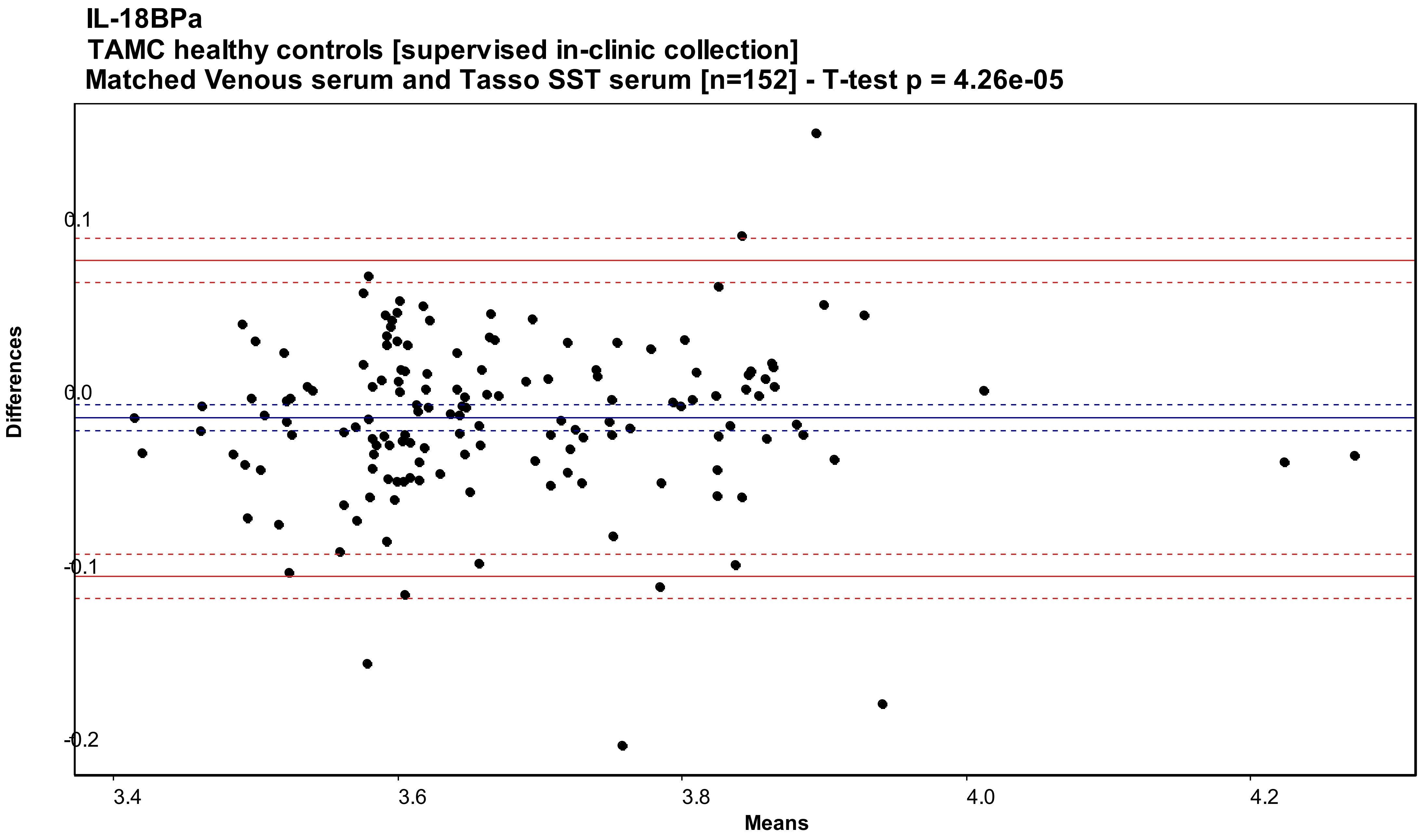
IL-18BPa
TAMC healthy controls [supervised in-clinic collection]
Matched Venous serum and Tasso SST serum [n=152] - T-test p = 4.26e-05
Differences
0.1
0.0
-0.1
-0.2
3.4
3.6
3.8
4.0
4.2
Means

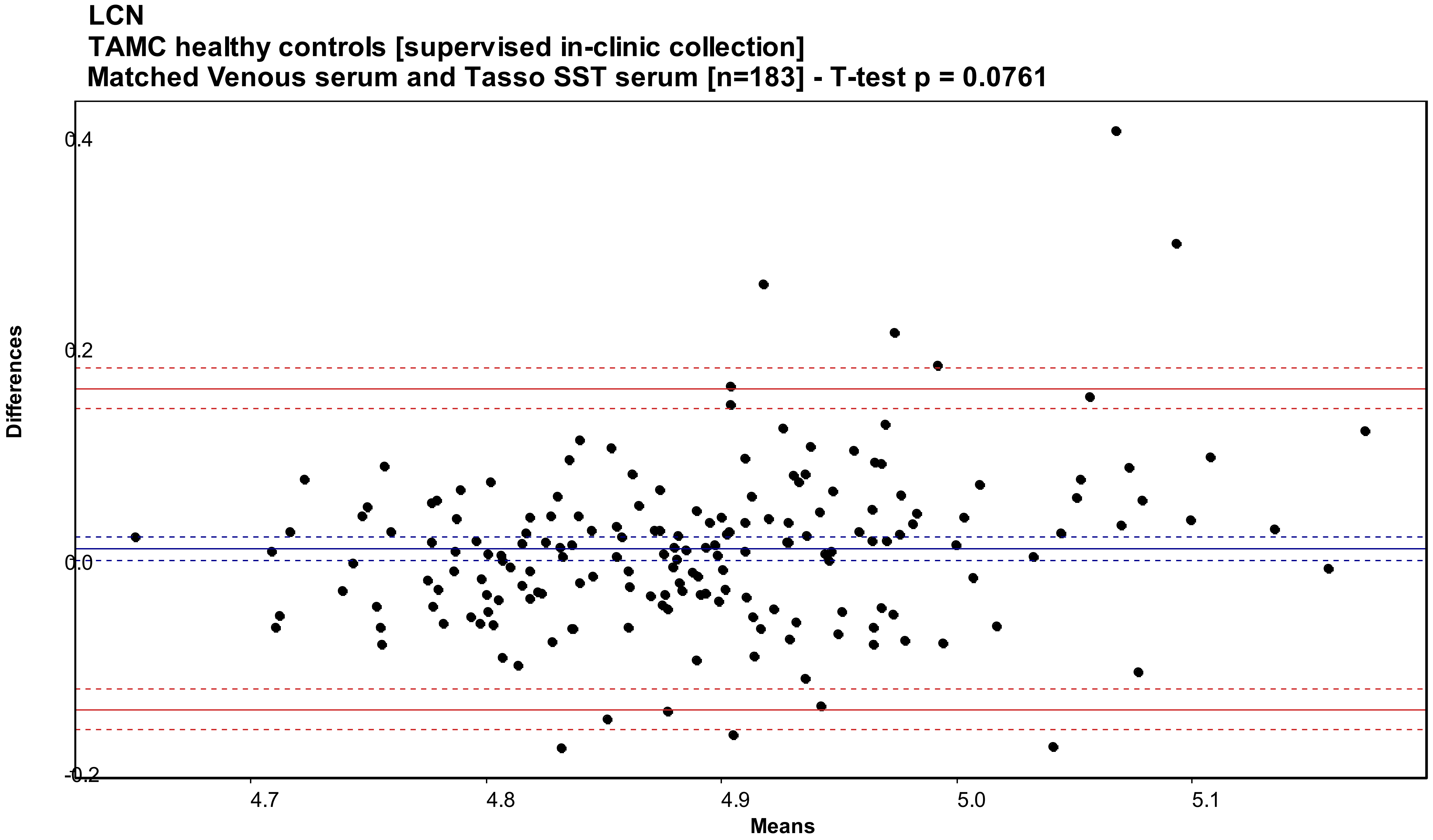

LCN
TAMC healthy controls [supervised in-clinic collection]
Matched Venous serum and Tasso SST serum [n=183] - T-test p = 0.0761
Differences
0.4
0.2
0.0
-0.2
4.7
4.8
4.9
5.0
5.1
Means

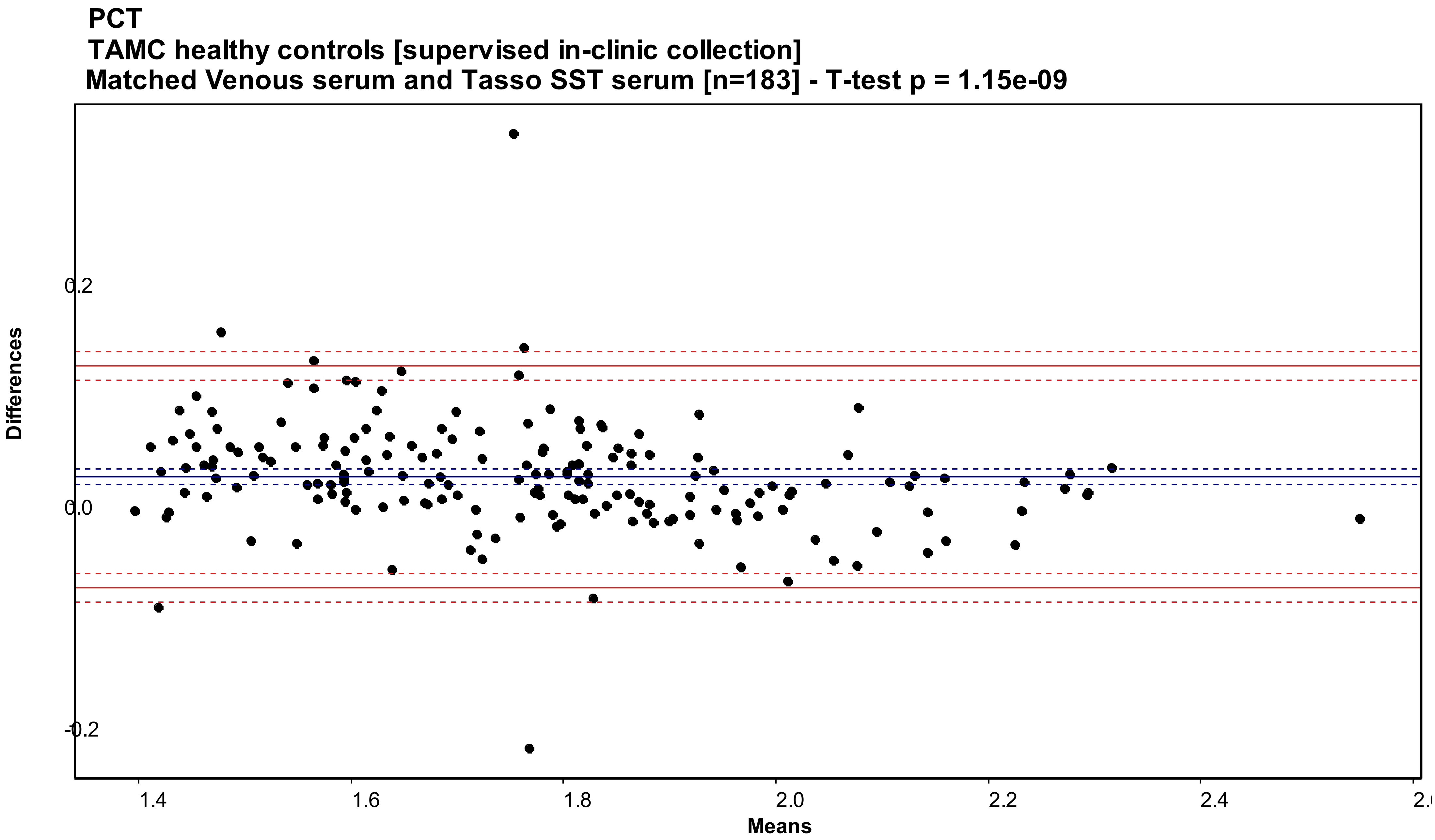
PCT
TAMC healthy controls [supervised in-clinic collection]
Matched Venous serum and Tasso SST serum [n=183] - T-test p = 1.15e-09
Differences
0.2
0.0
-0.2
1.4
1.6
1.8
2.0
2.2
2.4
Means

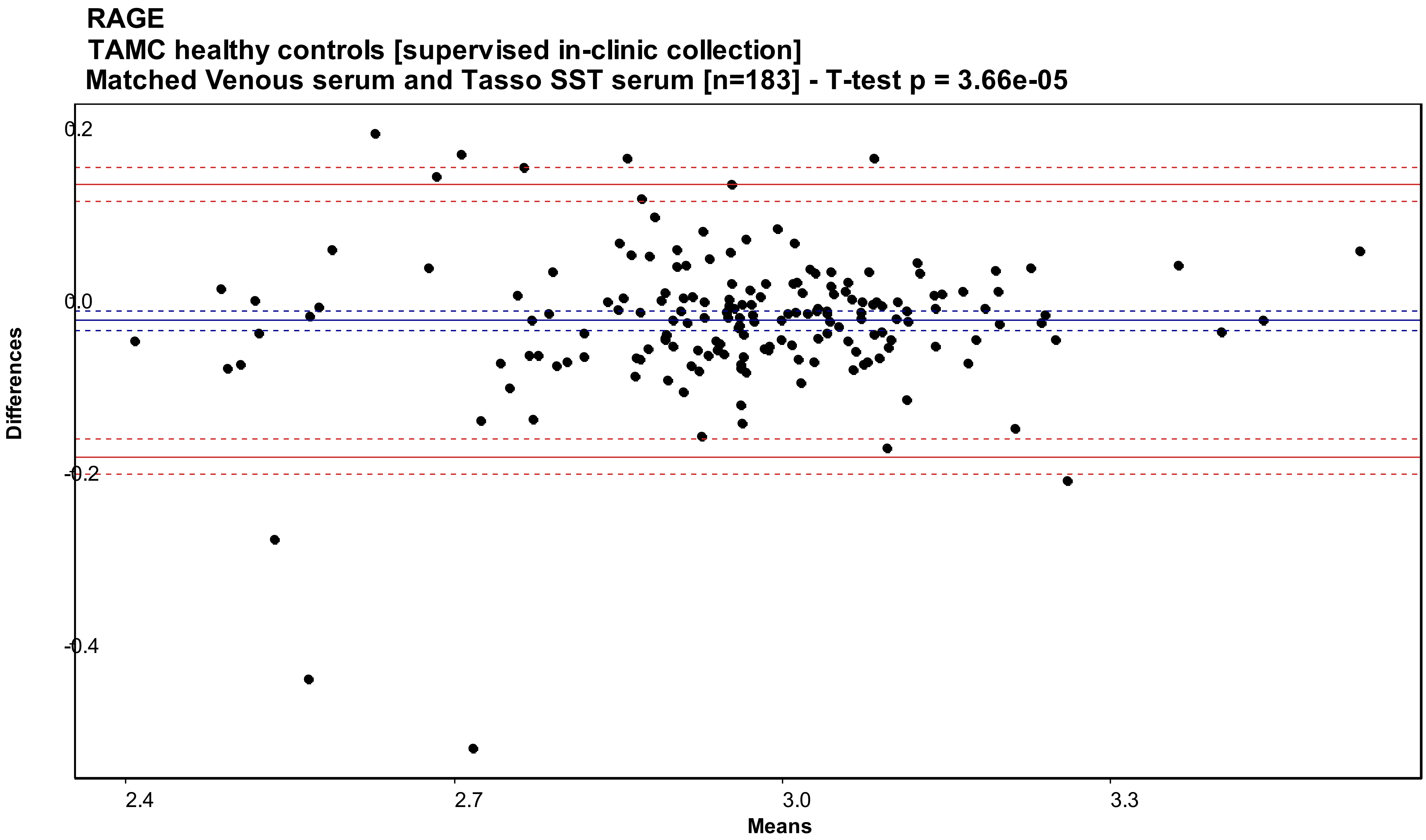
RAGE
TAMC healthy controls [supervised in-clinic collection]
Matched Venous serum and Tasso SST serum [n=183] - T-test p = 3.66e-05
0.2
0.0
-0.2
-0.4
Differences
2.4
2.7
3.0
3.3
Means

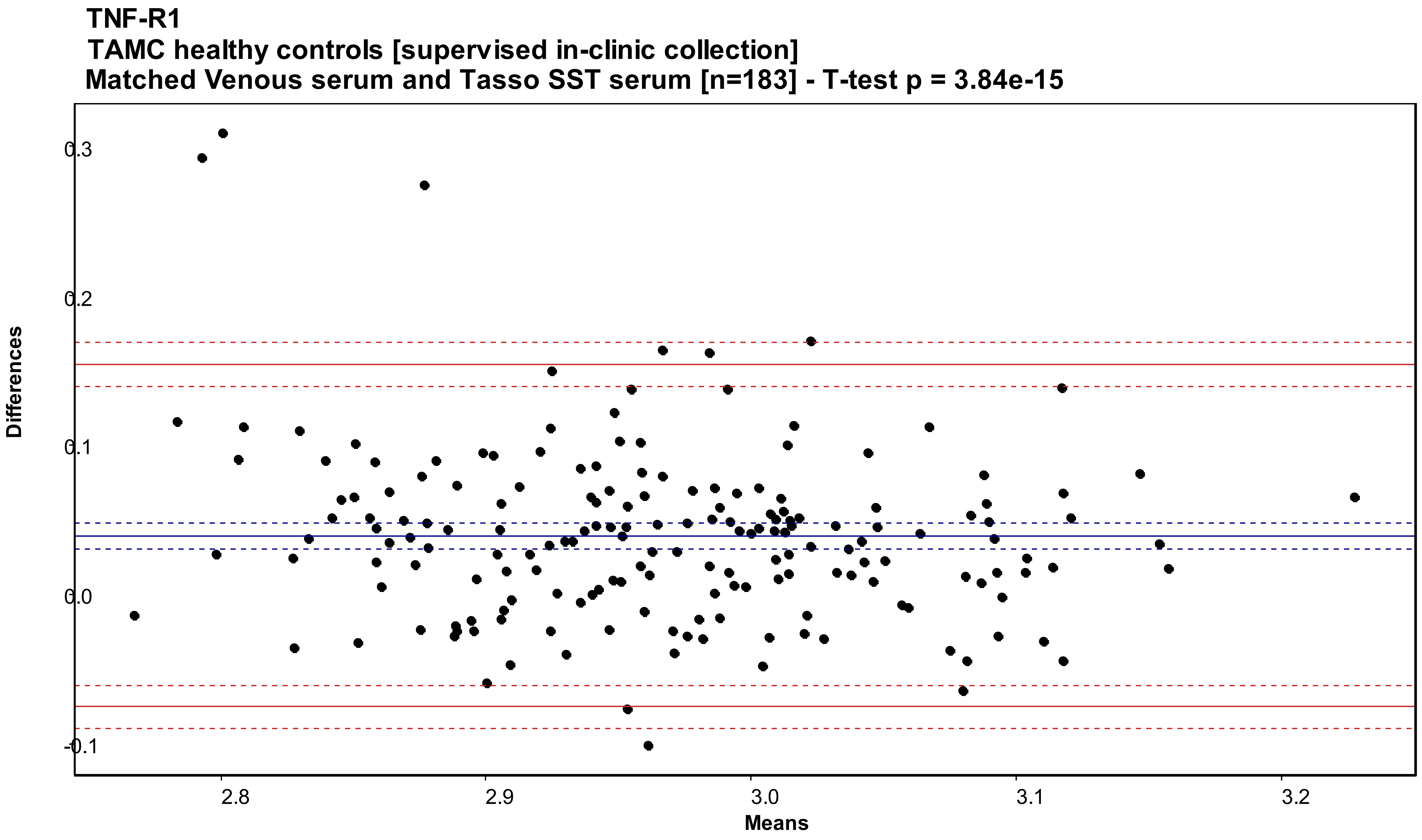
TNF-R1
TAMC healthy controls [supervised in-clinic collection]
Matched Venous serum and Tasso SST serum [n=183] - T-test p = 3.84e-15
Differences
Means
0.3
0.2
0.1
0.0
-0.1
2.8
2.9
3.0
3.1
3.2

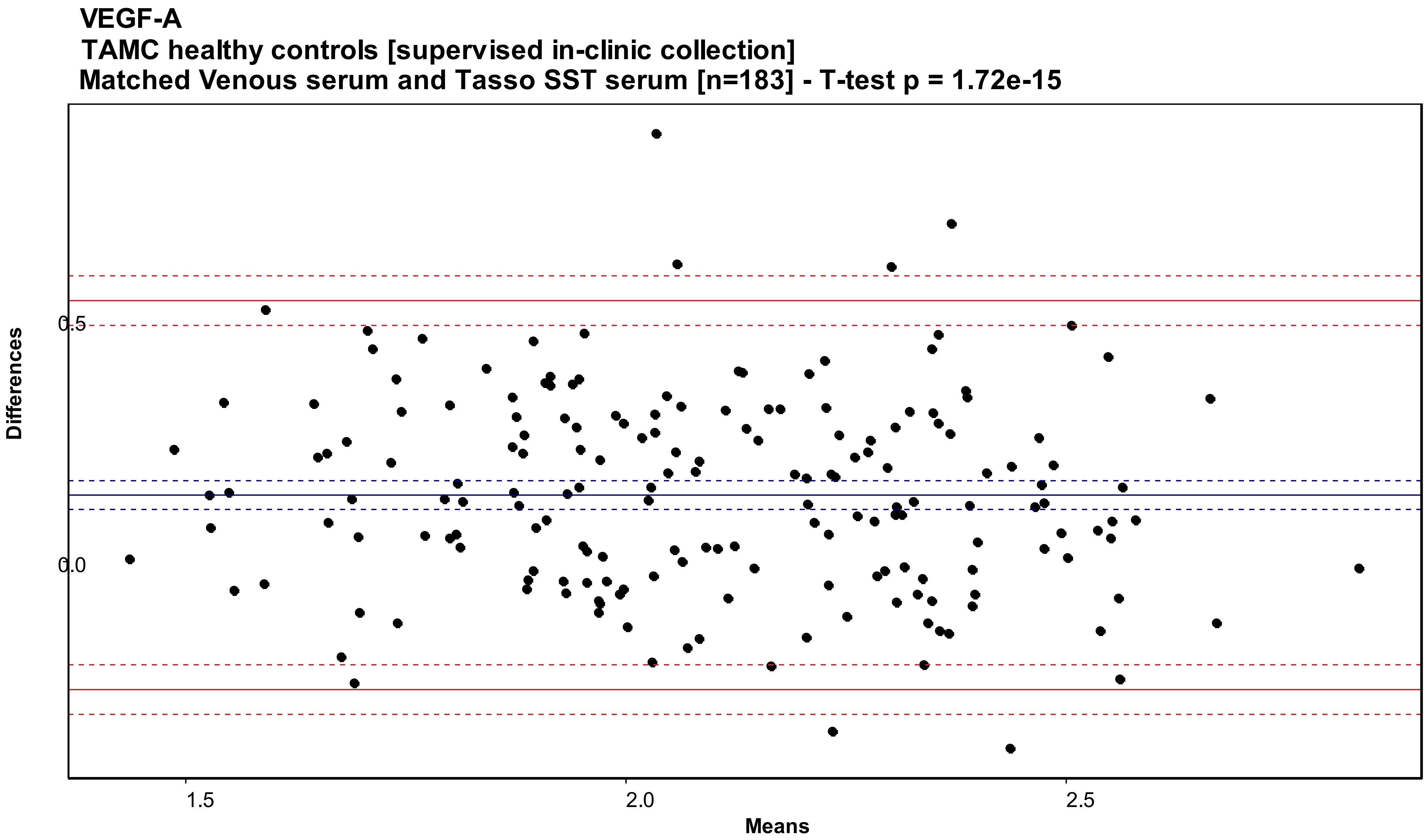
VEGF-A
TAMC healthy controls [supervised in-clinic collection]
Matched Venous serum and Tasso SST serum [n=183] - T-test p = 1.72e-15
Differences
0.5
0.0
1.5
2.0
2.5
Means
