# Supplementary material for: Assessing the use of a micro-sampling device for measuring blood protein levels in healthy subjects and COVID-19 patients
Source: PLoS One. 2022 Aug 10;17(8):e0272572. doi: 10.1371/journal.pone.0272572 (PMC9365123; doi:10.1371/journal.pone.0272572)
Supplement: S4 Fig — Baseline concentrations of selected proteins in Tasso SST serum samples from healthy controls and non-hospitalized COVID-19 patients. Data are shown for the supervised, in-clinic collection protocol at TAMC (with rapid processing), the unsupervised, at-home self-collection protocol at NHRC (with potentially delayed processing), and for both protocols combined. Concentrations were log10 transformed. Significance values for Mann-Whitney U tests between COVID-19 patients and healthy controls are abbreviated as follows: n.s. not significant; * p<0.05; ** p<0.01; *** p<0.001; **** p<0.0001. (PDF) [file pone.0272572.s007.pdf]

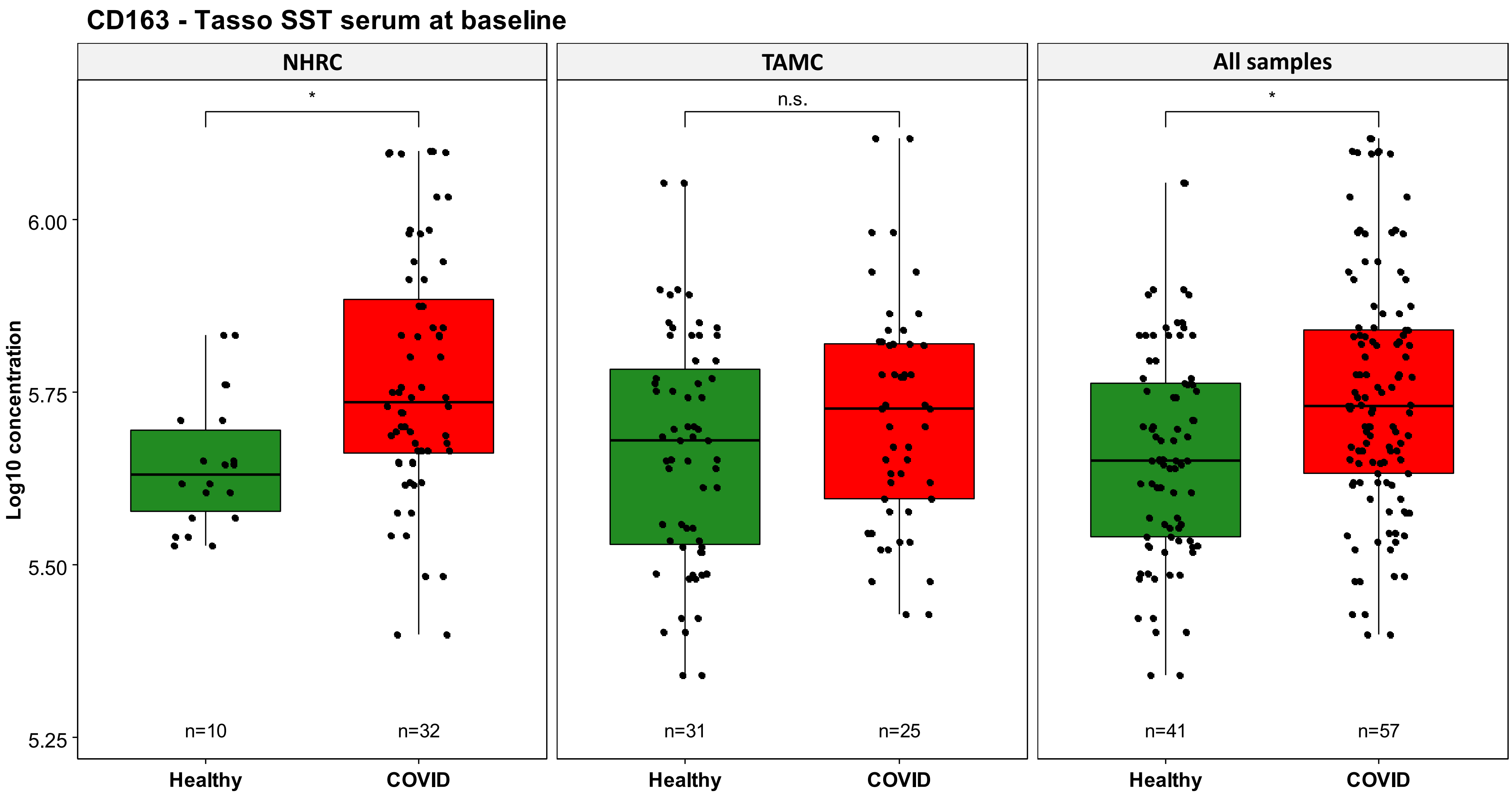
CD163 - Tasso SST serum at baseline
NHRC
TAMC
All samples
*
n.s.
*
Log10 concentration
6.00
5.75
5.50
5.25
n=10
n=32
n=31
n=25
n=41
n=57
Healthy
COVID
Healthy
COVID
Healthy
COVID

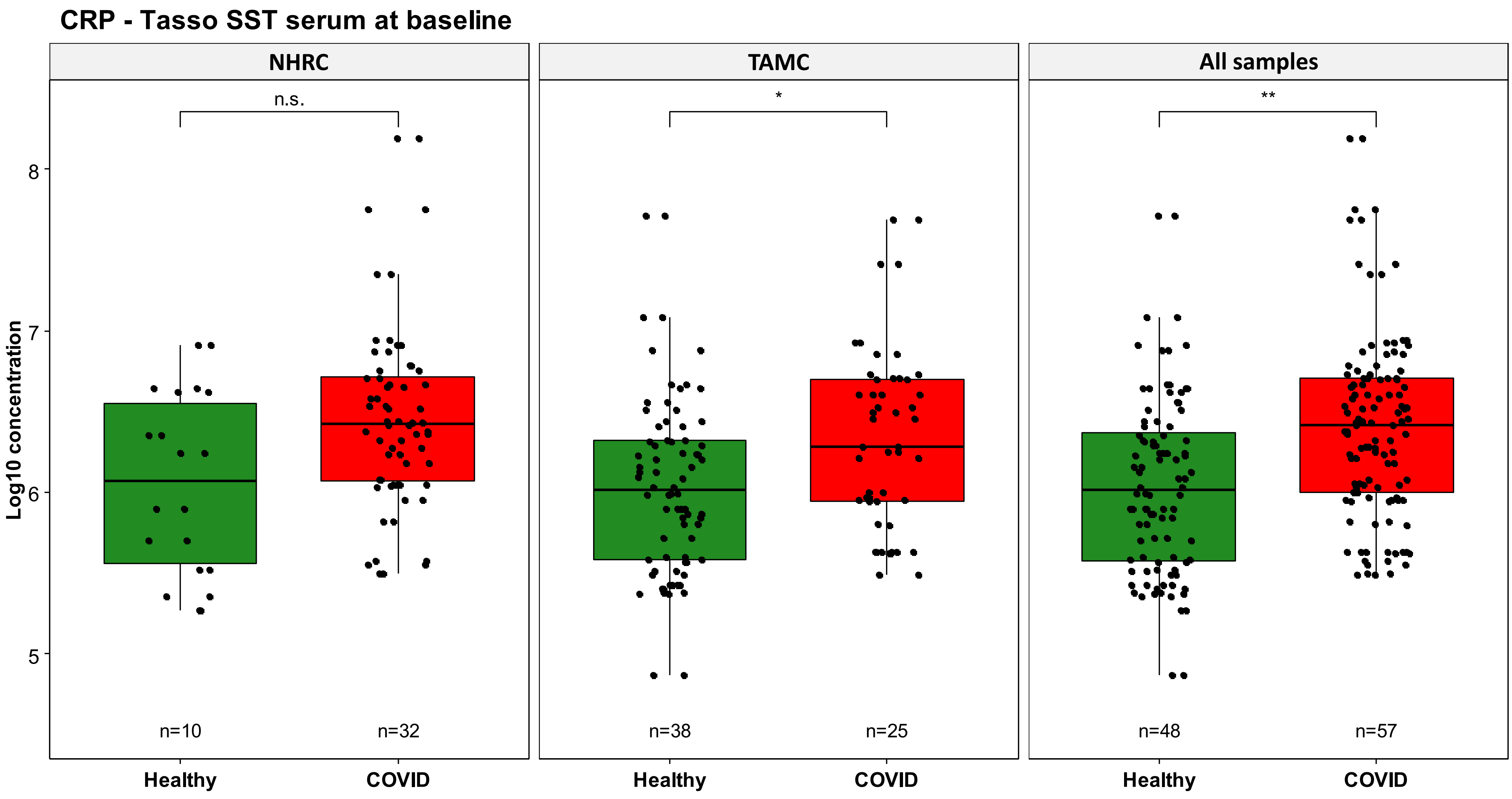

CRP - Tasso SST serum at baseline
NHRC
TAMC
All samples
n.s.
*
**
Log10 concentration
8
7
6
5
n=10
n=32
n=38
n=25
n=48
n=57
Healthy
COVID
Healthy
COVID
Healthy
COVID

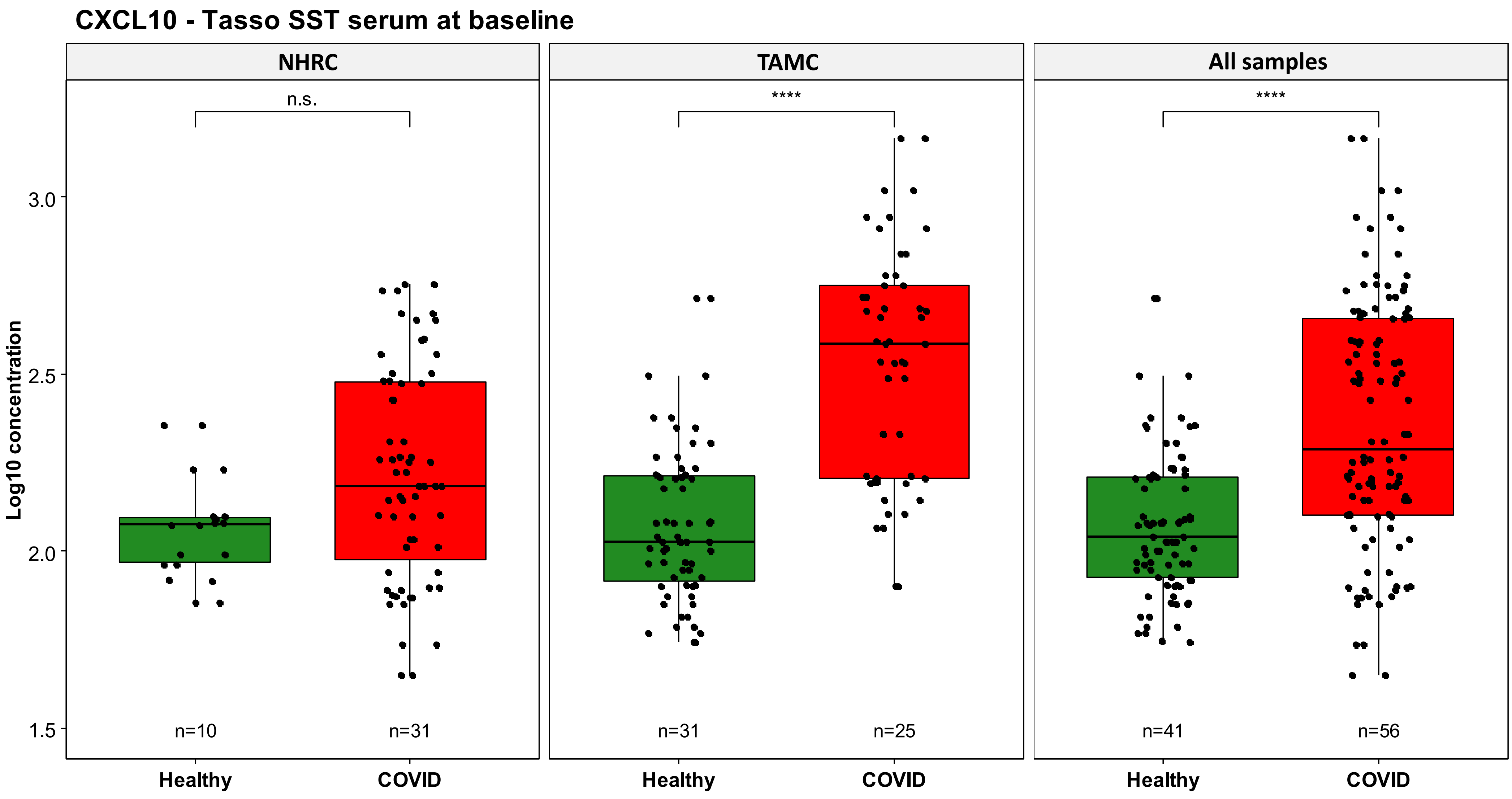
CXCL10 - Tasso SST serum at baseline
NHRC
TAMC
All samples
n.s.
****
****
Log10 concentration
3.0
2.5
2.0
1.5
n=10
n=31
n=31
n=25
n=41
n=56
Healthy
COVID
Healthy
COVID
Healthy
COVID

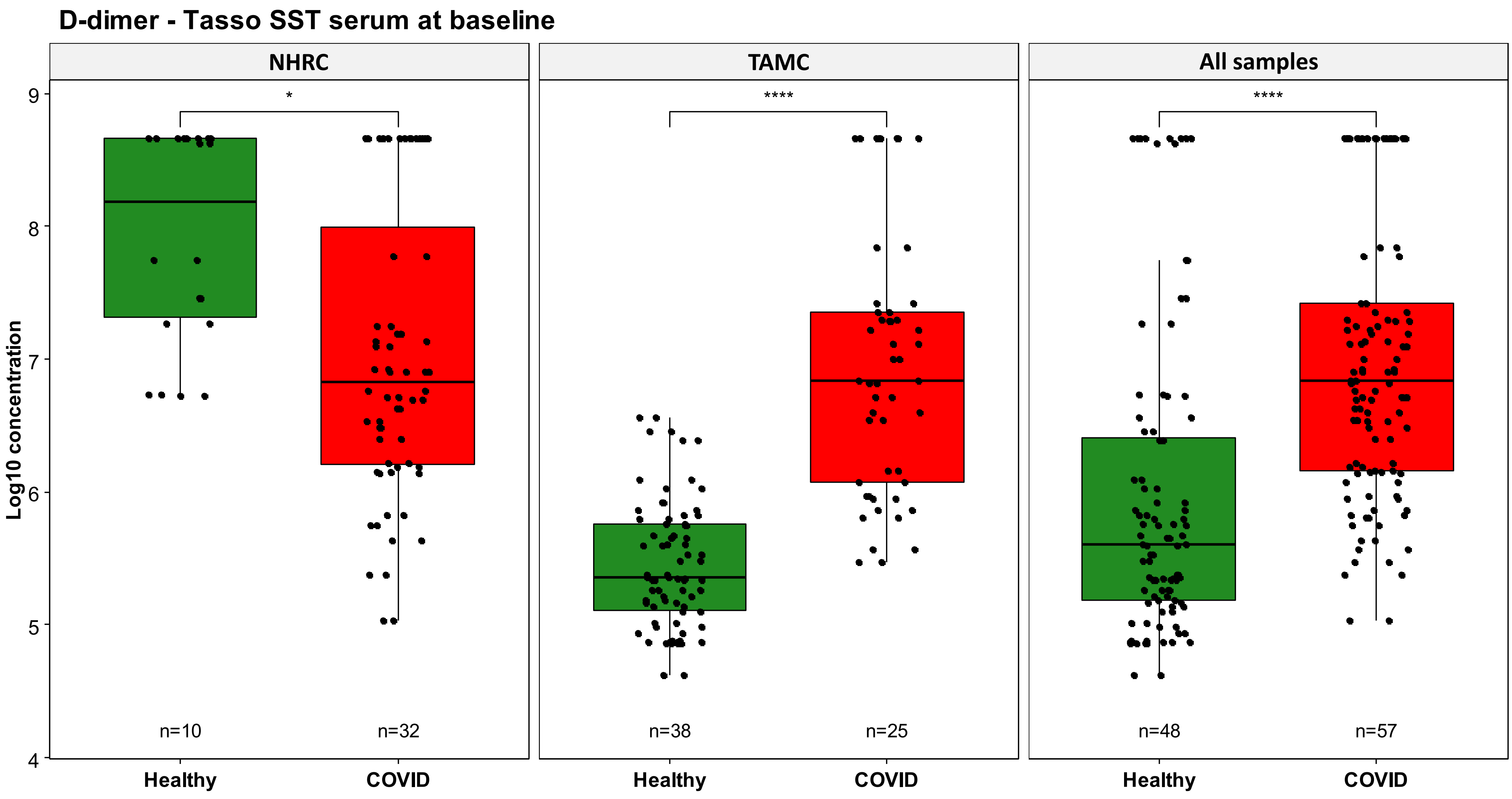

D-dimer - Tasso SST serum at baseline
NHRC
TAMC
All samples
Log10 concentration
9
8
7
6
5
4
*
****
****
n=10
n=32
n=38
n=25
n=48
n=57
Healthy
COVID
Healthy
COVID
Healthy
COVID

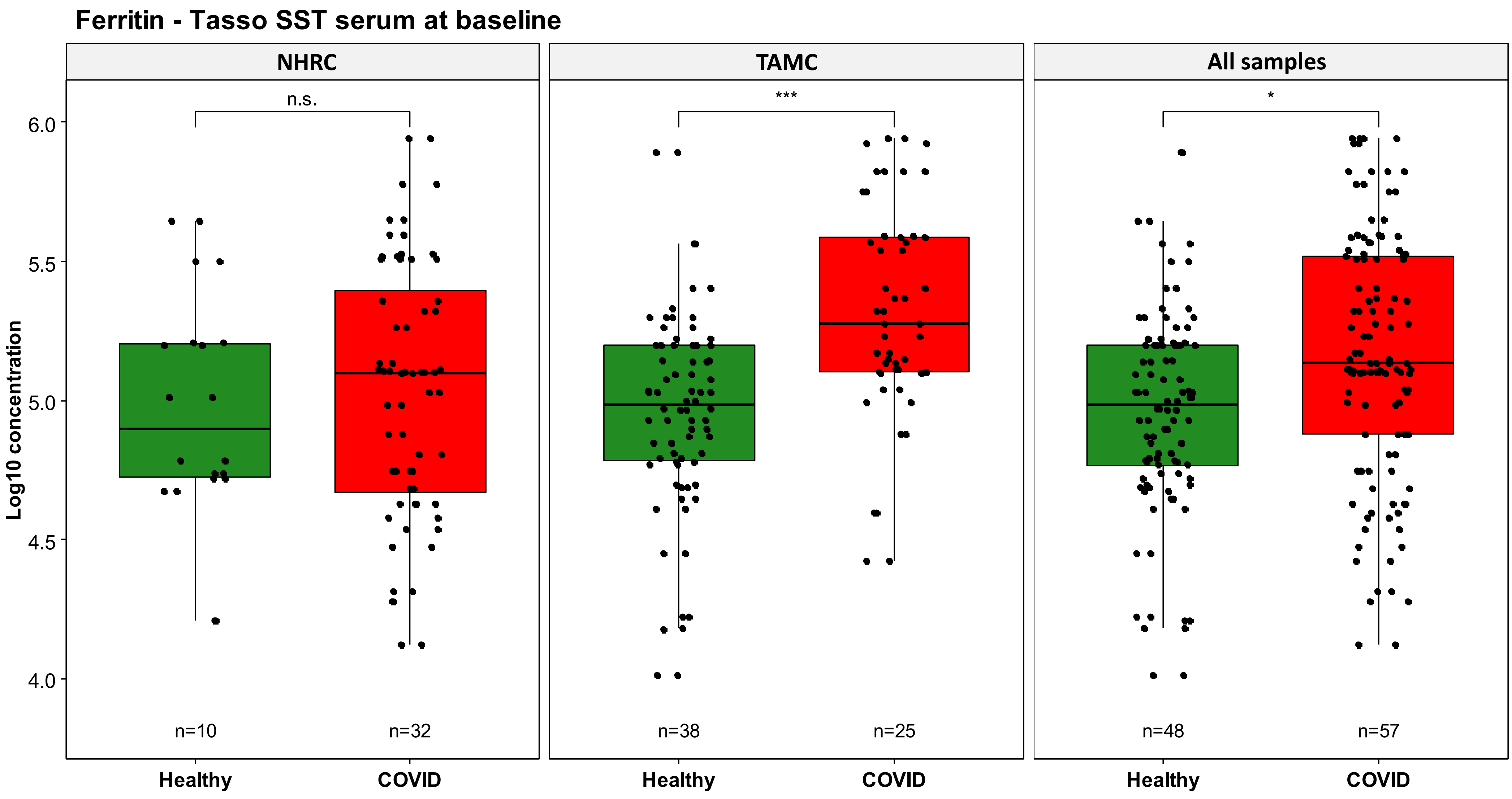

Ferritin - Tasso SST serum at baseline
NHRC
TAMC
All samples
n.s.
***
*
6.0
5.5
5.0
4.5
4.0
Log10 concentration
n=10
n=32
n=38
n=25
n=48
n=57
Healthy
COVID
Healthy
COVID
Healthy
COVID

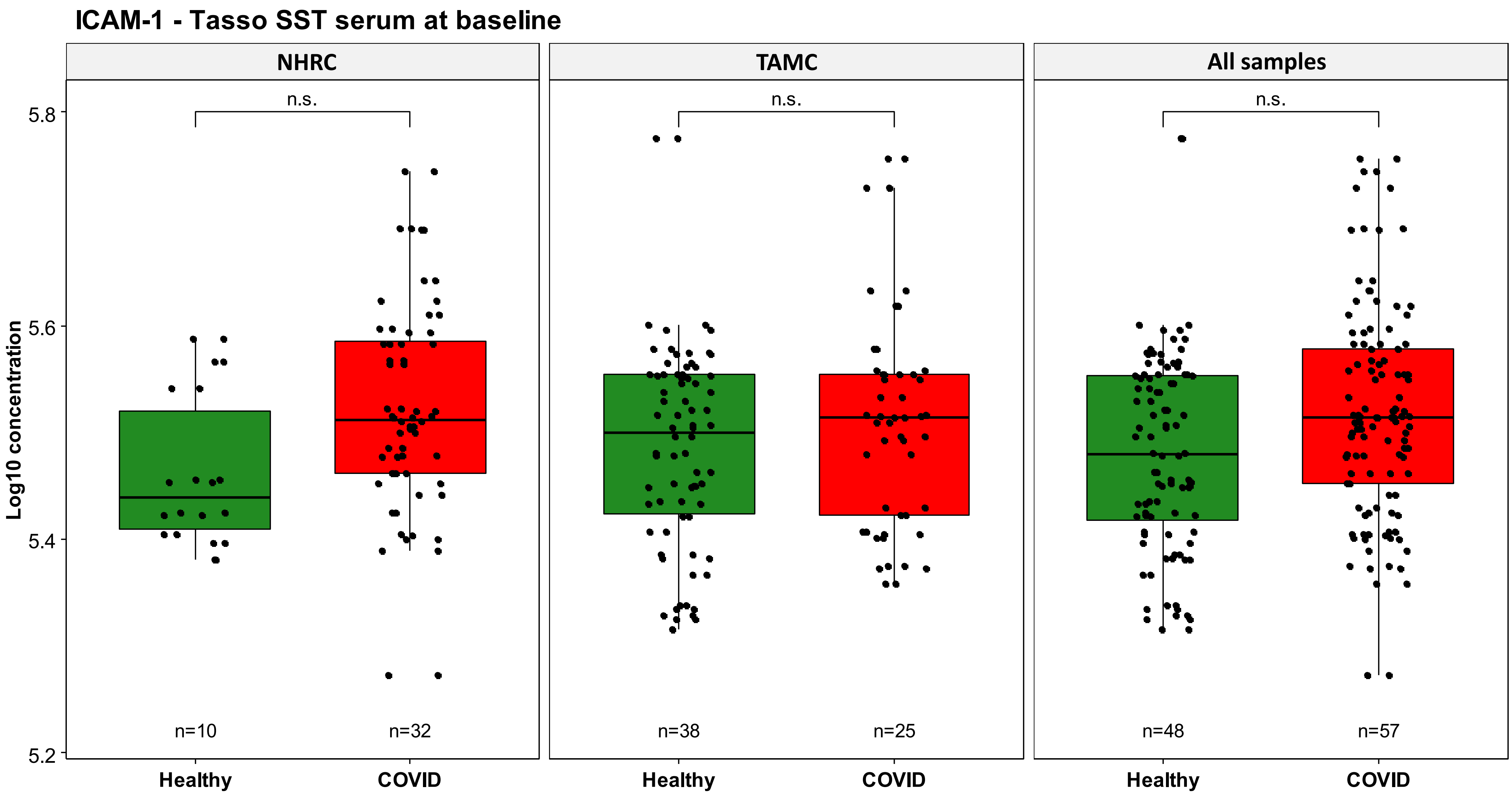
ICAM-1 - Tasso SST serum at baseline
NHRC
TAMC
All samples
n.s.
n.s.
n.s.
Log10 concentration
5.8
5.6
5.4
5.2
n=10
n=32
n=38
n=25
n=48
n=57
Healthy
COVID
Healthy
COVID
Healthy
COVID

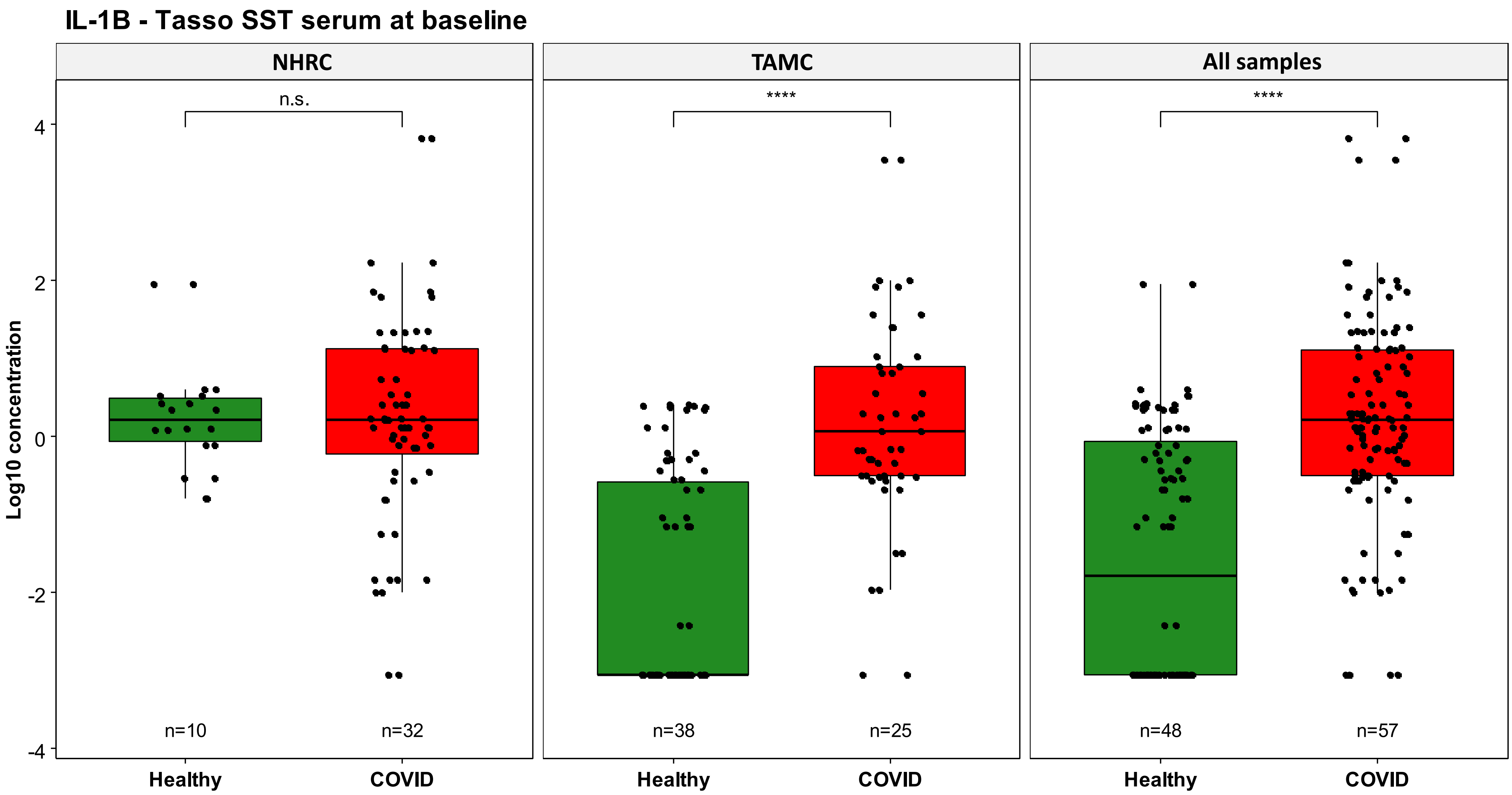
IL-1B - Tasso SST serum at baseline
NHRC
TAMC
All samples
n.s.
****
****
Log10 concentration
4
2
0
-2
-4
n=10
n=32
n=38
n=25
n=48
n=57
Healthy
COVID
Healthy
COVID
Healthy
COVID

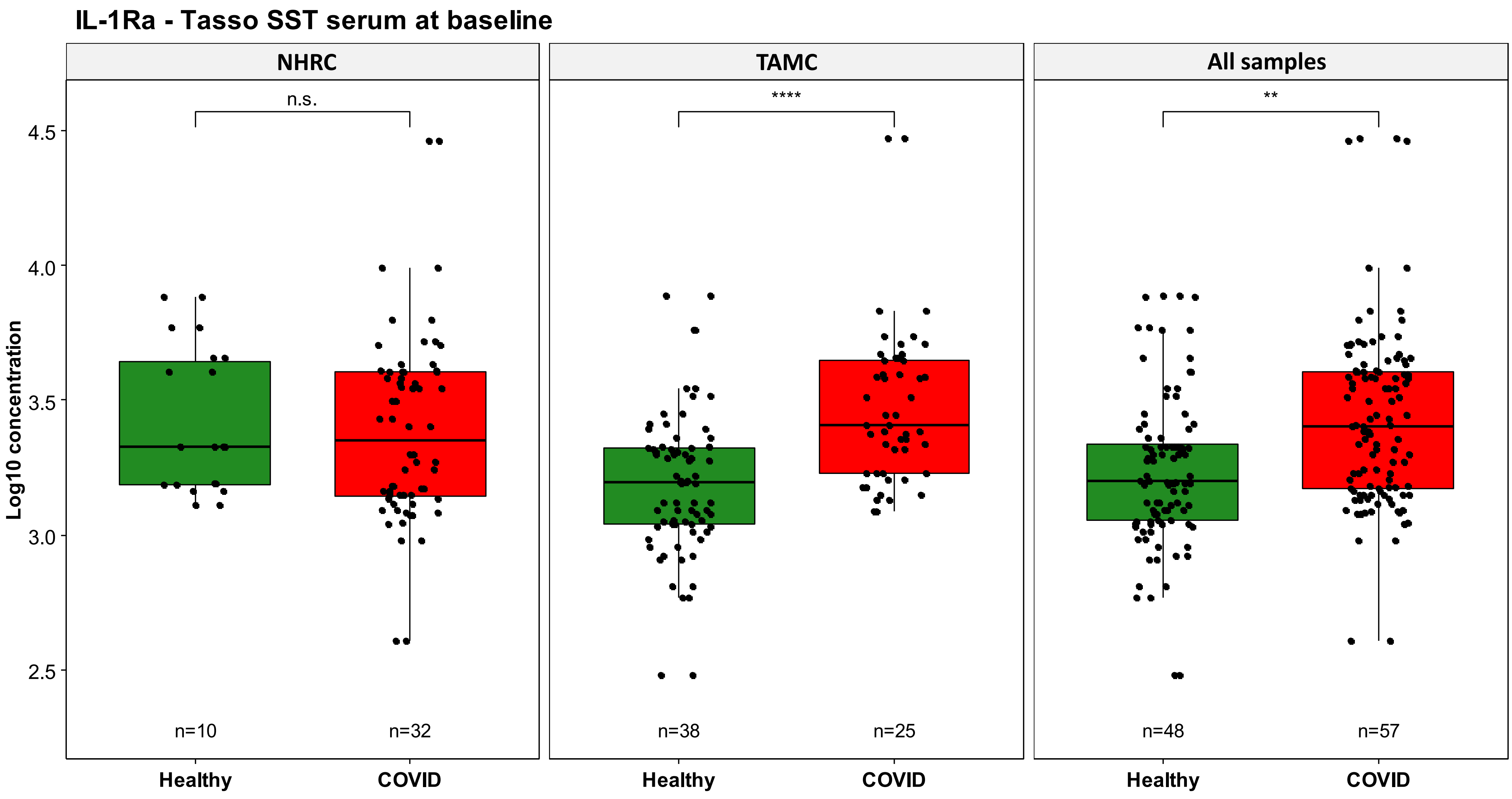

IL-1Ra - Tasso SST serum at baseline
NHRC
TAMC
All samples
n.s.
****
**
Log10 concentration
4.5
4.0
3.5
3.0
2.5
n=10
n=32
n=38
n=25
n=48
n=57
Healthy
COVID

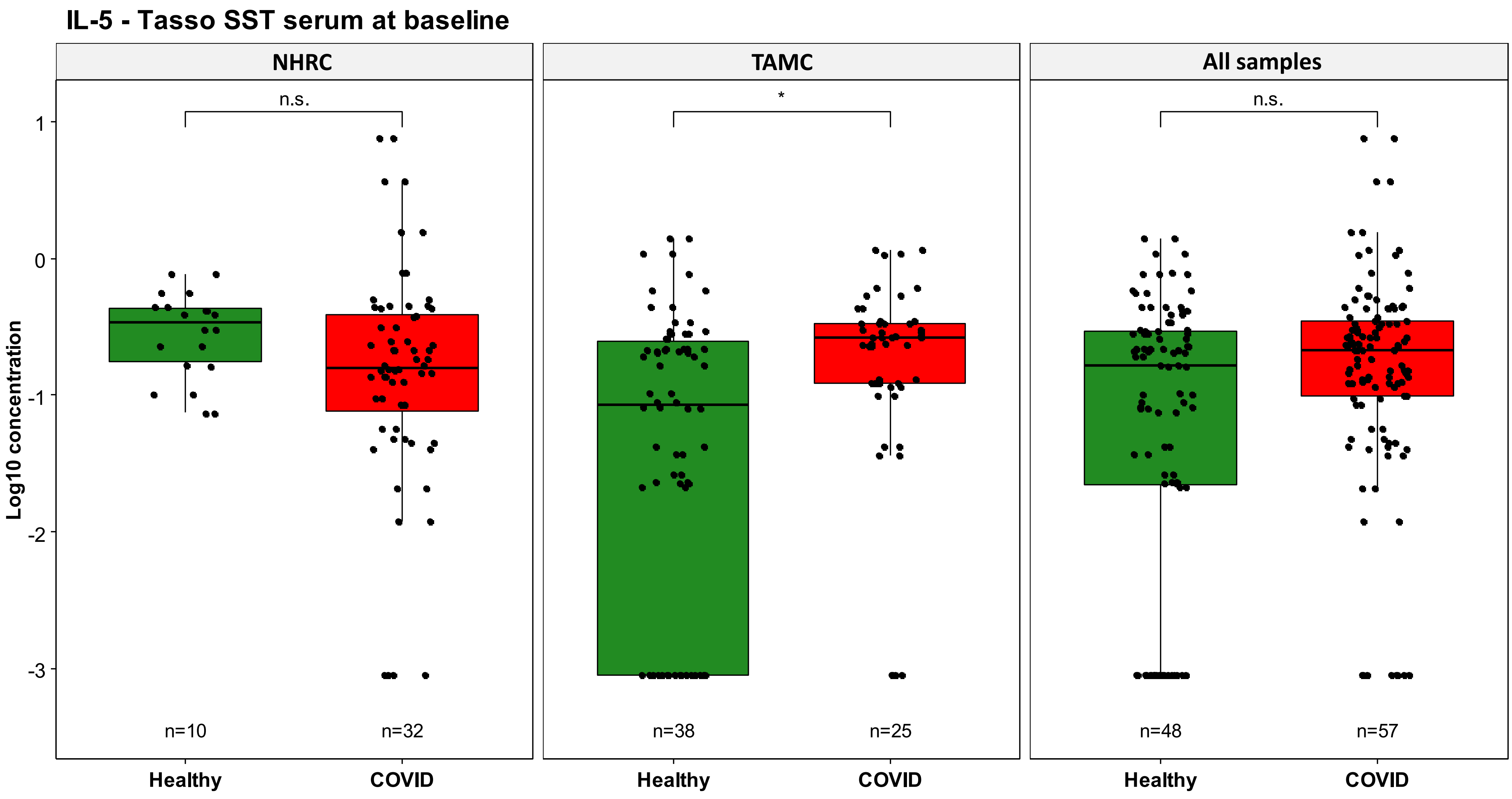
IL-5 - Tasso SST serum at baseline
NHRC
TAMC
All samples
n.s.
*
n.s.
Log10 concentration
1
0
-1
-2
-3
n=10
n=32
n=38
n=25
n=48
n=57
Healthy
COVID
Healthy
COVID
Healthy
COVID

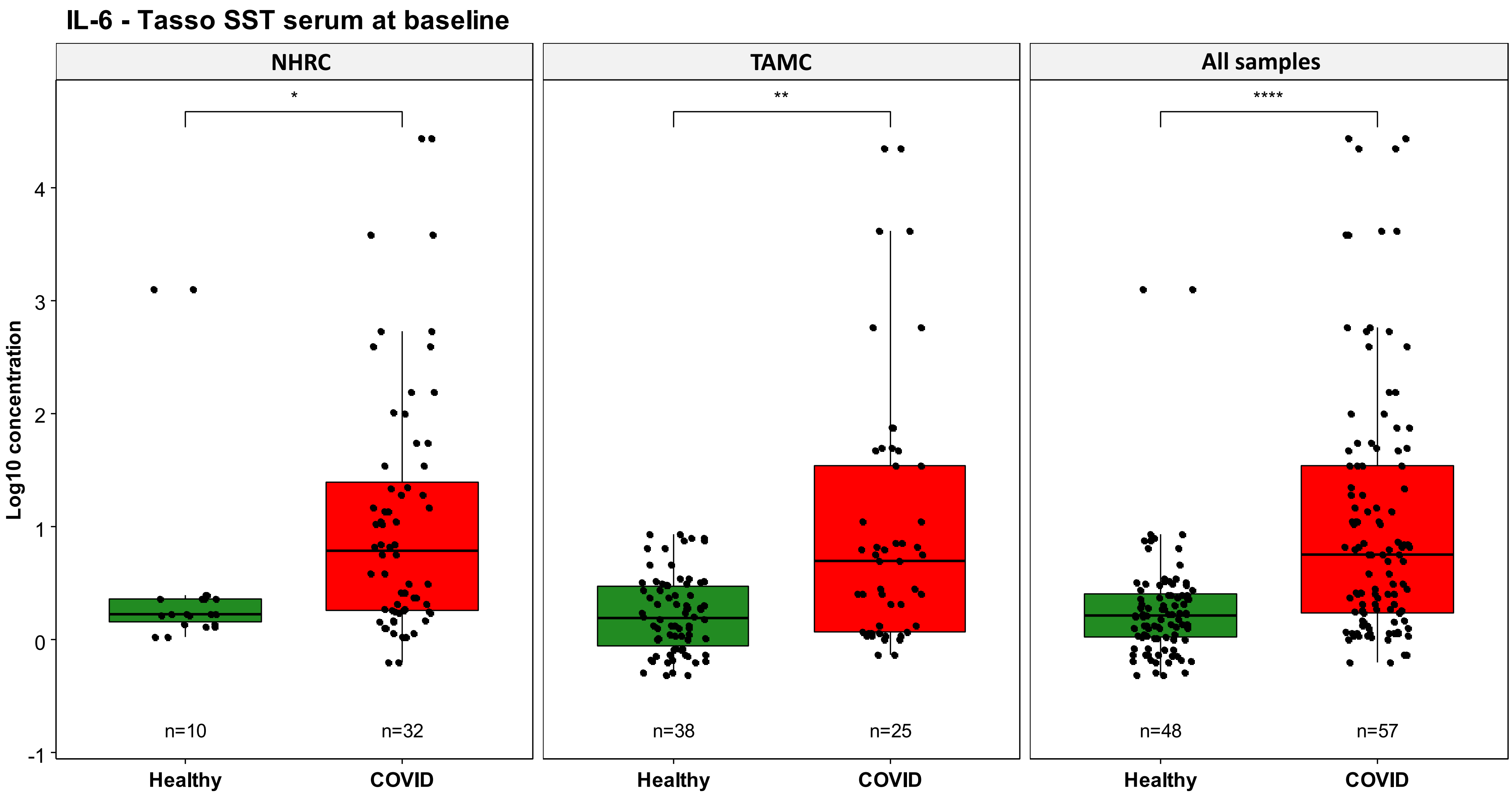

IL-6 - Tasso SST serum at baseline
NHRC
TAMC
All samples
*
**
****
Log10 concentration
4
3
2
1
0
-1
n=10
n=32
n=38
n=25
n=48
n=57
Healthy
COVID
Healthy
COVID
Healthy
COVID

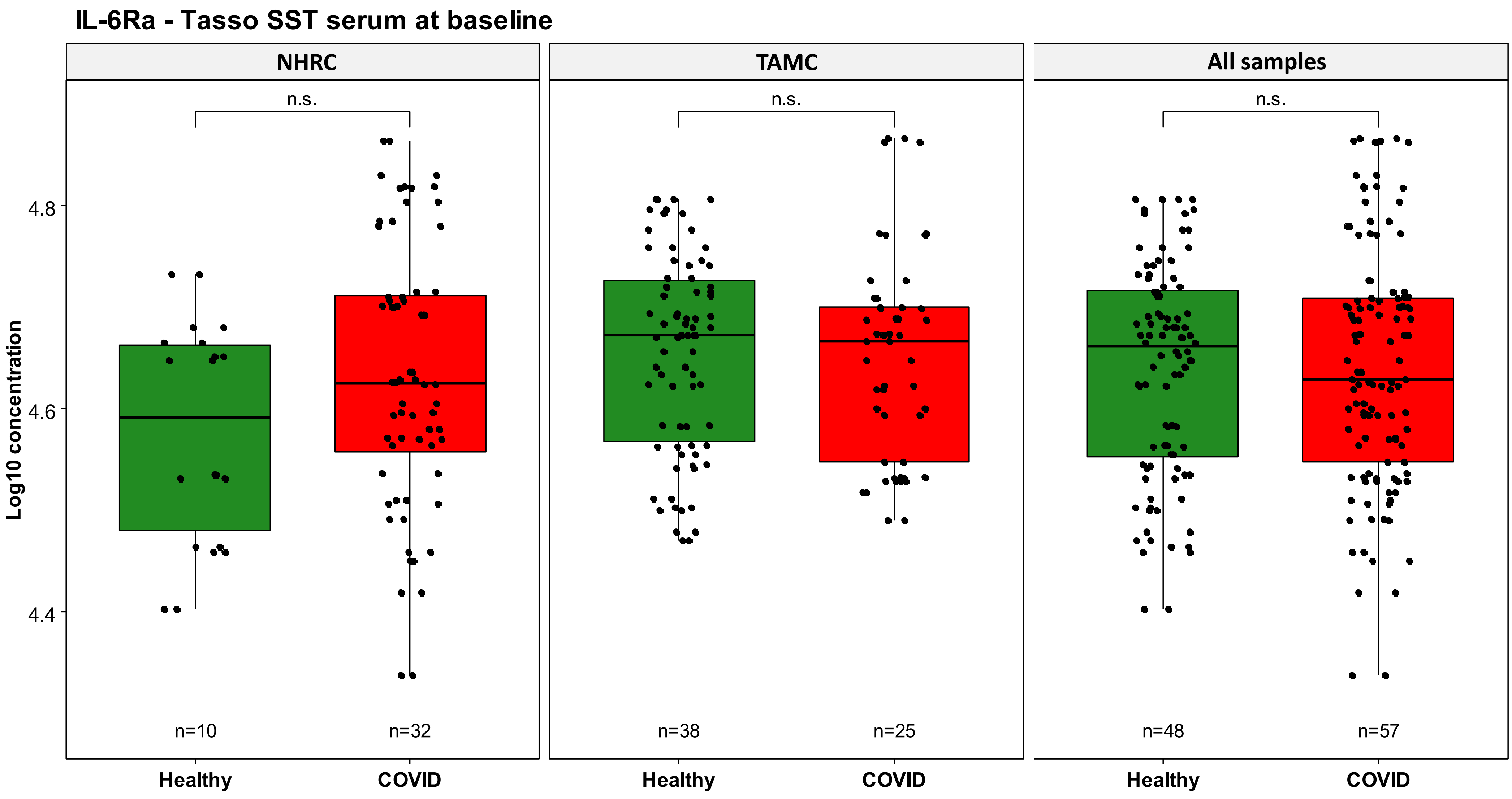

IL-6Ra - Tasso SST serum at baseline
NHRC
TAMC
All samples
n.s.
n.s.
n.s.
Log10 concentration
4.8
4.6
4.4
n=10
n=32
n=38
n=25
n=48
n=57
Healthy
COVID
Healthy
COVID
Healthy
COVID

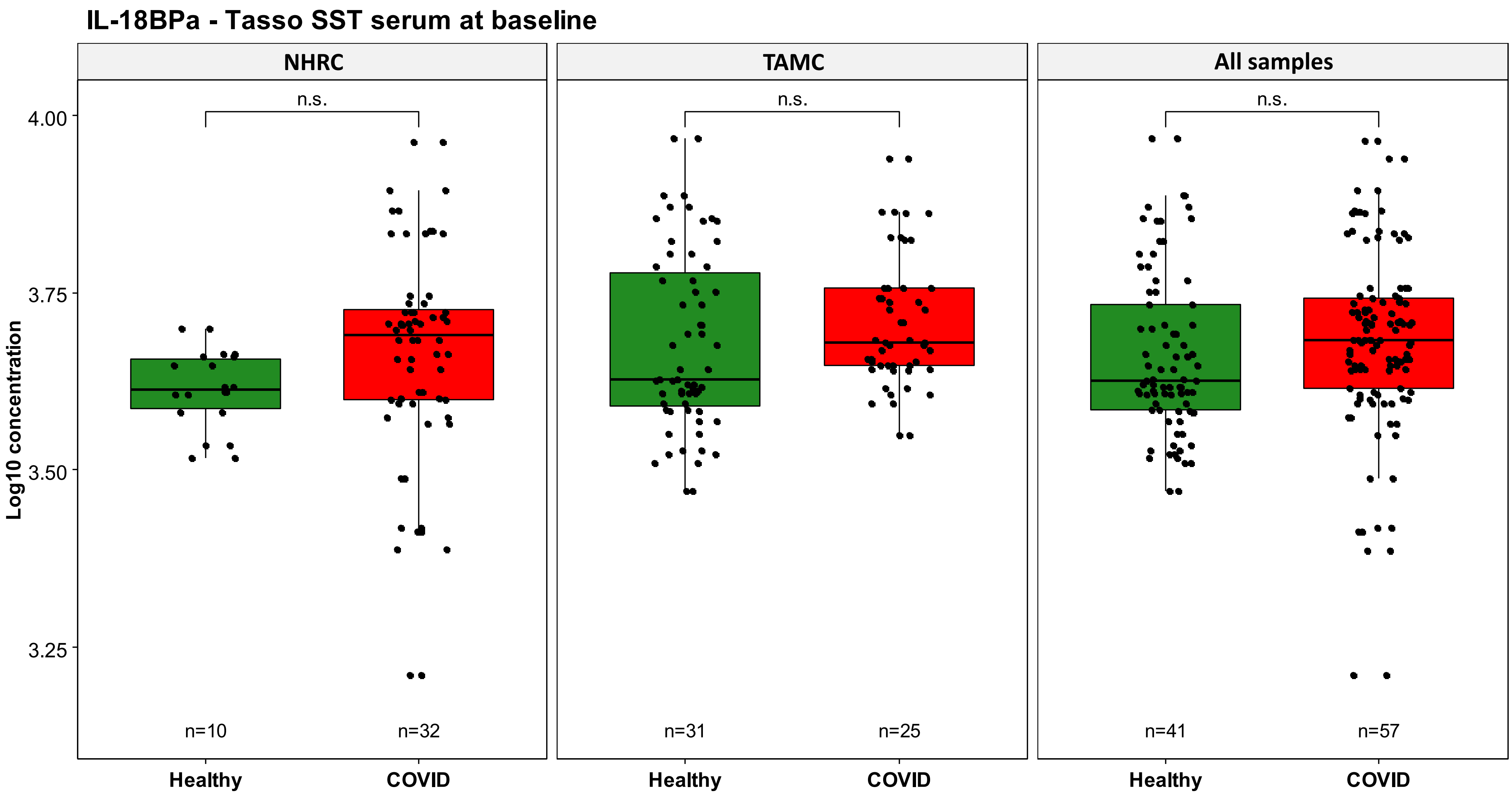

IL-18BPa - Tasso SST serum at baseline
NHRC
TAMC
All samples
n.s.
n.s.
n.s.
Log10 concentration
4.00
3.75
3.50
3.25
n=10
n=32
n=31
n=25
n=41
n=57
Healthy
COVID
Healthy
COVID
Healthy
COVID

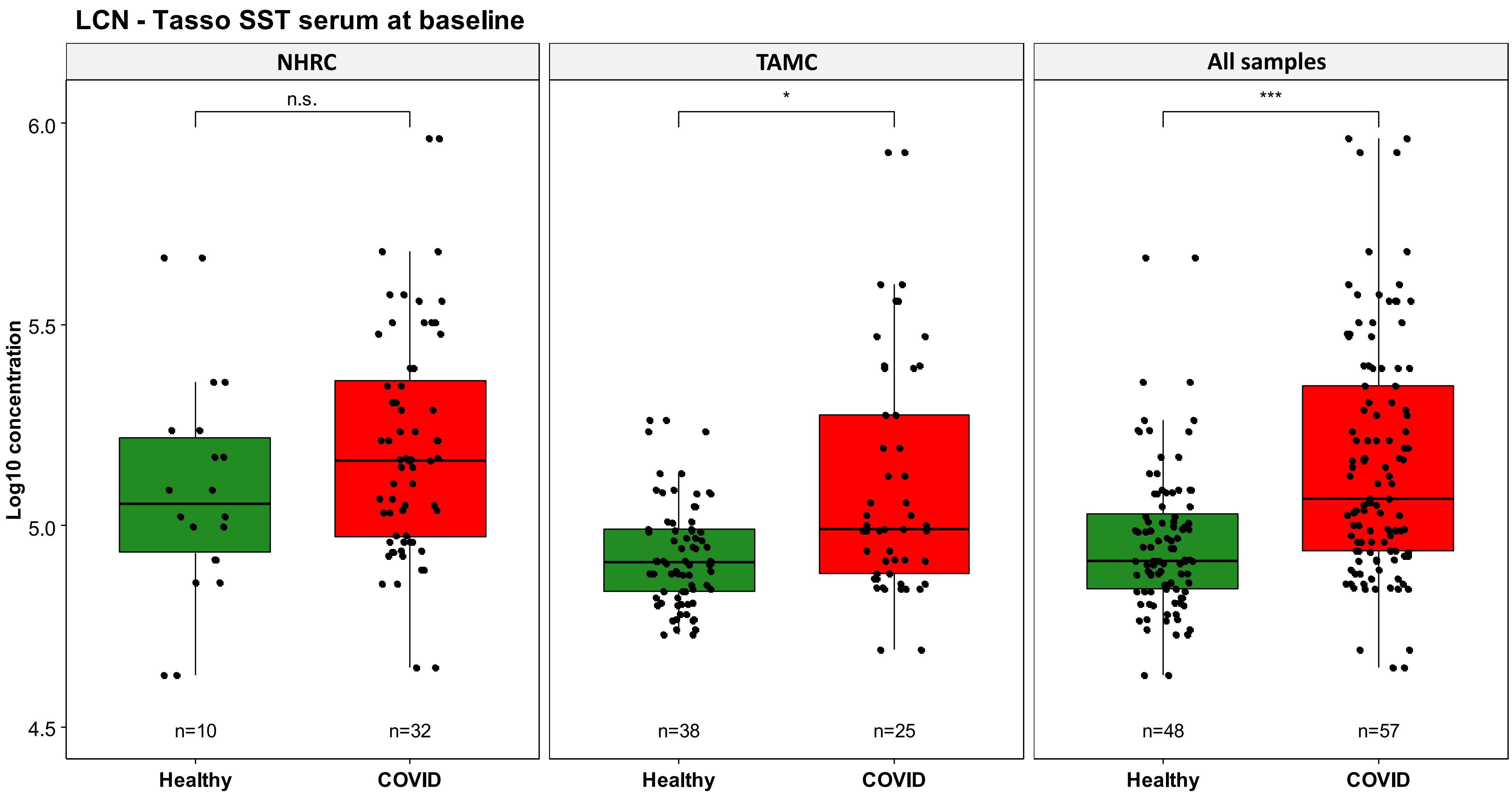

LCN - Tasso SST serum at baseline
NHRC
TAMC
All samples
n.s.
*
***
Log10 concentration
6.0
5.5
5.0
4.5
n=10
n=32
n=38
n=25
n=48
n=57
Healthy
COVID
Healthy
COVID
Healthy
COVID

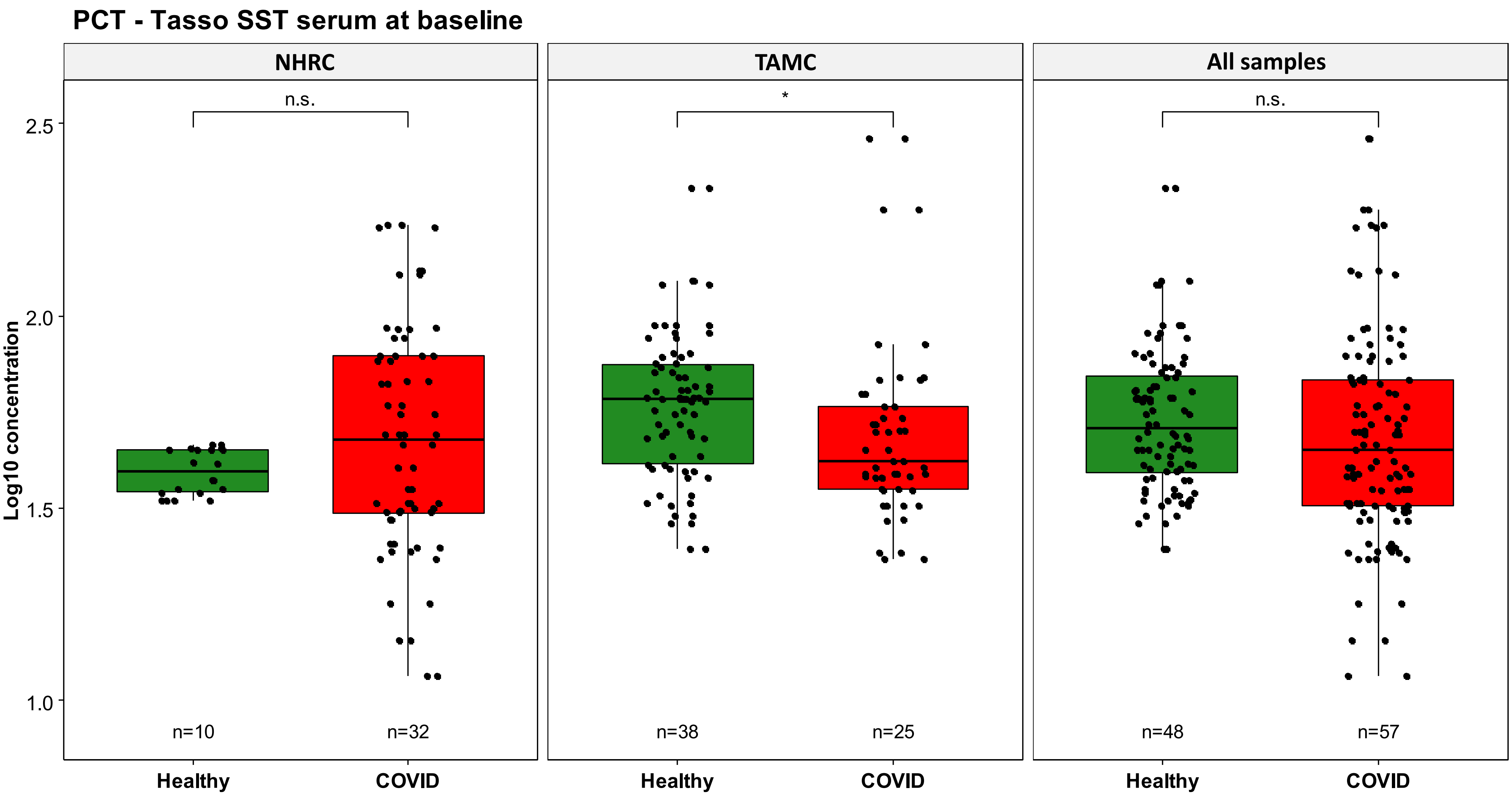
PCT - Tasso SST serum at baseline
NHRC
TAMC
All samples
n.s.
*
n.s.
Log10 concentration
2.5
2.0
1.5
1.0
n=10
n=32
n=38
n=25
n=48
n=57
Healthy
COVID
Healthy
COVID
Healthy
COVID

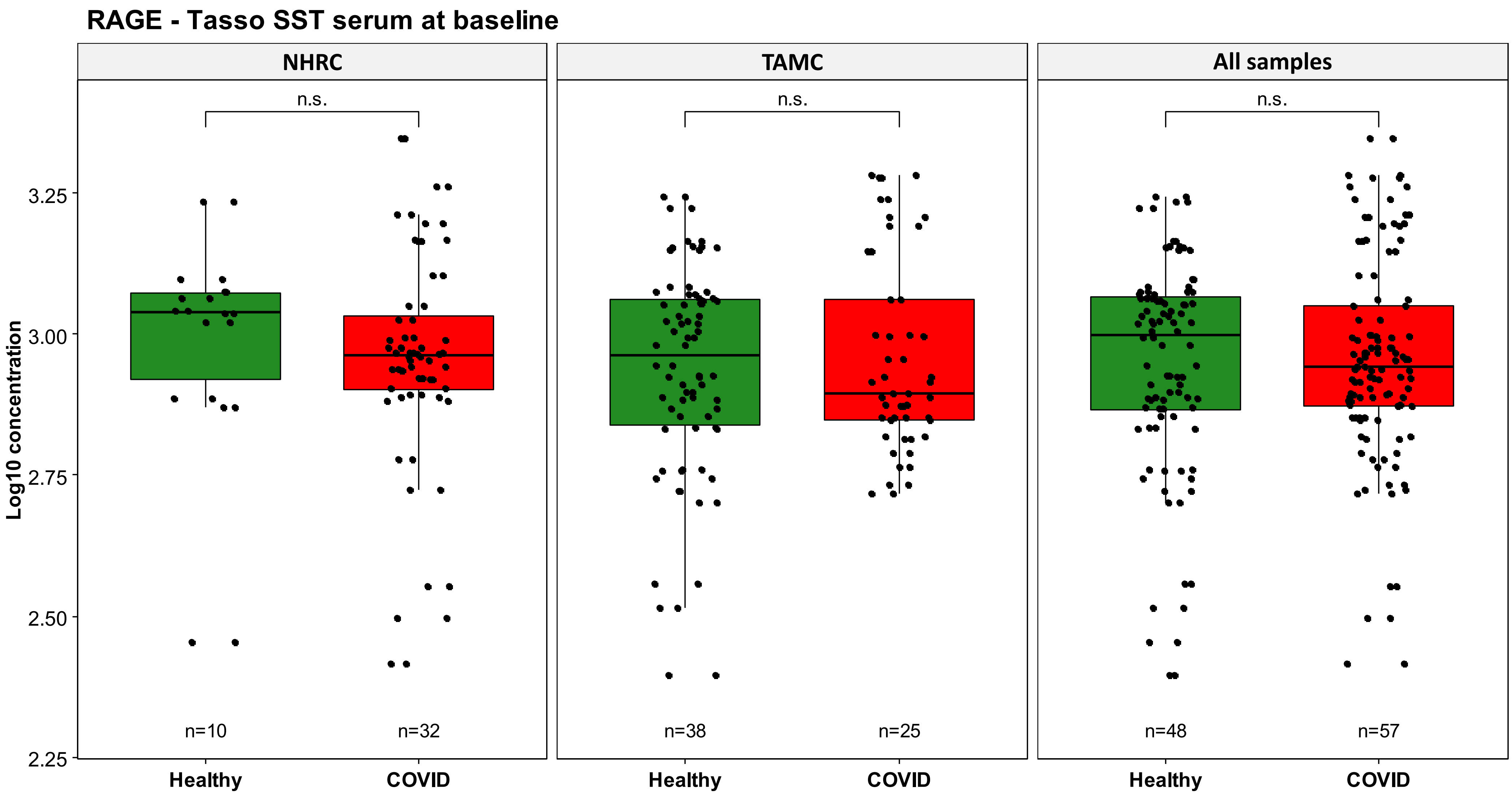

RAGE - Tasso SST serum at baseline
NHRC
TAMC
All samples
n.s.
n.s.
n.s.
Log10 concentration
3.25
3.00
2.75
2.50
2.25
n=10
n=32
n=38
n=25
n=48
n=57
Healthy
COVID
Healthy
COVID
Healthy
COVID

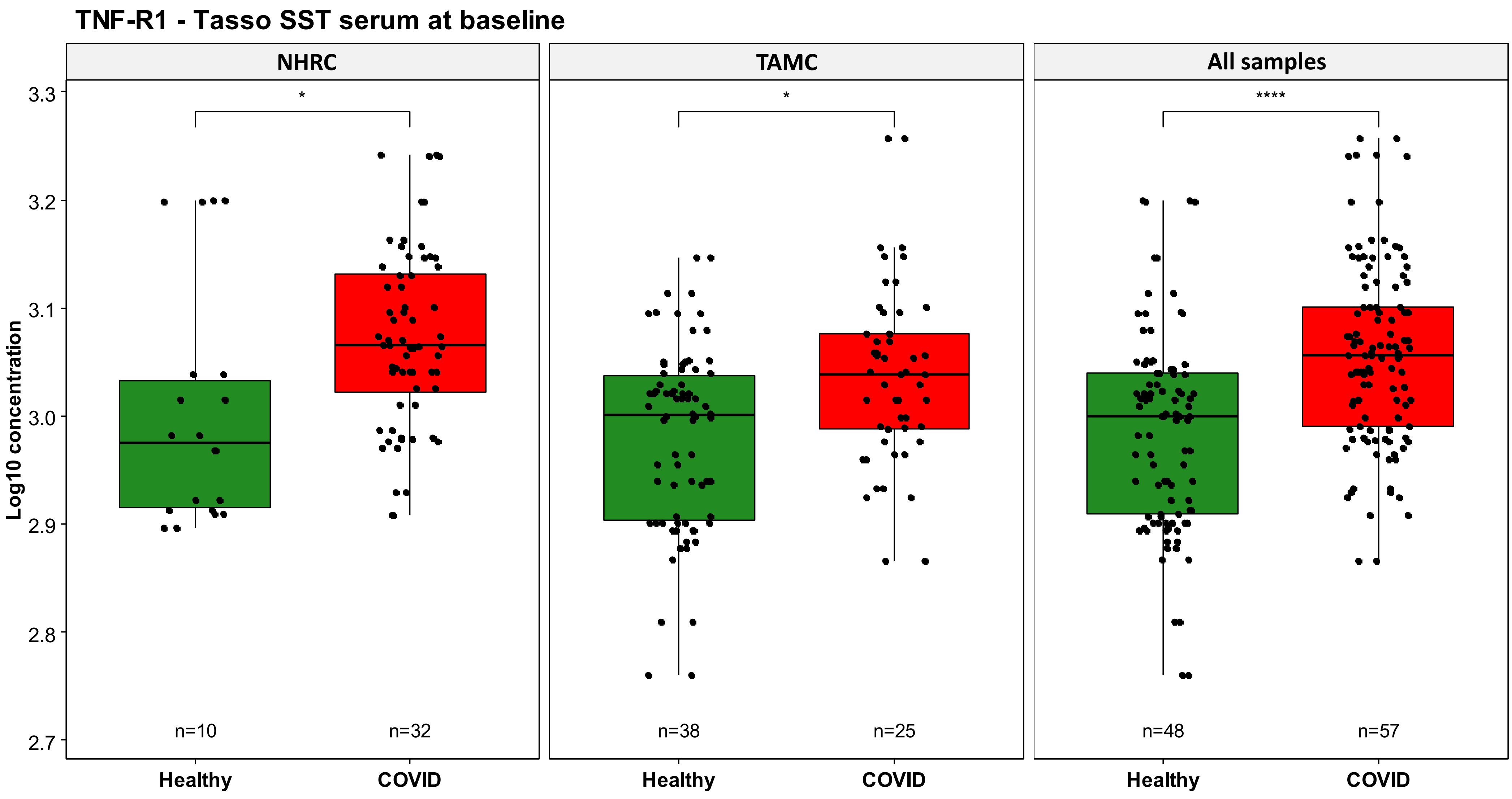

TNF-R1 - Tasso SST serum at baseline
NHRC
TAMC
All samples
*
*
****
Log10 concentration
3.3
3.2
3.1
3.0
2.9
2.8
2.7
n=10
n=32
n=38
n=25
n=48
n=57
Healthy
COVID
Healthy
COVID
Healthy
COVID

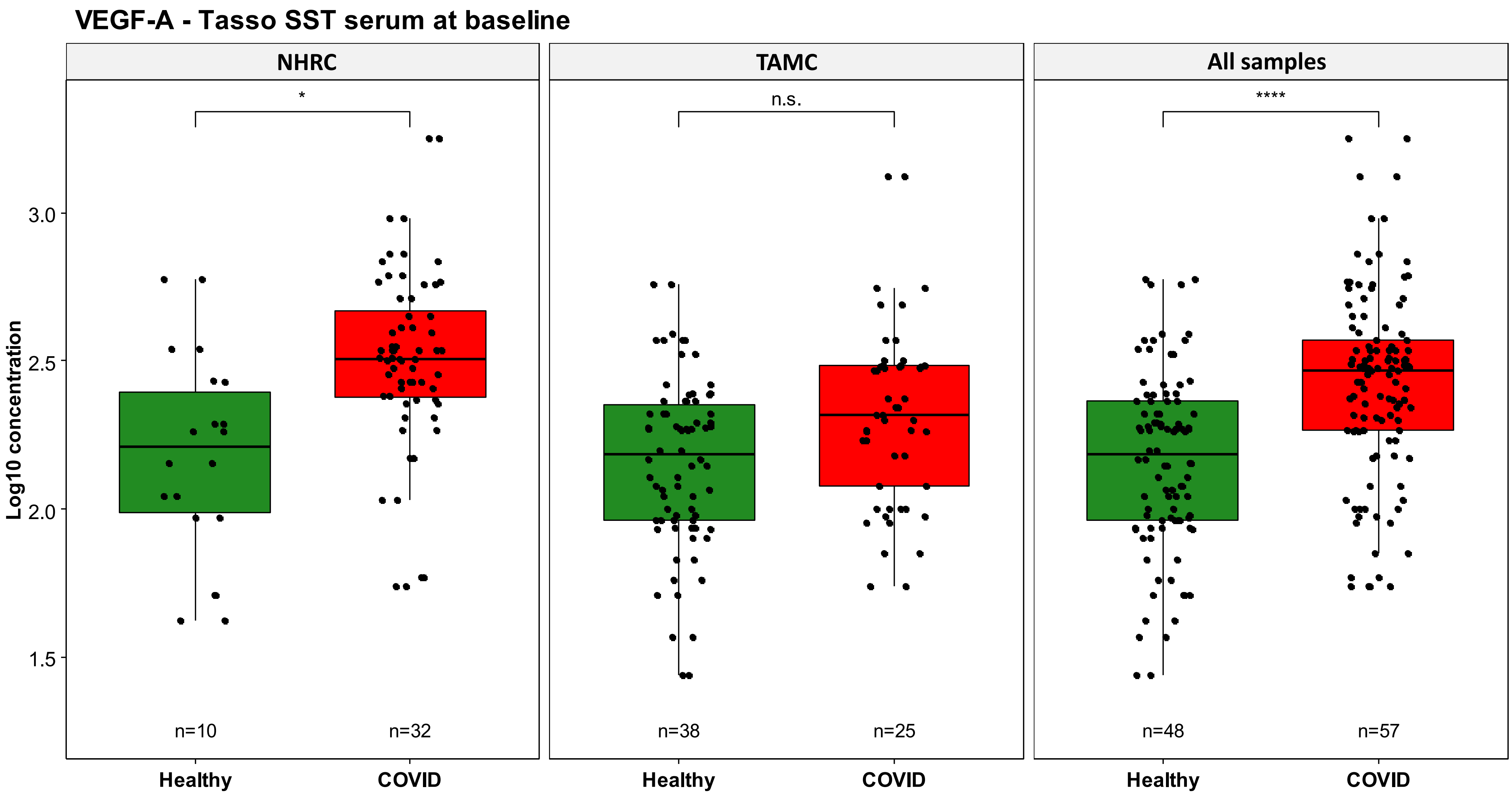
VEGF-A - Tasso SST serum at baseline
NHRC
TAMC
All samples
*
n.s.
****
3.0
2.5
2.0
1.5
Log10 concentration
n=10
n=32
n=38
n=25
n=48
n=57
Healthy
COVID
Healthy
COVID
Healthy
COVID
